# QTL mapping and genomic selection of stem and branch diameter in soybean (*Glycine max* L.)

**DOI:** 10.3389/fpls.2024.1388365

**Published:** 2024-05-31

**Authors:** Jing Wang, Qichao Yang, Yijie Chen, Kanglin Liu, Zhiqing Zhang, Yajun Xiong, Huan Yu, Yingdong Yu, Jun Wang, Jian Song, Lijuan Qiu

**Affiliations:** ^1^ MARA Key Laboratory of Sustainable Crop Production in the Middle Reaches of the Yangtze River (Co-construction by Ministry and Province), College of Agriculture, Yangtze University, Jingzhou, China; ^2^ The Shennong Laboratory, Zhengzhou, Henan, China; ^3^ State Key Laboratory of Crop Gene Resources and Breeding, Institute of Crop Sciences, Chinese Academy of Agricultural Sciences, Beijing, China; ^4^ National Key Facility for Gene Resources and Genetic Improvement, Key Laboratory of Crop Germplasm Utilization, Ministry of Agriculture and Rural Affairs, Institute of Crop Sciences, Chinese Academy of Agricultural Sciences, Beijing, China

**Keywords:** soybean, stem diameter, branch diameter, QTL, genomic selection

## Abstract

**Introduction:**

Soybean stem diameter (SD) and branch diameter (BD) are closely related traits, and genetic clarification of SD and BD is crucial for soybean breeding.

**Methods:**

SD and BD were genetically analyzed by a population of 363 RIL derived from the cross between Zhongdou41 (ZD41) and ZYD02878 using restricted two-stage multi-locus genome-wide association, inclusive composite interval mapping, and three-variance component multi-locus random SNP effect mixed linear modeling. Then candidate genes of major QTLs were selected and genetic selection model of SD and BD were constructed respectively.

**Results and discussion:**

The results showed that SD and BD were significantly correlated (r = 0.74, P < 0.001). A total of 93 and 84 unique quantitative trait loci (QTL) were detected for SD and BD, respectively by three different methods. There were two and ten major QTLs for SD and BD, respectively, with phenotypic variance explained (PVE) by more than 10%. Within these loci, seven genes involved in the regulation of phytohormones (IAA and GA) and cell proliferation and showing extensive expression of shoot apical meristematic genes were selected as candidate genes. Genomic selection (GS) analysis showed that the trait-associated markers identified in this study reached 0.47-0.73 in terms of prediction accuracy, which was enhanced by 6.56-23.69% compared with genome-wide markers. These results clarify the genetic basis of SD and BD, which laid solid foundation in regulation gene cloning, and GS models constructed could be potentially applied in future breeding programs.

## 1 Introduction

Soybeans are among the most important oil and protein sources worldwide (Zhang et al., 2022). Owing to the limited arable land and planting area of soybeans in China, increasing the yield is significant for meeting the increasing demand for soybean consumption (Sun and Li, 2015). Yield is a complex trait controlled by multiple genes in many crops, including soybean (Vogel et al., 2021). The stem provides mechanical support for the plant canopy (leaves, pods, and seeds) and transport systems for water, mineral elements, and carbohydrates via photosynthesis. Stem diameter (SD) is a vital factor influencing lodging, which can severely cause yield loss; when SD increases, the lodging ratio decreases significantly (Zhang et al., 2016). The SD and yield were closely correlated (r = 0.20–0.73) (Du and Wang, 2013). Branches developed from axillary buds on the main stem were also positively correlated with yield (Qiao et al., 2016). Branch number not only affects fruit setting but also affects plant light energy utilization (Xu et al., 2021). High-yield soybean plant architecture is also closely related to SD and branching traits, and the correlation coefficient between branch diameter (BD) and single plant yield is 0.30 (Yuan, 2015), however, there is relatively little research on the correlation between SD and BD.

Genetic analysis of SD revealed that SD was controlled by a batch of different QTLs. Using 257 soybean accessions, generalized linear model (GLM) and enriched compressed mixed linear model (E-cMLM) revealed that satt382 (A1) and satt534 (B2) with the highest positive and negative additive effects, respectively, were associated with SD (Yang, 2011). Five SD-associated QTLs (*qSD-1–1*, *qSD-8–1*, *qSD-8–2*, *qSD-24–1*, and *qSD-24–2*), located on the A2, C2, and L linkage groups, with phenotypic variance explained by 8.7–17.0%, were identified using composite interval mapping (CIM) based on 165 materials from the soy01 population (Zhou et al., 2009). In addition, ten SD QTLs located in the A1, B1, C2, D1a, E, and G linkage groups were identified in a recombinant inbred line (RIL) population (Charleston × Dongnong 594) consisting of 147 individual lines from different environments, with phenotypic variance explained of 7.0–38.5% (Fan et al., 2012). Among these 10 QTLs, *qST-C2–1* was adjacent to *qSD-8–1*, as mapped by Zhou et al. (2009). Based on an RIL population derived from the cross between “KeFeng 1” and “NanNong 1138–2”, an SD related QTL (*qSTD-13*) on chromosome 13, demonstrated a variance of 8.10% phenotypically (Hu, 2013). BARC-044739–08781 (D1b) was identified as an SD associated with a PVE of 6% using 219 soybean accessions by TASSEL (Zhang et al., 2015). Nine stable loci located on chromosomes 2, 5, 10, 14, 15, and 18, were identified to be associated with SD by GLM and a mixed linear model (MLM) based on 224 soybean microcore germplasm accessions. Of these nine loci, Map-1899 on chromosome 10 showed the strongest association with SD (Zhang, 2017). In addition, 20 single nucleotide polymorphism (SNP) loci significantly associated with SD were detected on chromosomes 2, 3, 4, 6, 8, 10, 11, 15, 16, and 19 of the 150 soybean accessions using restricted two-stage multi-locus genome-wide association (RTM-GWAS) (Li et al., 2021). Through three RIL populations (Nannong 94–156 × Bogao, Dongnong 50 × Williams 82, and Suinong 14 × Enrei) and inclusive composite interval mapping (ICIM), 12 SD QTLs were identified, of which *q11* was the most stable, explaining 12.58–26.63% of the phenotypic variation (Sun et al., 2021). Although extensive attention has been paid to genetic studies on SD, BD has not yet been fully investigated.

Genomic selection uses high-density markers across an entire genome for selective breeding (Meuwissen et al., 2001). The effect value of each marker was estimated using genotypes and phenotypes from the training population, and then the effect values of all markers were summed with only the genotypes to obtain the genomic estimated breeding value (GEBV) of the test individuals (Crossa et al., 2017). Owing to its short cycle, efficiency, and low cost, GS has been widely applied to many different crops, e.g. rice, maize, soybean (Liu et al., 2022). A genomic selection study of 10 agronomic traits in a population of 1495 rice hybrid combinations using genomic best linear unbiased prediction (GBLUP) showed that the prediction accuracy of seven traits exceeded 0.60 (Cui et al., 2020). Using ridge regression best linear unbiased prediction (rrBLUP) for genomic selection of maturity, plant height and seed weight in soybean, which can improve soybean breeding efficiency (Waltram et al., 2021). In maize, genomic selection was performed on southern corn rust resistance, and achieved prediction accuracy of 0.56–0.60 with GBLUP (Li et al., 2023a). To obtain a better performance, a statistical model is crucial for the prediction accuracy of genomic selection, e.g. GBLUP, rrBLUP, BayesA, Bayes B, Bayes C, and Bayes Lasso (Yin et al., 2019, Li et al., 2023b). The GBLUP was proposed by VanRaden in 2008 (VanRaden, 2008), has been widely applied owing to its high efficiency and robustness.

In this study, three different methods were used for QTL mapping of SD and BD in different environments and candidate genes of important QTL loci were analyzed as well. Besides, a genomic selection model was constructed for both SD and BD. This study provides a solid theoretical basis for the cloning of SD- and BD-regulating genes, and sheds light on marker selection strategies for genomic selection that could be further applied in breeding.

## 2 Materials and methods

### 2.1 Plant materials

An RIL population of 363 individual lines was obtained from the cross of ZD41 and ZYD02878 (wild soybean). The ZD41 is a summer soybean variety of high-yield and moderate maturing selected by the Oil Crops Research Institute of CAAS (Chinese Academy of Agricultural Sciences) from the cross between ZhongDou32 and Dundou. The ZYD02878 is a wild accession from Ningwu County, Shanxi Province deposited in Chinese Soybean Germplasm Resources Bank. ZD41 was phenotyped with erect stem, higher bottom pods and larger 100-seed weight, while stem of ZYD02878 was slender, and with more and longer branches, winding growth habit, more pods and smaller 100-seed weight.

The F_6_ and F_8_ of the RIL population were planted in Jingzhou (30.37°N, 112.06°E) and Sanya (18.25°N, 109.51°E) in 2019 (Chen et al., 2023), F_9_ was planted in the summer of 2021 at Ajian Farm, Zhengji Township, Yucheng County, Shangqiu City, Henan Province of China (34.41°N, 115.98°E). The RIL populations planted in different environments were abbreviated as 2019JZ (F_6_), 2019SY (F_7_), 2020JZ (F_8_), and 2021SQ (F_9_). The experiment was conducted using an interval contrast design and three technical replications were phenotyped in all environments except for 2019JZ with one replication, and field rows were all spaced by one meter to guarantee the growth space for each individual plant.

Plants were harvested at maturity for phenotyping. Effective branch was defined as the first level branch with more than two nodes and at least one pod, BD was defined as the diameter of the first internode of each effective branches and SD was defined as the diameter of the fifth internode of the main stem (Qiu and Chang, 2006). Phenotype data were collected for SD in four different environments, and BD from 2019JZ, 2019SY, and 2020JZ using a Vernier scale, with an accuracy of 0.1 mm.

### 2.2 Statistical analysis

Mean value was calculated after removing the outlier using the 1.5×interquartile range (IQR) (Ghasemi and Zahediasl, 2012) method and 3-σ principle (Khan et al., 2021) simultaneously. Descriptive statistics was performed using R package “psych.” (Revelle, 2015). Pearson correlation coefficient was calculated using R package “Hmisc.” (Harrell, 2019). Best linear unbiased estimates (BLUEs) was obtained via a mixed linear model assuming genotype as fixed effects and environments as random effects using R package “lme4” (Bates et al., 2015; Kibe et al., 2020). Mixed linear model was described by the formula as following:


Y=μ+ G+GxE+E+error

Where Y is represents observation value of each genotype in different environments, error is residual, μ is mean, G is fixed effect of genotype, E is random effect of environment, GxE is interaction effect between genotype and environment.

The broad-sense heritability (*h^2^*) of SD and BD was calculated according to the following equation:


$$h^{2}=\frac{V_{G}}{V_{G}+\frac{V_{GL}}{L}+\frac{V_{E}}{LR}}$$

where $V_{G}$ is the genotype variance, $\;V_{GL}$ is the two-level interaction variance of genotype and location; $V_{E}$ is the error variance; L is the number of locations; and R is the number of replicates. A full random model applied in lme4 was used to calculate broad sense heritability (Bates et al., 2015).

### 2.3 Genotyping and QTL analysis

Total DNA from each RIL was extracted from young fresh leaves using the CTAB method (Doyle and Doyle, 1990). A total of 158,290 SNP was obtained using Illumina soybean- SNP array (Beijing Compass Biotechnology Co., Ltd) (Sun et al., 2022). Then 127,185 SNP was obtained after filtration by MAF ≥ 0.005 and geno ≤ 0.2 (Purcell et al., 2007). After allele frequency distortion test with the expected ratio of 1:1 in terms of major allele to minor allele (Chi-test P >0.05), 41,994 SNP retained and subsequently resulted in 6098 bin markers using SNPBinner (Gonda et al., 2019).

The inclusive composite interval mapping (ICIM) showed excellent performance in background controlling in additive, dominant QTLs, as well as epistatic QTLs identification (Wang, 2009). The restricted two-stage multi-locus genome-wide association (RTM-GWAS) utilize SNPLDB harboring multi-allelic variation and trait heritability as the upper limit to detect as much QTLs as possible (He et al., 2017). The three-variance component multi-locus random SNP effect mixed linear model (3VmrMLM) was performed for association analysis as well (Li et al., 2022). To identify both major QTLs and minor effect QTLs, those three different methods for QTL mapping were performed for each environment separately and also for the combined environment using BLUE and the mean were adopted to explore the genetic mechanism underlie SD and BD based on bin markers.

The ICIM method was performed using QTL IciMapping 4.2 for QTL mapping according to the manufacturer’s instructions. A significant QTL was defined using an LOD threshold of 2.5. In RTM-GWAS pipeline, 4715 SNP linkage disequilibrium blocks (SNPLDBs) were first constructed based on 6098 bin markers, and the eigenvectors of SNPLDB and the genetic similarity coefficient (GSC) matrix obtained based on the whole genome were computed. Subsequently, an association analysis was performed using the multi-allele model of multiple loci, and the significance level was set at 0.01. Although the 3VmrMLM method was used to build a multi-locus genetic model to identify QTLs associated with SD and BD, the “Single_env” parameter was specified for the QTL detection, and significant association was defined by P-value ≤ 0.01/m and LOD ≥ 3.00, where m is the total number of bin markers.

Afterwards, PLINK (Purcell et al., 2007) was used to calculate the linkage disequilibrium (LD) of all QTLs mapped by the three different methods, and QTL regions with LD > 0.9 were defined as collocated QTLs.

### 2.4 Candidate gene selection

Regions of overlapping, neighboring, co-localized, pleiotropic effects, and high PVE (>10%) QTLs obtained using different methods were selected for candidate gene identification. Genes within these regions were functionally annotated using Soybase (https://www.soybase.org/) and the Phytozome (https://phytozome.jgi.doe.gov/), and SNP variation in the coding regions of those candidate genes with mutation types of nonsynonymous, synonymous, stopgain, stoploss and alternative splicing were annotated. Based on the genotype of 2214 soybean germplasm resources (Li et al., 2023c), 1132 cultivated soybeans, 861 improved varieties, and 218 wild soybeans were selected, and the fixation index (*F_ST_*) was calculated using vcftools (0.1.13) (Danecek et al., 2011). Coding sequence regions with *F_ST_* > 0.6 were used to identify potential domestication genes (Song et al., 2013; Chen et al., 2023). Tissue-specific expression patterns of candidate genes were analyzed in cotyledons, embryos, flowers, leaves, roots, seeds, seed coats, seedlings, shoot apical meristems, shoot meristems, stems, and axillary meristems using a soybean transcriptome integrative dataset (Yu et al., 2022). Expression was normalized using log_10_ (x+1), where x indicates fragments per kilobase of transcript per million reads mapped. Heatmap of gene expression was performed using the R package “pheatmap” (Kolde, 2015).

### 2.5 Genomic selection

To compare the effects of trait-associated marker sets on prediction accuracy, three different marker sets, G1 (all 6098 bin markers), G2 (5841 bin markers not associated with SD and BD), and G3 (257 bin markers associated with SD and BD), were set up, and a G matrix was constructed using SNP information instead of a relationship matrix (A matrix) (VanRaden, 2008). Genomic selection was performed on the mean and BLUE values of SD and BD in each environment, and 80% and 20% of the total population lines were selected as the training and test sets, respectively, via five-fold cross-validation with 123 random seeds and 20 replications. The average of the prediction accuracies of SD and BD was regarded as the final prediction accuracy. The GBLUP model was constructed using the rrBLUP package in R.

## 3 Results

### 3.1 Phenotypic descriptive statistics

**Figure 1:**
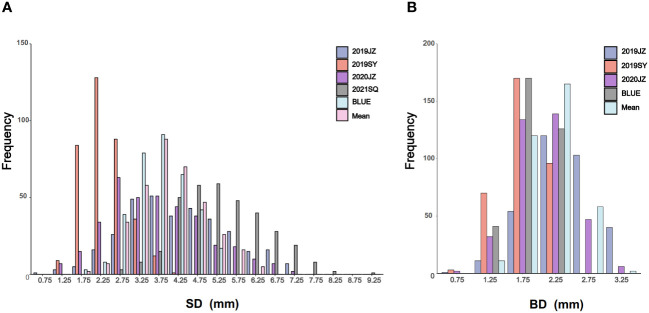
Phenotypic frequency distribution of SD and BD. **(A, B)** are the frequency distribution of SD and BD in different environments, BLUE, and mean; SD is stem diameter; BD is branch diameter; SY is Sanya; JZ is Jingzhou; SQ is Shangqiu; BLUE is the best linear unbiased estimate.

Outliers of SD and BD in RIL population in different environments were first filtrated using 1.5×IQR and 3-σ principle (**Table 1**). The SD of maternal parent ZD41 ranged from 4.08–9.05 mm, whereas that of paternal parent (wild soybean, ZYD02878) was significantly thinner (0.82–3.42 mm) in different environments. A similar trend was observed for BD ZD41 and ZYD02878 (**Table 1**; **Supplementary Table S1**). Generally, both SD and BD in the four different environments (2019JZ, 2019SY, 2020JZ, and 2021SQ) showed nearly normal distributions (**Table 1**; **Figure 1**), suggesting that both SD and BD were quantitative traits. However, the ranges differed in each environment (**Table 1**). Both SD and BD exhibited the widest range in 2019JZ and the smallest range in 2019SY (**Table 1**). Transgressive segregation was observed in both SD and BD in different environments, except in 2019SY-SD. In particular, 126 RILs exceeded the high-value parent (ZD41) in 2020JZ in terms of the SD. For BD, extensive transgression segregation was observed in the 2019SY, in which 20 and 47 parents exceeded the high- and low-value parents, respectively. More individuals exceeded high-value parents under SD than under BD.

**Table 1:** Descriptive statistics of SD and BD in different environments .

Trait	Environment	P1	P2	P1-P2	Max	Min	Mean	Range	SD^a^	Skewness	Kurtosis	Outlier	Transgressive Segregation with Respect to Higher Parent	Transgressive Segregation with Respect to Lower Parent.
SD	2019JZ	6.45	0.82	5.63	7.91	1.32	4.44	6.59	1.40	0.40	-0.28	14	35	0
	2019SY	4.13	1.03	3.10	3.84	1.10	2.38	2.74	0.54	0.48	-0.20	4	0	0
	2020JZ	4.08	1.08	3.01	7.07	1.24	3.72	5.83	1.25	0.47	-0.32	6	126	0
	2021SQ	9.05	3.42	5.63	8.16	2.65	5.40	5.51	1.07	0.24	-0.33	13	0	8
	BLUE	5.75	1.72	4.03	5.86	1.94	3.81	3.92	0.76	0.26	-0.29	13	1	0
	Mean	5.93	1.59	4.34	6.13	1.99	4.00	4.14	0.85	0.31	-0.28	12	7	1
BD	2019JZ	3.77	0.90	2.87	3.94	1.09	2.51	2.85	0.57	0.28	-0.22	9	6	0
	2019SY	2.49	1.45	1.04	2.89	0.71	1.83	2.18	0.39	0.27	-0.18	3	20	47
	2020JZ	2.59	0.93	1.66	3.20	0.90	2.06	2.30	0.43	0.20	-0.20	3	42	1
	BLUE	2.63	1.01	1.62	2.77	1.09	1.93	1.68	0.33	0.08	-0.28	11	6	0
	Mean	2.95	1.09	1.86	3.02	1.21	2.13	1.81	0.36	0.17	-0.28	9	4	0

SD, stem diameter; BD, branch diameter; P1, ZD41; P2, ZYD02878; JZ, Jingzhou; SY, Sanya; SQ, Shangqiu; BLUE, best linear unbiased estimate; SD^a^, standard deviation.

Correlation analysis showed that the SD and BD was significantly correlated in 2019JZ, 2019SY, and 2020JZ (r = 0.60–0.83, P< 0.001), which was higher than correlation between either SD–SD or BD–BD between different environments (**Table 2**; **Supplementary Table S2**). The broad-sense heritability of SD was 0.70, which was higher than that of BD (0.57) (**Table 3**).

**Table 2:** Correlation analysis between SD and BD among different environments.

Trait	Environment	SD				BD			SD.BLUE	SD.mean
		2019JZ	2019SY	2020JZ	2021SQ	2019JZ	2019SY	2020JZ		
SD	2019JZ	1								
	2019SY	0.24^***^	1							
	2020JZ	0.47^***^	0.21^***^	1						
	2021SQ	0.38^***^	0.40^***^	0.47^***^	1					
BD	2019JZ	0.60^***^	0.26^***^	0.52^***^	0.44^***^	1				
	2019SY	0.13^**^	0.63^***^	0.16^**^	0.25^***^	0.21^***^	1			
	2020JZ	0.44^***^	0.27^***^	0.83^***^	0.46^***^	0.61^***^	0.27^***^	1		
BD.BLUE									0.74^***^	
BD.mean										0.75^***^

SD, stem diameter; BD, branch diameter; JZ, Jingzhou; SY, Sanya; SQ, Shangqiu; BLUE, best linear unbiased estimate; where * * * represents P<0.001 and * * represents P<0.05.

**Table 3:** Broad sense heritability estimation.

Trait	V_G_	V_L_	V_E_	V_GL_	V_GL_/L	V_E_/LR	*h^2^*
SD	0.55	2.44	0.88	0.40	0.13	0.10	0.70
BD	0.08	0.06	0.13	0.07	0.04	0.02	0.57

SD, stem diameter; BD, branch diameter; $V_{G}$ is the genotype variance; $V_{E}$ is the error variance; $V_{GL}$ is the two-level interaction variance of genotype; *h^2^*, broad sense heritability.

### 3.2 QTL identification

QTL mapping was performed using three different approaches: RTM-GWAS, ICIM, and 3VmrMLM for SD and BD. A total of 92, 36, and 17 QTLs were identified for SD and BD in the different environments using RTM-GWAS, ICIM, and 3VmrMLM, respectively (**Supplementary Table S3**). Using RTM-GWAS, 50 and 42 QTL were identified for SD and BD, respectively (**Supplementary Table S3**; **Supplementary Figure S1**). Of these QTLs, *qSD10–4* and *qBD10–1* showed the highest PVE, demonstrating phenotypic variance of 19.61% and 17.46%, respectively. In addition, *qSD8–2*, *qBD10–5*, *qBD11–4* were the major QTLs with a PVE > 10%. Only two QTLs (*qBD11–3* and *qBD11–5*) for SD in the different environments (2019JZ and 2020JZ) were localized adjacently (**Supplementary Table S4**). Eighteen QTLs were identified for SD and BD using ICIM (**Supplementary Table S3**). Of these QTLs, five had a PVE > 10%: *qSD10–9* (15.96%), *qBD3–4* (14.38%), *qBD4–6* (11.16%), *qBD10–10* (10.35%), and *qBD10–11* (10.11%). *qSD10–9* and *qBD10–10* were co-localized (Chr10: 45,243,194-45,322,107), with a maximum LOD of 24.09, indicating a possible pleiotropic locus. In addition, the co-localized QTLs *qBD1–1* and *qBD1–3* were persistently identified in 2019JZ and 2020JZ (**Supplementary Table S4**). Using 3VmrMLM, seven and ten QTLs were identified for SD and BD, respectively (**Supplementary Table S3**; **Supplementary Figure S2**). Only *qSD10–12* (12.60%), *qBD11–10* (11.24%), and *qBD10–15* (11.03%) demonstrated phenotypic variation >10%, *qBD10–15* and its co-localized QTL *qBD10–16* were mapped to both 2019JZ and 2020JZ (**Supplementary Table S4**). Taken together, 13 QTLs were possibly major QTL with high PVE in different environments, and one of these QTL loci (Chr10:45,243,194-45,257,940) were repeatedly identified in different environments (**Supplementary Table S3**).

**Figure 2:**
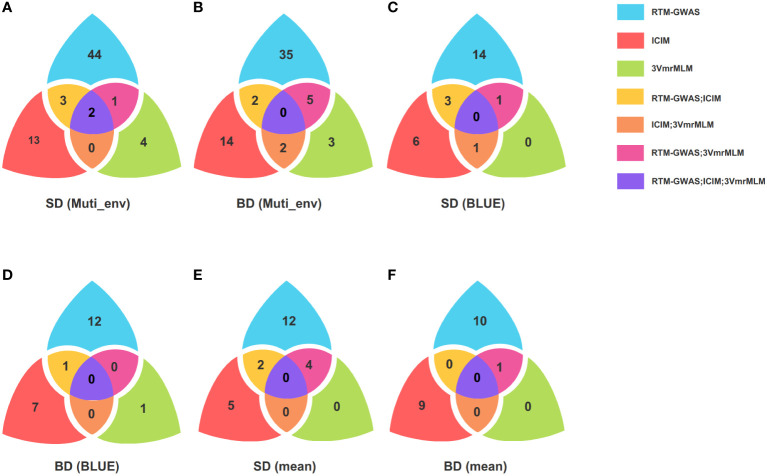
Venn plot of QTLs identified by multiple methods and colocalized QTL. **(A, B)** are QTL number identified by RTM-GWAS, ICIM, and 3VmrMLM for SD and BD in different environment; **(C, D)** are QTL number identified by RTM-GWAS, ICIM, and 3VmrMLM for SD and BD using BLUE; **(E, F)** are QTL number identified by ICIM, RTM-GWAS, and 3VmrMLM for SD and BD using mean; RTM-GWAS is the restricted two-stage multi-locus genome-wide association analysis; ICIM is the inclusive composite interval mapping; 3VmrMLM is the three variance component multi-locus random SNP effect mixed linear model analysis; SD is stem diameter; BD is branch diameter; BLUE is the best linear unbiased estimate; Muti_env represents different environments, including Jingzhou, Sanya and Shangqiu.

Among the four environments, the largest number of QTLs was identified in 2020JZ (49), followed by 2019SY (43) and 2019JZ (39), and only 14 QTLs were identified in 2021SQ (**Supplementary Table S3**). Fifteen loci were found in the same environment using different methods (**Supplementary Table S5**), of which 13 loci were detected using two different methods, whereas only two loci, namely Chr08: 46,364,044-46,518,573 in 2019SY and Chr10: 45,120,118-45,322,107 in 2020JZ, were detected using all three methods (**Figures 2A, B**).

A total of 30 SD and 22 BD QTLs were identified through RTM-GWAS, ICIM, and 3VmrMLM using BLUE (**Supplementary Table S3**), which were then compared with those identified in different environments. Fourteen co-localized loci were found to be distributed on chromosomes 1, 7, 8, 9, 10, 15, and 20. One BD (Chr20: 37,526,276-37,703,948) and three SD co-localized loci (Chr08: 46,364,044-46,420,859; Chr10: 45,120,118-45,322,107; Chr15: 11,778,040-11,883,830) were identified using RTM-GWAS and ICIM, one SD co-localized locus (Chr08: 46,364,044-46,420,859) was identified using ICIM and 3VmrMLM, and one SD co-localized locus (Chr10: 45,120,118-45,257,940) was identified using the RTM-GWAS and 3VmrMLM (**Figures 2C, D**). Based on the BLUE results, a region on chromosome 1 (Chr01: 3,561,373-3,678,971) was identified in 2019JZ and 2020JZ, and this locus was associated with BD. Both ICIM and 3VmrMLM revealed that the region on chromosome 8 (Chr08: 46,364,044-46,420,859) was associated with SD in 2019SY and BLUE. Another region on chromosome 10 (Chr10: 45,120,118-45,322,107) was identified using RTM-GWAS and ICIM in 2020JZ, and BLUE was associated with SD (**Supplementary Table S6**).

Using the mean as the phenotypic input, three different methods mapped 50 QTLs for SD and BD (**Supplementary Table S3**). Among them, RTM-GWAS and 3VmrMLM shared four loci associated with SD and one with BD, whereas two SD co-localized QTLs were found by RTM-GWAS with ICIM (**Figures 2E, F**). Comparing to the QTLs mapped from different environments, 14 co-localized QTLs were identified and distributed on chromosomes 2, 5, 6, 7, 8, 10, 13, and 18. Taking these results together, four co-localized loci were found in BLUE, mean, and different environments, namely Chr07: 13,790,127-14,357,932; Chr08: 7,225,100-7,322,137; Chr08: 46,364,044-46,518,573; Chr10: 45,120,118-45,322,107 (**Supplementary Table S6**).

Taken together, 93 SD-and 84 BD-associated loci were uniquely mapped to different chromosomes, and 35 of these loci were mapped by multiple environments or different methods (**Supplementary Table S7**).

### 3.3 Pleiotropic QTLs

In this study, 12 possibly pleiotropic QTLs were identified using three different methods in different environments, four pleiotropic QTLs were identified based on BLUE values using ICIM alone, and 4 pleiotropic QTLs were identified based on mean values using both ICIM and RTM-GWAS (**Supplementary Table S8**). In particular, a region of 201.9kb on chromosome 10 (Chr10: 45,120,118-45,322,107) was found to be associated with SD and BD in BLUE, mean, and different environments using ICIM, RTM-GWAS, and 3VmrMLM and explained a phenotypic variance of 7.22–19.61%, indicating that this locus was stable and reliable. In addition, although the PVE values of the two QTLs located on chromosome 8 (Chr08: 7,225,100-7,322,137; Chr08: 46,364,044-46,518,573) were low, these two QTLs were repeatedly identified using RTM-GWAS, ICIM, and 3VmrMLM and were associated with SD and BD simultaneously, suggesting that these two loci were also reliable pleiotropic QTLs. In addition, a region on chromosome 11 (Chr11: 10,779,406-11,043,519) was found to be associated with SD and BD and was persistently identified in different environments using RTM-GWAS and 3VmrMLM.

### 3.4 Candidate gene analysis

Based on genetic analysis, 247 QTLs were obtained using these three methods; of which, 26 QTL regions were identified with high PVE (>10%) and LD (>0.9), and 255 gene models within these regions were identified according to Wm82.a2.v1. After SNP variation analysis, 253 of these genes contained nonsynonymous, synonymous, stop-gain, stop-loss, and alternative splicing SNPs. Genetic differentiation analysis showed that the *F_ST_* in the CDS region of 92 genes was >0.6 (**Supplementary Table S9**; **Supplementary Figure S3**), suggesting that these genes might have been subjected to domestication selection. Gene expression pattern analysis demonstrated that 55 of these genes are expressed in the stem, shoot, apical meristem, and axillary meristem. Of these 55 genes, 46 were functionally annotated based on Phytozome and Soybase. In addition, based on gene function, five genes coding for uncharacterized proteins (**Supplementary Table S10**), and seven genes coding for the expression of shoot apical meristem, regulation of auxin (IAA) and gibberellin (GA), and cell proliferation (**Table 4**) might influence the growth and development of SD and BD.

**Table 4:** Functional annotation of 7 candidate genes related to SD and BD.

Trait	Soybean	Start	End	Arabidopsis	Annotation	Functional pathway
SD_BD	*Glyma.08G095800*	7,304,924	7,308,800	*AT2G01570*	GRAS family transcription factor family protein	cell proliferation and expansion
SD_BD	*Glyma.08G351900*	46,509,706	46,516,703	*AT5G59540*	2-oxoglutarate (2OG) and Fe(II)-dependent oxygenase superfamily protein	Plant hormone regulation
SD_BD	*Glyma.10G220700*	45,193,087	45,207,808	*AT1G34110*	Leucine-rich receptor-like protein kinase family protein	Expression of shoot apical meristematic
SD_BD	*Glyma.10G224200*	45,498,206	45,500,186	*AT1G22780*	Ribosomal protein S13/S18 family	Expression of shoot apical meristematic
SD_BD	*Glyma.11G141200*	10,783,630	10,788,473	*AT2G28760*	UDP-XYL synthase 6	Cell wall synthesis
SD_BD	*Glyma.11G141300*	10,790,718	10,795,368	*AT3G46440*	UDP-XYL synthase 5	Cell wall synthesis
SD_BD	*Glyma.15G143700*	11,812,631	11,819,435	*AT5G49360*	Beta-xylosidase 1	Secondary cell wall synthesis

SD, stem diameter; BD, branch diameter; SD_BD, pleiotropic.

### 3.5 Genomic selection

Genome-wide selection was performed using BLUE, mean, and phenotypic values of each environment of SD and BD using three different marker sets (G1–G3), and the results showed that the maximum prediction accuracy of SD and BD both reached 0.73. Regarding SD, the highest prediction accuracy was observed in the G3 marker sets (trait-associated markers), followed by G1 and G2. The prediction accuracy of G2 (5841 bin markers unassociated with SD and BD) ranged from 0.37–0.66, and when genome-wide bin markers were used (G1), the prediction accuracy was significantly improved by 1.52–4.35%. However, a significant improvement of 8.96–23.68% was observed compared with the G3 marker set. In particular, the BLUE and mean values (averaged at 0.67) showed better performance in the GS than in each environment (averaged at 0.51) in terms of prediction accuracy (**Table 5**). A similar trend was observed for the GS in the BD group. Genome-wide bin markers (G1) showed a prediction accuracy ranging from 1.64–4.08% and outperformed the trait unassociated marker set (G2). Further, the G3 marker set improved the prediction accuracy by 6.56–21.57% compared with G1. Both BLUE and mean values showed a higher prediction accuracy (0.67) than each environment (0.6) (**Table 5**). However, BLUE and the mean values showed opposite trends in SD and BD; BLUE showed higher prediction accuracy than the mean by 4.29–6.45% in SD, whereas it was lower than the mean by 1.39–1.59% in BD. Notably, BD demonstrated a higher prediction accuracy than SD in 2019JZ, 2019SY, and 2020JZ (**Table 5**).

**Table 5:** GS prediction accuracy estimated by three marker sets.

Maker sets	GS prediction accuracy (SD)						GS prediction accuracy (BD)				
	2019JZ	2019SY	2020JZ	2021SQ	BLUE	Mean	2019JZ	2019SY	2020JZ	BLUE	Mean
G1	0.38	0.52	0.58	0.48	0.67	0.64	0.51	0.61	0.62	0.65	0.66
G2	0.37	0.51	0.57	0.46	0.66	0.62	0.49	0.60	0.61	0.63	0.64
G3	0.47	0.57	0.65	0.55	0.73	0.70	0.62	0.65	0.69	0.72	0.73

SD, stem diameter; BD, branch diameter; JZ, Jingzhou; SY, Sanya; SQ, Shangqiu; BLUE, best linear unbiased estimate; G1, all 6098 bin markers; G2, 5841 bin markers unassociated with SD and BD; G3, 257 bin markers associated to SD and BD.

## 4 Discussion

### 4.1 Combination of different methods increased the reliability of QTL mapping

Complex quantitative traits in soybeans are usually interrelated (Berhanu et al., 2021) and influenced by both genetic and environmental factors (Wang et al., 2019). The QTL mapping efficiency and accuracy of quantitative traits could be largely influenced by the application in a single environment. In this study, QTL mapping of SD and BD was performed in multiple environments (19JZ, 19SY, 20JZ, and 21SQ) to reduce environmental influences. In addition, different QTL mapping results are usually observed in various methods owing to the different assumptions and models applied, and each method has its advantages and disadvantages. To this end, three different methods, namely RTM-GWAS, ICIM, and 3VmrMLM, were adopted simultaneously in this study to achieve a holographic genetic scene of SD and BD.

In this study, 247 QTLs were identified, of which 152, 70, and 25 were identified using RTM-GWAS, ICIM, and 3VmrMLM, respectively (**Supplementary Table S3**). This result is consistent with the fact that a greater number of QTLs were identified by RTM-GWAS than by other methods (Gai and He, 2020; Pan et al., 2020). In this study, the repeatability of QTL detected in different environments or by different methods was relatively low. This is probably because that the SD and BD were two traits of relatively low heritability, which indicating the interaction between genotype and environments is non-ignorable. Although there are fewer QTLs co-localized in different environments, these QTLs are also very stable and reliable. Previously 12 SD QTL in three RIL populations and five environments were identified (Sun et al., 2021), of which *q11* was mapped to Chr11: 10,875,976-24,450,687, within this region five SD related QTLs (*qSD11–4*, *qSD11–5*, *qSD11–6*, *qSD11–7* and *qSD11–9*) and two BD QTLs (*qBD11–3* and *qBD11–9*) were detected in this study. In addition, there were other trait-associated QTLs in this region, such as *Seed weight 2-g3* (Zhang et al., 2015), *Seed weight 13-g1* (Wang et al., 2016), *Plant height 3*, and *Pod number 2* (Contreras-Soto et al., 2017), indicating that Chr11: 10,875,976-24,450,687 may be possibly pleiotropic not only to SD and BD but also to regulate plant height, seed weight, and pod number.

### 4.2 Candidate gene prediction

To narrow the candidate gene list from the stable and reliable QTL regions identified using these three methods, SNP variant analysis, genetic differentiation index analysis, and calculation of candidate gene expression were performed, and seven candidate genes were obtained (**Table 4**). Of which *Glyma.08G095800* encodes a GRAS transcription factor, a member of the VHIID/DELLA regulatory family, involved in the inhibition of cell proliferation and expansion in response to gibberellin degradation, thereby promoting plant growth (Hirsch and Oldroyd, 2009). *Glyma.08G351900* encodes a 2-oxoglutarate (2OG)-and Fe(II)-dependent oxygenase involved in the regulation of IAA and GA (Farrow and Facchini, 2014). IAA regulates cell elongation, cell division, and differentiation, which are important for plant stem development (Jiang et al., 2020; Liu et al., 2021), whereas GA regulates plant nutrient growth and affects plant stem growth (Hedden and Proebsting, 1999). Plant stem shape is largely determined by the shoot apical meristem, whereas branch features are controlled by the axillary meristematic (Hu and Han, 2008). *Glyma.10G220700* and *Glyma.10G224200*, encoded leucine-rich receptor-like protein kinases and ribosomal S13/S18 protein family respectively, and specifically expressed in shoot apical meristem (Lijsebettens et al., 1994; Wang et al., 2010); *Glyma.11G141200* and *Glyma.11G141300* encode a cytosolic isoform of UDP-glucuronic acid decarboxylase, UDP-glucuronic acid decarboxylase produces UDP-xylose, which was the substrate for many cell wall carbohydrates, including hemicellulose and pectin (Zhao et al., 2020); *Glyma.15G143700* encodes beta-xylosidase 1, the homologous Arabidopsis gene *AtBXL1*, was believed to be involved in hemicellulose metabolism of secondary cell wall and plant development (Goujon et al., 2003). These genes are associated with phytohormone regulation (IAA and GA), shoot apical meristem expression, cell proliferation, and secondary cell wall synthesis, which are closely related to plant growth and development, indicating a possible role in stem and branch formation.

### 4.3 Trait-associated markers increased genomic prediction accuracy

Given that SD and BD were significantly correlated (**Table 2**), 93 SD-associated and 84 BD-associated QTLs were used to identify markers for genomic selection model construction. The highest prediction accuracy (0.73) was obtained using BLUE and the mean inferred from different environments for both SD and BD. We noticed that the prediction accuracy of BLUE and mean was higher than that of the different environments. In this study, three marker sets, G1 (all 6098 bin markers), G2 (5841 bin markers not associated with SD or BD), and G3 (257 bin markers associated with SD or BD), were constructed, and the GBLUP model was used for genomic selection. The prediction accuracy of G2 (5841 bin markers unassociated with SD and BD) was 0.37–0.66 for SD and 0.49–0.64 for BD, while the addition of 257 markers associated with the traits to G2 increased the prediction accuracy of SD by 1.52–3.23% and the prediction accuracy of BD by 1.64–4.08%, which is consistent with the phenomenon observed in 100 seed weight, pod length, and pod width (Chen et al., 2023). The GS compensates the shortcomings of traditional marker-assisted selection (MAS), which considers only major-effect other than minor-effect QTLs (Hong et al., 2020), by including all QTLs in the analysis. In addition, the best prediction accuracy was obtained by G3 (257 bin markers associated with SD and BD) in this study, which is consistent with the results of (An et al., 2020) and confirms the concept that trait-associated markers can improve prediction accuracy in many cases (Dang et al., 2023). However, other means referring to optimizing the training population size, the kinship between the training and prediction populations, and different models might also effective in prediction accuracy improvement, which would be elucidated in next study.

## 5 Conclusion

In this study, a RIL population of 363 lines derived from “ZD41×ZYD02878” was used for genetic study of SD and BD in soybean. Combined with the RTM-GWAS, ICIM, and 3VmrMLM methods, 134 SD-associated and 113 BD-associated QTLs were identified; 93 SD-and 84 BD-associated loci were unique, of which 35 loci were mapped by multiple environments or different methods (**Supplementary Table S7**). There were two and ten major QTLs for SD and BD, respectively, with phenotypic variance explained by more than 10%. Candidate gene analysis showed that seven genes involved in the expression of shoot apical meristems, the regulation of phytohormones (IAA and GA), and cell proliferation may be involved in the growth and development of stems and branches. Genomic selection analysis showed that trait-associated markers could significantly enhance prediction accuracy. These results provide a solid foundation for further studies on the SD and BD in soybeans.

## Data availability statement

The data presented in the study are deposited in the Soybean Functional Genomics & Breeding repository, direct access to data through link https://sfgb.rmbreeding.cn/about/data.

## Author contributions

JiW: Validation, Software, Methodology, Data curation, Writing – original draft, Investigation, Formal Analysis. QY: Methodology, Data curation, Writing – review & editing, Investigation, Formal Analysis. YC: Visualization, Software, Writing – review & editing, Investigation, Formal Analysis. KL: Data curation, Writing – review & editing. ZZ: Methodology, Writing – review & editing. YX: Methodology, Writing – review & editing. HY: Data curation, Writing – review & editing. YY: Data curation, Writing – review & editing. JuW: Supervision, Resources, Project administration, Funding acquisition, Writing – original draft, Conceptualization. JS: Validation, Supervision, Resources, Writing – review & editing, Conceptualization. LQ: Supervision, Resources, Project administration, Funding acquisition, Writing – review & editing, Conceptualization.
