# Supplementary material for: QTL mapping and genomic selection of stem and branch diameter in soybean (Glycine max L.)
Source: Front Plant Sci. 2024 May 31;15:1388365. doi: 10.3389/fpls.2024.1388365 (PMC11176531; doi:10.3389/fpls.2024.1388365)
Supplement: Supplementary file 1 [file DataSheet_1.zip › Supplementary Material/Supplementary Material .pdf]

Supplementary Materia

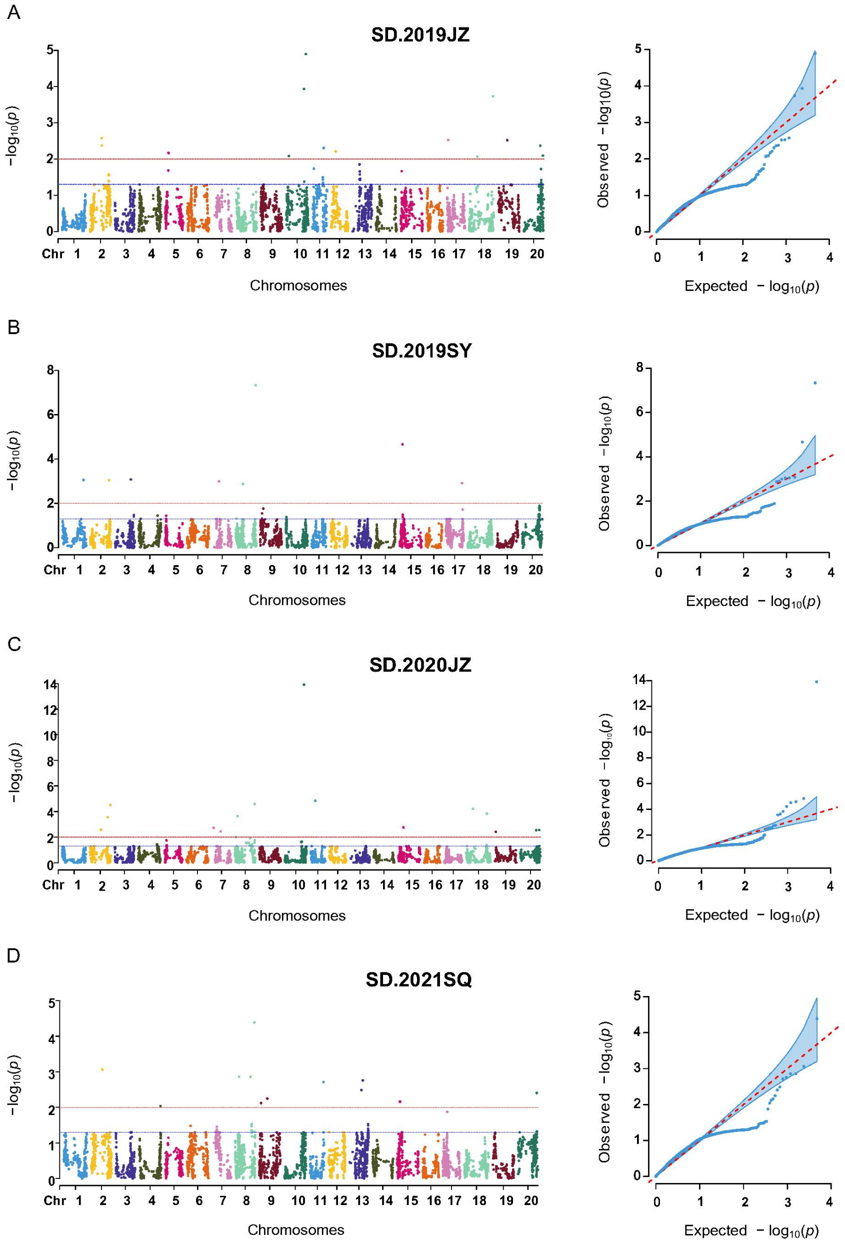

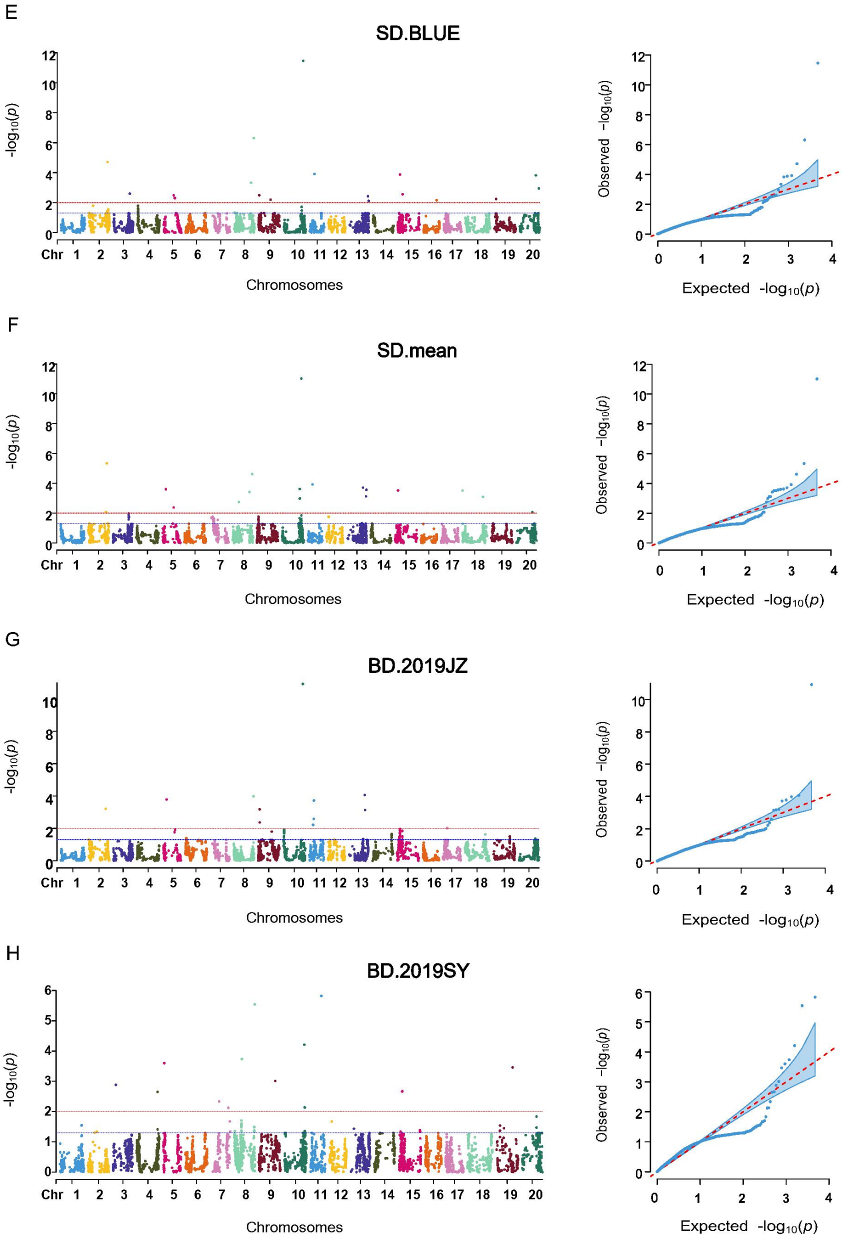
E
SD.BLUE
F
SD.mean
G
BD.2019JZ
H
BD.2019SY
-log10(p)
Chr 1 2 3 4 5 6 7 8 9 10 11 12 13 14 15 16 17 18 19 20
Chromosomes
Observed -log10(p)
Expected -log10(p)

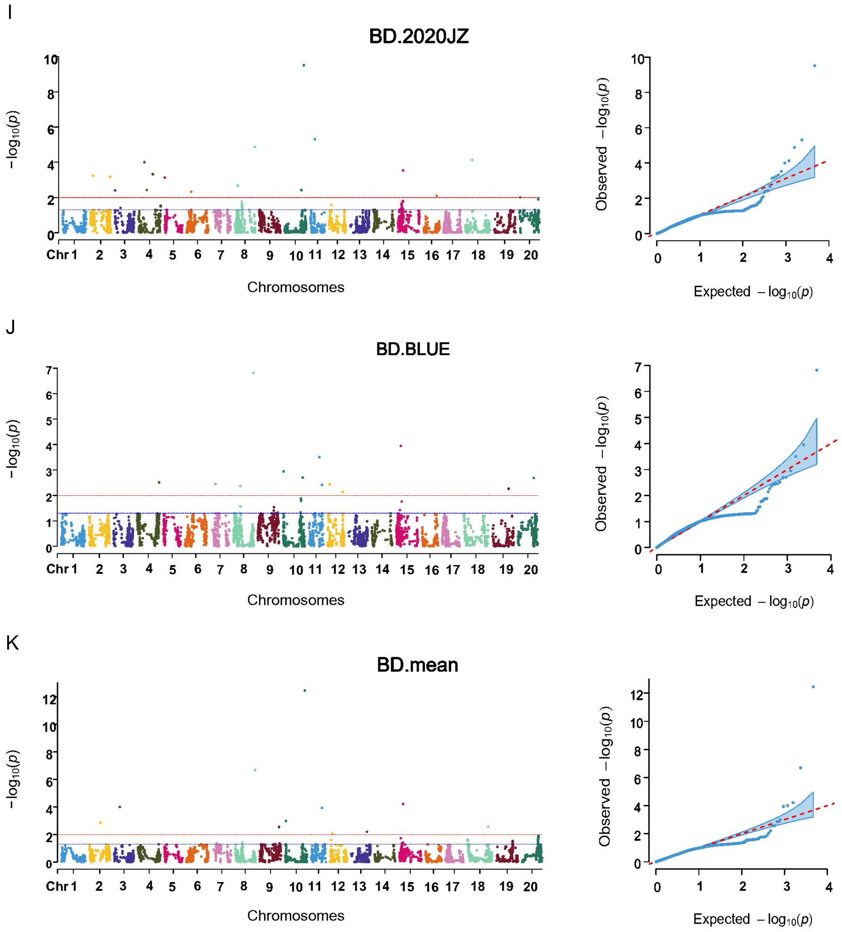

Supplementary Figure S1. Manhattan and QQ plots for SD and BD by RTM-GWAS. Sample panels of Manhattan (left) and quantile–quantile plots (right) for SD (A-F) and BD (G-K) based on different environments, blue and mean. SD: stem diameter; BD: branch diameter; RTM-GWAS, restricted two-stage multi-locus genome-wide association analysis; BLUE, best linear unbiased estimates; JZ: Jingzhou; SY: Sanya; SQ: Shangqiu.

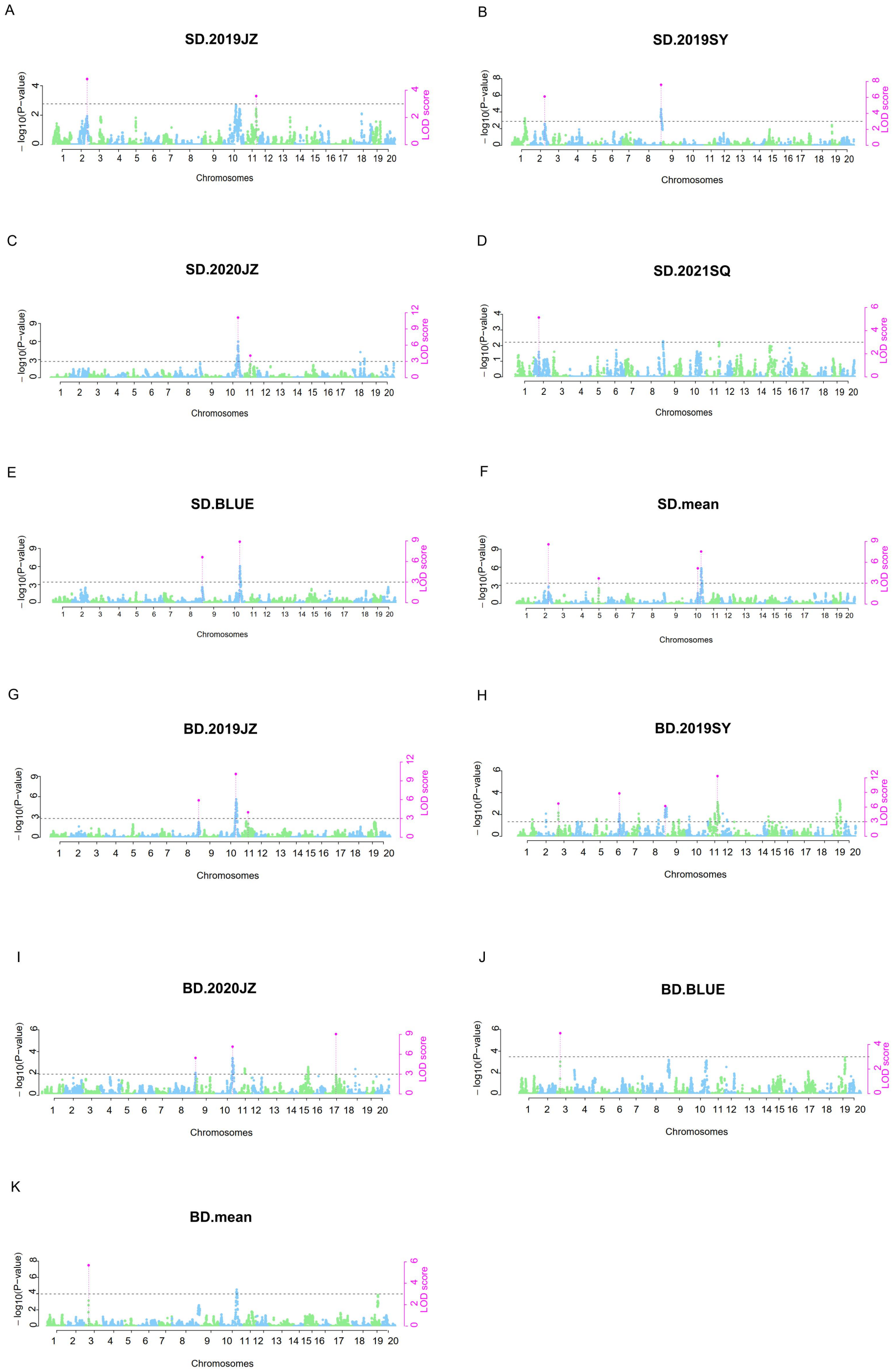

Supplementary Figure S2. Manhattan plots SD and BD by 3VmrMLM. Sample panels of Manhattan plots for SD (A-F) and BD (G-K) based on different environments, blue and mean. 3VmrMLM: three variance component multi locus random SNP effect mixed linear model analysis; LOD, logarithm of odds; SD: stem diameter; BD: branch diameter.

.

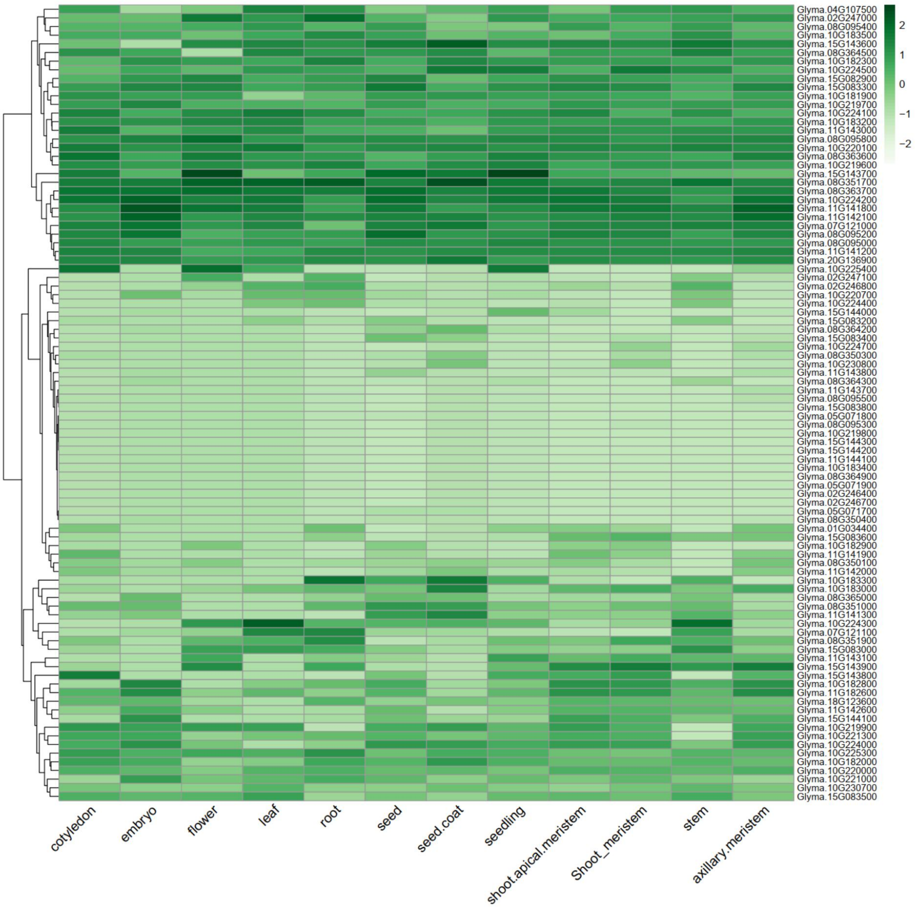

Supplementary Figure S3. Spatial and temporal gene expression pattern of 92 candidate genes. Heat map was constructed using pheatmap package in R. raw data was normalized by log(x+1), where x is gene expression level determined by calculating Fragments Per Kilobase of exon model per Million mapped fragments (FPKM).

Supplementary Table S1. Phenotype of SD and BD in different environments

Line	Year	Location	Replication	SD (mm)	BD (mm)
N222488	2019	JingZhou	1	5.14	2.24
N222489	2019	JingZhou	1	3.39	2.8
N222490	2019	JingZhou	1	5.1	2.36
N222491	2019	JingZhou	1	5.21	3.35
N222492	2019	JingZhou	1	3.11	1.78
N222493	2019	JingZhou	1	2.95	2.26
N222494	2019	JingZhou	1	5.37	3.37
N222495	2019	JingZhou	1	4.86	1.85
N222496	2019	JingZhou	1	5.32	2.28
N222497	2019	JingZhou	1	4.68	2.58
N222498	2019	JingZhou	1	3.78	2.22
N222499	2019	JingZhou	1	3.38	2.73
N222500	2019	JingZhou	1	2.93	1.6
N222501	2019	JingZhou	1	2.3	1.65
N222502	2019	JingZhou	1	4.15	1.95
N222503	2019	JingZhou	1	4.16	2.34
N222504	2019	JingZhou	1	5.8	2.35
N222505	2019	JingZhou	1	6.22	2.98
N222506	2019	JingZhou	1	5.12	2.88
N222507	2019	JingZhou	1	3.8	2.11
N222508	2019	JingZhou	1	4.87	2.91
N222509	2019	JingZhou	1	7.28	2.97
N222510	2019	JingZhou	1	3.24	2.59
N222511	2019	JingZhou	1	5.91	2.54
N222512	2019	JingZhou	1	12.5	3.82
N222513	2019	JingZhou	1	3.23	2.83
N222514	2019	JingZhou	1	5.3	2.74
N222515	2019	JingZhou	1	5.56	3.56
N222516	2019	JingZhou	1	3.45	2.27
N222517	2019	JingZhou	1	6.61	2.82
N222518	2019	JingZhou	1	3.41	2.08
N222519	2019	JingZhou	1	-	-
N222520	2019	JingZhou	1	2.71	2.48
N222521	2019	JingZhou	1	9.6	3.76
N222522	2019	JingZhou	1	4.5	2.59
N222523	2019	JingZhou	1	6.81	3.29
N222524	2019	JingZhou	1	5.6	3.83
N222525	2019	JingZhou	1	4.43	2.98
N222526	2019	JingZhou	1	7.53	2.81
N222527	2019	JingZhou	1	5.14	2.96
N222528	2019	JingZhou	1	4.77	2.32
N222529	2019	JingZhou	1	7.91	3.48
N222530	2019	JingZhou	1	3.74	2.34
N222531	2019	JingZhou	1	3.88	2.25

Line	Year	Location	Replication	SD (mm)	BD (mm)
N222532	2019	JingZhou	1	2.8	1.98
N222533	2019	JingZhou	1	3.17	2.4
N222534	2019	JingZhou	1	4.5	2.54
N222535	2019	JingZhou	1	3.17	2.69
N222536	2019	JingZhou	1	3.8	2.31
N222537	2019	JingZhou	1	3.23	1.68
N222538	2019	JingZhou	1	5.9	3.13
N222539	2019	JingZhou	1	2.59	1.85
N222540	2019	JingZhou	1	4.51	1.62
N222541	2019	JingZhou	1	7.1	2.72
N222542	2019	JingZhou	1	3.45	2.21
N222543	2019	JingZhou	1	6.7	3.03
N222544	2019	JingZhou	1	3.67	2.17
N222545	2019	JingZhou	1	4.64	2.66
N222546	2019	JingZhou	1	5.6	2.92
N222547	2019	JingZhou	1	3.51	2.15
N222548	2019	JingZhou	1	5.6	2.92
N222549	2019	JingZhou	1	4.58	2.67
N222550	2019	JingZhou	1	6.1	3.67
N222551	2019	JingZhou	1	4.83	2.51
N222552	2019	JingZhou	1	5.6	2.71
N222553	2019	JingZhou	1	2.93	2.62
N222554	2019	JingZhou	1	2.27	2.1
N222555	2019	JingZhou	1	2.17	2.01
N222556	2019	JingZhou	1	3.15	2.01
N222557	2019	JingZhou	1	3.5	1.9
N222558	2019	JingZhou	1	3.1	2.24
N222559	2019	JingZhou	1	4.73	2.85
N222560	2019	JingZhou	1	3.99	2.38
N222561	2019	JingZhou	1	4.52	2.18
N222562	2019	JingZhou	1	5.4	2.63
N222563	2019	JingZhou	1	4.43	1.81
N222564	2019	JingZhou	1	3.48	2.08
N222565	2019	JingZhou	1	1.78	1.93
N222566	2019	JingZhou	1	6.8	3.51
N222567	2019	JingZhou	1	6	2.95
N222568	2019	JingZhou	1	5.12	2.51
N222569	2019	JingZhou	1	3.63	2.1
N222570	2019	JingZhou	1	5.91	2.85
N222571	2019	JingZhou	1	5.45	2.06
N222572	2019	JingZhou	1	4.41	3.66
N222573	2019	JingZhou	1	3.27	1.82
N222574	2019	JingZhou	1	9.76	3.81
N222575	2019	JingZhou	1	4.84	2.78
N222576	2019	JingZhou	1	5.48	3.31

Line	Year	Location	Replication	SD (mm)	BD (mm)
N222577	2019	JingZhou	1	4.54	2.39
N222578	2019	JingZhou	1	2.4	2.33
N222579	2019	JingZhou	1	1.32	4.22
N222580	2019	JingZhou	1	1.9	4.19
N222581	2019	JingZhou	1	4.8	3.43
N222582	2019	JingZhou	1	5.29	3.7
N222583	2019	JingZhou	1	2.78	2.18
N222584	2019	JingZhou	1	5.75	2.87
N222585	2019	JingZhou	1	7.88	2.96
N222586	2019	JingZhou	1	4.24	2.67
N222587	2019	JingZhou	1	3.92	1.99
N222588	2019	JingZhou	1	2.66	3.17
N222589	2019	JingZhou	1	4.1	2.22
N222590	2019	JingZhou	1	4.47	2.7
N222591	2019	JingZhou	1	3.55	2
N222592	2019	JingZhou	1	5.46	2.95
N222593	2019	JingZhou	1	5.13	2.54
N222594	2019	JingZhou	1	3.9	2.95
N222595	2019	JingZhou	1	3.59	2.32
N222596	2019	JingZhou	1	2.82	1.79
N222597	2019	JingZhou	1	7.89	4.6
N222598	2019	JingZhou	1	3.4	1.75
N222599	2019	JingZhou	1	6.61	2.75
N222600	2019	JingZhou	1	3.1	2.07
N222601	2019	JingZhou	1	4.72	2.08
N222602	2019	JingZhou	1	5.12	2.58
N222603	2019	JingZhou	1	7.9	2.87
N222604	2019	JingZhou	1	3.61	2.55
N222605	2019	JingZhou	1	4.65	2.44
N222606	2019	JingZhou	1	2.48	2.43
N222607	2019	JingZhou	1	6.15	2.64
N222608	2019	JingZhou	1	3.2	1.88
N222609	2019	JingZhou	1	2.77	1.49
N222610	2019	JingZhou	1	4.13	2.2
N222611	2019	JingZhou	1	3.8	2.62
N222612	2019	JingZhou	1	2.62	2.75
N222613	2019	JingZhou	1	5.25	2.23
N222614	2019	JingZhou	1	3.36	2.32
N222615	2019	JingZhou	1	-	-
N222616	2019	JingZhou	1	5.56	3.41
N222617	2019	JingZhou	1	3.93	2.18
N222618	2019	JingZhou	1	5.31	2.15
N222619	2019	JingZhou	1	2.32	1.4
N222620	2019	JingZhou	1	1.87	1.45
N222621	2019	JingZhou	1	2.9	1.09

Line	Year	Location	Replication	SD (mm)	BD (mm)
N222622	2019	JingZhou	1	4.45	2.08
N222623	2019	JingZhou	1	2.96	1.56
N222624	2019	JingZhou	1	4.9	2.4
N222625	2019	JingZhou	1	3.7	2.52
N222628	2019	JingZhou	1	5.15	3.37
N222629	2019	JingZhou	1	4.8	1.97
N222630	2019	JingZhou	1	4.52	2.98
N222631	2019	JingZhou	1	7.41	2.58
N222632	2019	JingZhou	1	6.84	3.74
N222633	2019	JingZhou	1	5.2	2.78
N222634	2019	JingZhou	1	5.41	3.07
N222635	2019	JingZhou	1	2.62	1.71
N222636	2019	JingZhou	1	3	1.88
N222637	2019	JingZhou	1	3.86	2.59
N222638	2019	JingZhou	1	3.88	2.45
N222639	2019	JingZhou	1	6.82	2.7
N222640	2019	JingZhou	1	5.29	2.48
N222641	2019	JingZhou	1	4.35	2.43
N222642	2019	JingZhou	1	6.1	3.23
N222643	2019	JingZhou	1	6.29	2.73
N222644	2019	JingZhou	1	4.27	2.69
N222645	2019	JingZhou	1	4.2	2.38
N222646	2019	JingZhou	1	5.92	2.81
N222647	2019	JingZhou	1	7.85	2.03
N222648	2019	JingZhou	1	4.82	2.21
N222649	2019	JingZhou	1	4.44	2.96
N222650	2019	JingZhou	1	8.21	3.26
N222651	2019	JingZhou	1	4.56	3.22
N222652	2019	JingZhou	1	3.72	2.2
N222653	2019	JingZhou	1	2.33	1.65
N222654	2019	JingZhou	1	4.78	2.48
N222655	2019	JingZhou	1	4.76	2.68
N222656	2019	JingZhou	1	3.27	2.08
N222657	2019	JingZhou	1	6.74	3.07
N222658	2019	JingZhou	1	5.48	4.36
N222659	2019	JingZhou	1	7.7	3.19
N222660	2019	JingZhou	1	6.53	3.19
N222661	2019	JingZhou	1	7.5	3.65
N222662	2019	JingZhou	1	5.17	3.89
N222663	2019	JingZhou	1	6.7	2.2
N222664	2019	JingZhou	1	2.82	1.55
N222665	2019	JingZhou	1	5.72	2.31
N222666	2019	JingZhou	1	4.29	2.84
N222667	2019	JingZhou	1	4.3	2.88
N222668	2019	JingZhou	1	3.34	2.13

Line	Year	Location	Replication	SD (mm)	BD (mm)
N222669	2019	JingZhou	1	3.45	1.95
N222670	2019	JingZhou	1	4.36	2.43
N222671	2019	JingZhou	1	5.18	2.89
N222672	2019	JingZhou	1	2.46	2.27
N222673	2019	JingZhou	1	3.47	2.19
N222674	2019	JingZhou	1	5.68	3.32
N222675	2019	JingZhou	1	4.94	2.64
N222676	2019	JingZhou	1	7.78	2.52
N222677	2019	JingZhou	1	4.75	2.32
N222678	2019	JingZhou	1	4.9	1.88
N222679	2019	JingZhou	1	4.58	2.59
N222680	2019	JingZhou	1	5.48	2.26
N222681	2019	JingZhou	1	5.63	4.35
N222682	2019	JingZhou	1	9.1	3.7
N222683	2019	JingZhou	1	6.67	3.3
N222684	2019	JingZhou	1	5	2.55
N222685	2019	JingZhou	1	4.29	2.41
N222686	2019	JingZhou	1	4.45	3.19
N222687	2019	JingZhou	1	3.9	2.42
N222688	2019	JingZhou	1	3.15	1.92
N222689	2019	JingZhou	1	3.71	2.34
N222690	2019	JingZhou	1	5.96	2.84
N222691	2019	JingZhou	1	3.8	2.67
N222692	2019	JingZhou	1	5.2	2.76
N222693	2019	JingZhou	1	3.38	2.06
N222694	2019	JingZhou	1	4.6	2.14
N222695	2019	JingZhou	1	4.22	2.44
N222696	2019	JingZhou	1	4.13	2.04
N222697	2019	JingZhou	1	4.48	2.46
N222698	2019	JingZhou	1	3.21	2.53
N222699	2019	JingZhou	1	4.2	2.43
N222700	2019	JingZhou	1	5.84	3.33
N222701	2019	JingZhou	1	8.76	3.54
N222702	2019	JingZhou	1	6.4	3.4
N222703	2019	JingZhou	1	5.72	2.76
N222704	2019	JingZhou	1	7.27	2.65
N222705	2019	JingZhou	1	5.61	2.51
N222706	2019	JingZhou	1	6.4	3.13
N222707	2019	JingZhou	1	5.17	2.7
N222708	2019	JingZhou	1	4.19	2.08
N222709	2019	JingZhou	1	3.78	2.95
N222710	2019	JingZhou	1	4.1	2.47
N222711	2019	JingZhou	1	6.81	2.64
N222712	2019	JingZhou	1	5.51	3.03
N222713	2019	JingZhou	1	5.52	1.77

Line	Year	Location	Replication	SD (mm)	BD (mm)
N222715	2019	JingZhou	1	4.3	3.17
N222716	2019	JingZhou	1	8.94	3.51
N222717	2019	JingZhou	1	12.13	5.23
N222718	2019	JingZhou	1	8.69	3.57
N222719	2019	JingZhou	1	7.15	3.3
N222720	2019	JingZhou	1	7.3	3.42
N222721	2019	JingZhou	1	4.13	2
N222722	2019	JingZhou	1	5.85	3.02
N222723	2019	JingZhou	1	4.97	2.18
N222724	2019	JingZhou	1	6.21	2.22
N222725	2019	JingZhou	1	3.65	2.18
N222726	2019	JingZhou	1	3.24	2.17
N222727	2019	JingZhou	1	2.76	1.57
N222728	2019	JingZhou	1	3.6	3.17
N222729	2019	JingZhou	1	4.59	3.54
N222730	2019	JingZhou	1	3.58	2.28
N222731	2019	JingZhou	1	2.91	1.86
N222732	2019	JingZhou	1	3.2	2.13
N222733	2019	JingZhou	1	3.72	2.17
N222734	2019	JingZhou	1	8.7	4.97
N222735	2019	JingZhou	1	2.45	2.51
N222736	2019	JingZhou	1	3.43	2.35
N222737	2019	JingZhou	1	4.6	2.72
N222738	2019	JingZhou	1	3.21	1.89
N222739	2019	JingZhou	1	2.5	1.32
N222740	2019	JingZhou	1	3.19	2.7
N222741	2019	JingZhou	1	3.95	2.99
N222742	2019	JingZhou	1	1.33	1.18
N222743	2019	JingZhou	1	3.89	2.39
N222744	2019	JingZhou	1	9.95	3.48
N222745	2019	JingZhou	1	3.99	2.24
N222746	2019	JingZhou	1	-	-
N222747	2019	JingZhou	1	2.92	2.21
N222748	2019	JingZhou	1	3.9	2.04
N222749	2019	JingZhou	1	3.8	1.16
N222750	2019	JingZhou	1	1.68	1.89
N222751	2019	JingZhou	1	2.55	1.83
N222752	2019	JingZhou	1	3.18	2.29
N222753	2019	JingZhou	1	7.8	2.98
N222754	2019	JingZhou	1	4.5	2.24
N222755	2019	JingZhou	1	4.77	2.34
N222756	2019	JingZhou	1	5.53	2.47
N222757	2019	JingZhou	1	6.87	3.36
N222759	2019	JingZhou	1	3.1	1.92
N222760	2019	JingZhou	1	3.93	1.95

Line	Year	Location	Replication	SD (mm)	BD (mm)
N222761	2019	JingZhou	1	3.51	2.56
N222762	2019	JingZhou	1	7.84	3.04
N222763	2019	JingZhou	1	2.42	1.87
N222764	2019	JingZhou	1	3.98	2.52
N222765	2019	JingZhou	1	2.88	1.63
N222766	2019	JingZhou	1	4.38	1.84
N222767	2019	JingZhou	1	5.2	2.1
N222768	2019	JingZhou	1	3.4	2.18
N222769	2019	JingZhou	1	3.43	2.1
N222770	2019	JingZhou	1	2.24	1.69
N222771	2019	JingZhou	1	3.79	3.07
N222772	2019	JingZhou	1	3.74	2.25
N222773	2019	JingZhou	1	3.62	2.59
N222774	2019	JingZhou	1	4.91	2.69
N222775	2019	JingZhou	1	1.92	1.35
N222776	2019	JingZhou	1	2.2	1.88
N222777	2019	JingZhou	1	3.49	1.5
N222778	2019	JingZhou	1	2.17	1.72
N222779	2019	JingZhou	1	-	2.4
N222780	2019	JingZhou	1	-	2.58
N222781	2019	JingZhou	1	-	2.61
N222782	2019	JingZhou	1	-	2.84
N222783	2019	JingZhou	1	2.9	1.81
N222784	2019	JingZhou	1	4.19	2.49
N222785	2019	JingZhou	1	2.19	1.87
N222786	2019	JingZhou	1	5.19	2.58
N222787	2019	JingZhou	1	3.97	2
N222788	2019	JingZhou	1	4.65	2.29
N222789	2019	JingZhou	1	4.2	2.88
N222790	2019	JingZhou	1	5.2	2.94
N222791	2019	JingZhou	1	3.26	2.55
N222792	2019	JingZhou	1	3.96	2.48
N222793	2019	JingZhou	1	3.43	2.41
N222794	2019	JingZhou	1	3.42	2.06
N222795	2019	JingZhou	1	5.47	3.76
N222796	2019	JingZhou	1	3.45	1.73
N222797	2019	JingZhou	1	5.12	2.1
N222798	2019	JingZhou	1	4.7	3.01
N222799	2019	JingZhou	1	6.39	2.81
N222800	2019	JingZhou	1	6.5	2.67
N222801	2019	JingZhou	1	8.57	3.71
N222802	2019	JingZhou	1	4	2.68
N222803	2019	JingZhou	1	3.44	1.94
N222804	2019	JingZhou	1	4.38	1.74
N222805	2019	JingZhou	1	5.3	2.34

Line	Year	Location	Replication	SD (mm)	BD (mm)
N222806	2019	JingZhou	1	3.89	2.79
N222807	2019	JingZhou	1	4.88	2.16
N222808	2019	JingZhou	1	8.81	3.58
N222809	2019	JingZhou	1	3.16	1.87
N222810	2019	JingZhou	1	6.35	2.63
N222811	2019	JingZhou	1	3.37	1.85
N222812	2019	JingZhou	1	3.85	2.29
N222813	2019	JingZhou	1	5.26	2.3
N222814	2019	JingZhou	1	3.75	2.66
N222815	2019	JingZhou	1	5.61	2.46
N222816	2019	JingZhou	1	3.1	2.23
N222817	2019	JingZhou	1	3.56	2.91
N222818	2019	JingZhou	1	5.7	3.35
N222819	2019	JingZhou	1	6.5	2.58
N222820	2019	JingZhou	1	2.75	2.63
N222821	2019	JingZhou	1	3.18	2.01
N222822	2019	JingZhou	1	2.95	2.12
N222823	2019	JingZhou	1	4.28	2.45
N222824	2019	JingZhou	1	2.57	1.7
N222825	2019	JingZhou	1	4.24	2.45
N222826	2019	JingZhou	1	3.49	2.53
N222827	2019	JingZhou	1	4.16	2.42
N222828	2019	JingZhou	1	6.39	3.25
N222829	2019	JingZhou	1	5.1	2.44
N222830	2019	JingZhou	1	3.89	2.72
N222831	2019	JingZhou	1	4.75	2.2
N222832	2019	JingZhou	1	6.6	5.1
N222833	2019	JingZhou	1	6.59	3.32
N222834	2019	JingZhou	1	4.76	2.4
N222835	2019	JingZhou	1	3.76	3.29
N222836	2019	JingZhou	1	4.7	2.38
N222837	2019	JingZhou	1	3.67	3.9
N222838	2019	JingZhou	1	4.71	2.87
N222839	2019	JingZhou	1	4.75	2.38
N222840	2019	JingZhou	1	5.6	3.24
N222841	2019	JingZhou	1	4.5	2.02
N222842	2019	JingZhou	1	6.73	4.38
N222843	2019	JingZhou	1	4.59	2.38
N222844	2019	JingZhou	1	5.52	2.52
N222845	2019	JingZhou	1	3.2	1.89
N222846	2019	JingZhou	1	9.36	3.6
N222847	2019	JingZhou	1	2.45	1.66
N222848	2019	JingZhou	1	6.21	3.94
N222849	2019	JingZhou	1	3.85	2.43
N222850	2019	JingZhou	1	2.72	1.9

Line	Year	Location	Replication	SD (mm)	BD (mm)
N222851	2019	JingZhou	1	1.48	1.32
N222852	2019	JingZhou	1	3.6	2.14
N222853	2019	JingZhou	1	3.19	1.3
N222854	2019	JingZhou	1	4.6	2.3
ZD41	2019	JingZhou	1	6.45	3.77
ZYD02878	2019	JingZhou	1	0.82	0.9
N222488	2019	Sanya	1	1.94	2.82
N222488	2019	Sanya	2	2.45	2.15
N222488	2019	Sanya	3	3.13	2.11
N222489	2019	Sanya	1	1.44	1.39
N222489	2019	Sanya	2	2.49	2.19
N222489	2019	Sanya	3	1.81	1.96
N222490	2019	Sanya	1	1.96	1.32
N222490	2019	Sanya	2	1.8	1.5
N222490	2019	Sanya	3	1.74	1.65
N222491	2019	Sanya	1	3.96	2.34
N222491	2019	Sanya	2	3.97	1.99
N222491	2019	Sanya	3	3.57	2.25
N222492	2019	Sanya	1	2.52	1.98
N222492	2019	Sanya	2	2.15	2.56
N222492	2019	Sanya	3	2.07	1.88
N222493	2019	Sanya	1	2.35	1.89
N222493	2019	Sanya	2	2.72	1.99
N222493	2019	Sanya	3	2.69	1.72
N222494	2019	Sanya	1	2.38	2.08
N222494	2019	Sanya	2	3.13	1.85
N222494	2019	Sanya	3	3.29	2.51
N222495	2019	Sanya	1	2.38	1.9
N222495	2019	Sanya	2	2.25	1.33
N222495	2019	Sanya	3	1.66	1.17
N222496	2019	Sanya	1	3.28	2.3
N222496	2019	Sanya	2	3.11	2.37
N222496	2019	Sanya	3	3.96	2.58
N222497	2019	Sanya	1	2.53	2.1
N222497	2019	Sanya	2	3.48	2.3
N222497	2019	Sanya	3	2.51	1.69
N222498	2019	Sanya	1	2.27	1.43
N222498	2019	Sanya	2	4.26	2.76
N222498	2019	Sanya	3	3.85	2.45
N222499	2019	Sanya	1	1.83	1.43
N222499	2019	Sanya	2	1.96	1.96
N222499	2019	Sanya	3	1.8	1.33
N222500	2019	Sanya	1	2.1	1.12
N222500	2019	Sanya	2	2.18	1.17
N222500	2019	Sanya	3	2.25	1.54

Line	Year	Location	Replication	SD (mm)	BD (mm)
N222501	2019	Sanya	1	2.13	2.14
N222501	2019	Sanya	2	2.39	2.66
N222501	2019	Sanya	3	2.01	1.9
N222502	2019	Sanya	1	2.49	2.17
N222502	2019	Sanya	2	1.94	1.89
N222502	2019	Sanya	3	2.08	1.65
N222503	2019	Sanya	1	2.25	1.85
N222503	2019	Sanya	2	2.06	2.42
N222503	2019	Sanya	3	2.61	2.01
N222504	2019	Sanya	1	2.62	2.19
N222504	2019	Sanya	2	1.78	2.01
N222504	2019	Sanya	3	2.7	2.04
N222505	2019	Sanya	1	2.68	2.54
N222505	2019	Sanya	2	3.44	3.15
N222505	2019	Sanya	3	1.92	2.97
N222506	2019	Sanya	1	2.17	1.12
N222506	2019	Sanya	2	3.66	1.79
N222506	2019	Sanya	3	3.01	1.87
N222507	2019	Sanya	1	2.43	1.7
N222507	2019	Sanya	2	3.42	2.71
N222507	2019	Sanya	3	2.23	2.4
N222508	2019	Sanya	1	2.58	1.93
N222508	2019	Sanya	2	2.35	2.21
N222508	2019	Sanya	3	2.84	2.24
N222509	2019	Sanya	1	1.76	2.39
N222509	2019	Sanya	2	1.86	2.44
N222509	2019	Sanya	3	2.18	2.36
N222510	2019	Sanya	1	1.95	1.66
N222510	2019	Sanya	2	2.08	1.93
N222510	2019	Sanya	3	1.53	1.88
N222511	2019	Sanya	1	3.29	2.03
N222511	2019	Sanya	2	3.86	2.32
N222511	2019	Sanya	3	2.88	2.24
N222512	2019	Sanya	1	2.62	2.47
N222512	2019	Sanya	2	2.09	1.97
N222512	2019	Sanya	3	2.12	2.23
N222513	2019	Sanya	1	1.49	1.55
N222513	2019	Sanya	2	2.02	1.95
N222513	2019	Sanya	3	1.57	1.33
N222514	2019	Sanya	1	2.84	2.55
N222514	2019	Sanya	2	3.69	2.49
N222514	2019	Sanya	3	2.02	2.88
N222515	2019	Sanya	1	2.16	2.46
N222515	2019	Sanya	2	1.46	2.16
N222515	2019	Sanya	3	1.77	1.49

Line	Year	Location	Replication	SD (mm)	BD (mm)
N222516	2019	Sanya	1	1.87	2.01
N222516	2019	Sanya	2	2.16	1.68
N222516	2019	Sanya	3	1.6	0.61
N222517	2019	Sanya	1	2.72	2.62
N222517	2019	Sanya	2	3.12	2.03
N222517	2019	Sanya	3	2.57	2.26
N222518	2019	Sanya	1	1.92	1.64
N222518	2019	Sanya	2	2.82	2.1
N222518	2019	Sanya	3	2.46	1.87
N222519	2019	Sanya	1	2.03	1.57
N222519	2019	Sanya	2	1.8	1.4
N222519	2019	Sanya	3	1.79	1.68
N222520	2019	Sanya	1	2.47	2.11
N222520	2019	Sanya	2	1.97	2.13
N222520	2019	Sanya	3	2.13	2.26
N222521	2019	Sanya	1	2.54	1.81
N222521	2019	Sanya	2	2.7	2.46
N222521	2019	Sanya	3	2.38	2
N222522	2019	Sanya	1	3.11	1.91
N222522	2019	Sanya	2	2.06	2.45
N222522	2019	Sanya	3	2.18	2.12
N222523	2019	Sanya	1	2.18	3.03
N222523	2019	Sanya	2	2.91	2.32
N222523	2019	Sanya	3	-	-
N222524	2019	Sanya	1	1.56	1.96
N222524	2019	Sanya	2	2.24	2.02
N222524	2019	Sanya	3	1.67	2.04
N222525	2019	Sanya	1	2.31	2.61
N222525	2019	Sanya	2	2.41	2.14
N222525	2019	Sanya	3	2.68	2.35
N222526	2019	Sanya	1	2.86	1.86
N222526	2019	Sanya	2	3.3	2.64
N222526	2019	Sanya	3	3.54	2.03
N222527	2019	Sanya	1	2.18	2.24
N222527	2019	Sanya	2	2.64	2.07
N222527	2019	Sanya	3	2.77	2.44
N222528	2019	Sanya	1	2.25	1.81
N222528	2019	Sanya	2	2.46	2.18
N222528	2019	Sanya	3	2.72	2.08
N222529	2019	Sanya	1	2.26	2.67
N222529	2019	Sanya	2	2.48	2.97
N222529	2019	Sanya	3	2.54	2.31
N222530	2019	Sanya	1	2.64	2.01
N222530	2019	Sanya	2	2.27	2.79
N222530	2019	Sanya	3	2.69	1.95

Line	Year	Location	Replication	SD (mm)	BD (mm)
N222531	2019	Sanya	1	3.44	2.6
N222531	2019	Sanya	2	2.95	1.97
N222531	2019	Sanya	3	2.91	2.87
N222532	2019	Sanya	1	1.44	1.46
N222532	2019	Sanya	2	2.23	1.33
N222532	2019	Sanya	3	1.9	0.93
N222533	2019	Sanya	1	2	1.2
N222533	2019	Sanya	2	1.51	1.27
N222533	2019	Sanya	3	1.76	1.78
N222534	2019	Sanya	1	2	-
N222534	2019	Sanya	2	2.31	-
N222534	2019	Sanya	3	1.72	-
N222535	2019	Sanya	1	2.16	1.93
N222535	2019	Sanya	2	2.07	2.06
N222535	2019	Sanya	3	1.78	1.98
N222536	2019	Sanya	1	1.67	1.44
N222536	2019	Sanya	2	3.67	1.85
N222536	2019	Sanya	3	2.72	1.59
N222537	2019	Sanya	1	2.75	2.76
N222537	2019	Sanya	2	2.13	2.24
N222537	2019	Sanya	3	1.38	2.18
N222538	2019	Sanya	1	3.36	1.54
N222538	2019	Sanya	2	2.7	1.52
N222538	2019	Sanya	3	3.56	1.59
N222539	2019	Sanya	1	2.17	1.86
N222539	2019	Sanya	2	2.22	2.02
N222539	2019	Sanya	3	2.23	1.87
N222540	2019	Sanya	1	2.15	2.36
N222540	2019	Sanya	2	1.84	1.74
N222540	2019	Sanya	3	2.13	1.92
N222541	2019	Sanya	1	2.71	-
N222541	2019	Sanya	2	3.15	1.79
N222541	2019	Sanya	3	3.48	2.4
N222542	2019	Sanya	1	2.45	1.87
N222542	2019	Sanya	2	1.96	1.26
N222542	2019	Sanya	3	2.02	1.64
N222543	2019	Sanya	1	2.17	1.92
N222543	2019	Sanya	2	2	1.92
N222543	2019	Sanya	3	2.47	2.01
N222544	2019	Sanya	1	2.2	1.77
N222544	2019	Sanya	2	2.79	2.24
N222544	2019	Sanya	3	2.67	1.77
N222545	2019	Sanya	1	2.02	1.97
N222545	2019	Sanya	2	1.46	2.14
N222545	2019	Sanya	3	1.9	1.74

Line	Year	Location	Replication	SD (mm)	BD (mm)
N222546	2019	Sanya	1	1.41	1.23
N222546	2019	Sanya	2	1.99	1.28
N222546	2019	Sanya	3	1.86	1.91
N222547	2019	Sanya	1	1.89	1.57
N222547	2019	Sanya	2	1.96	1.81
N222547	2019	Sanya	3	1.98	1.67
N222548	2019	Sanya	1	2.16	1.53
N222548	2019	Sanya	2	2.6	1.89
N222548	2019	Sanya	3	2.23	1.5
N222549	2019	Sanya	1	2.57	1.47
N222549	2019	Sanya	2	2.23	1.36
N222549	2019	Sanya	3	3.5	1.58
N222550	2019	Sanya	1	3.04	2.54
N222550	2019	Sanya	2	1.66	2.65
N222550	2019	Sanya	3	3.4	2.52
N222551	2019	Sanya	1	3.01	2.19
N222551	2019	Sanya	2	2.68	1.81
N222551	2019	Sanya	3	2.98	2.21
N222552	2019	Sanya	1	2.67	1.63
N222552	2019	Sanya	2	2.18	1.69
N222552	2019	Sanya	3	2.36	1.66
N222553	2019	Sanya	1	3.91	2.3
N222553	2019	Sanya	2	-	-
N222553	2019	Sanya	3	3.73	2.72
N222554	2019	Sanya	1	1.6	1.79
N222554	2019	Sanya	2	1.68	1.65
N222554	2019	Sanya	3	1.65	2.23
N222555	2019	Sanya	1	2.07	2.04
N222555	2019	Sanya	2	2.36	2.1
N222555	2019	Sanya	3	2.12	1.99
N222556	2019	Sanya	1	2.09	1.69
N222556	2019	Sanya	2	2.01	1.7
N222556	2019	Sanya	3	1.7	1.63
N222557	2019	Sanya	1	2.31	1.45
N222557	2019	Sanya	2	2.95	2.02
N222557	2019	Sanya	3	2.45	1.23
N222558	2019	Sanya	1	1.91	2.24
N222558	2019	Sanya	2	2.5	2
N222558	2019	Sanya	3	2.76	2.2
N222559	2019	Sanya	1	2.2	1.68
N222559	2019	Sanya	2	2.35	1.3
N222559	2019	Sanya	3	2.62	1.47
N222560	2019	Sanya	1	2.92	1.54
N222560	2019	Sanya	2	2.97	1.61
N222560	2019	Sanya	3	3.05	1.82

Line	Year	Location	Replication	SD (mm)	BD (mm)
N222561	2019	Sanya	1	2.26	1.91
N222561	2019	Sanya	2	2.84	1.97
N222561	2019	Sanya	3	2.16	2.13
N222562	2019	Sanya	1	2.88	1.68
N222562	2019	Sanya	2	2.34	1.45
N222562	2019	Sanya	3	2.64	1.24
N222563	2019	Sanya	1	2.42	1.48
N222563	2019	Sanya	2	1.92	1.28
N222563	2019	Sanya	3	1.92	-
N222564	2019	Sanya	1	2	1.52
N222564	2019	Sanya	2	1.73	1.29
N222564	2019	Sanya	3	2.5	1.77
N222565	2019	Sanya	1	2.23	2.7
N222565	2019	Sanya	2	3.52	2.04
N222565	2019	Sanya	3	4.4	2.62
N222566	2019	Sanya	1	3.53	2.43
N222566	2019	Sanya	2	3.29	2.17
N222566	2019	Sanya	3	3.89	2.33
N222567	2019	Sanya	1	3.2	2.05
N222567	2019	Sanya	2	2.98	2.09
N222567	2019	Sanya	3	3.94	2.03
N222568	2019	Sanya	1	2.88	1.86
N222568	2019	Sanya	2	2.03	1.49
N222568	2019	Sanya	3	2.3	1.88
N222569	2019	Sanya	1	2.38	1.87
N222569	2019	Sanya	2	2.27	1.88
N222569	2019	Sanya	3	2.33	1.95
N222570	2019	Sanya	1	3.38	2.15
N222570	2019	Sanya	2	2.79	2.3
N222570	2019	Sanya	3	3.46	2.7
N222571	2019	Sanya	1	2.02	1.59
N222571	2019	Sanya	2	2.2	1.62
N222571	2019	Sanya	3	1.7	1.38
N222572	2019	Sanya	1	2.31	1.75
N222572	2019	Sanya	2	1.93	1.95
N222572	2019	Sanya	3	2.11	1.86
N222573	2019	Sanya	1	2.34	1.72
N222573	2019	Sanya	2	2.19	1.82
N222573	2019	Sanya	3	2.53	1.49
N222574	2019	Sanya	1	3.86	2.22
N222574	2019	Sanya	2	3.87	2.23
N222574	2019	Sanya	3	3.36	1.77
N222575	2019	Sanya	1	2.86	2.33
N222575	2019	Sanya	2	2.71	2.16
N222575	2019	Sanya	3	2.9	2.17

Line	Year	Location	Replication	SD (mm)	BD (mm)
N222576	2019	Sanya	1	2.88	2.31
N222576	2019	Sanya	2	1.52	2.17
N222576	2019	Sanya	3	1.94	1.67
N222577	2019	Sanya	1	3.08	2.27
N222577	2019	Sanya	2	3.11	2.02
N222577	2019	Sanya	3	-	-
N222578	2019	Sanya	1	1.8	1.31
N222578	2019	Sanya	2	1.77	1.59
N222578	2019	Sanya	3	1.96	1.52
N222579	2019	Sanya	1	4.3	3.1
N222579	2019	Sanya	2	4	2.85
N222579	2019	Sanya	3	4.04	3.34
N222580	2019	Sanya	1	3.46	2.8
N222580	2019	Sanya	2	3.97	3.24
N222580	2019	Sanya	3	5.1	3.17
N222581	2019	Sanya	1	2.58	1.87
N222581	2019	Sanya	2	2.27	2.29
N222581	2019	Sanya	3	3.53	2.21
N222582	2019	Sanya	1	1.57	1.47
N222582	2019	Sanya	2	2.94	2.24
N222582	2019	Sanya	3	2.64	2.19
N222583	2019	Sanya	1	2.35	1.9
N222583	2019	Sanya	2	2.09	1.66
N222583	2019	Sanya	3	1.99	1.53
N222584	2019	Sanya	1	3.28	2.04
N222584	2019	Sanya	2	3.7	2.56
N222584	2019	Sanya	3	3.29	2.48
N222585	2019	Sanya	1	3.56	1.95
N222585	2019	Sanya	2	2.77	1.63
N222585	2019	Sanya	3	3.07	1.9
N222586	2019	Sanya	1	2.7	2.21
N222586	2019	Sanya	2	2.57	1.96
N222586	2019	Sanya	3	2.26	1.76
N222587	2019	Sanya	1	4.68	2.87
N222587	2019	Sanya	2	3.72	2.19
N222587	2019	Sanya	3	-	-
N222588	2019	Sanya	1	2.16	1.75
N222588	2019	Sanya	2	2.49	2.17
N222588	2019	Sanya	3	1.9	1.75
N222589	2019	Sanya	1	1.46	0.87
N222589	2019	Sanya	2	2.32	1.43
N222589	2019	Sanya	3	1.8	1.45
N222590	2019	Sanya	1	1.98	1.23
N222590	2019	Sanya	2	2.08	1.23
N222590	2019	Sanya	3	2.05	1.44

Line	Year	Location	Replication	SD (mm)	BD (mm)
N222591	2019	Sanya	1	1.39	1.83
N222591	2019	Sanya	2	1.45	1.18
N222591	2019	Sanya	3	2.81	1.9
N222592	2019	Sanya	1	2.46	1.23
N222592	2019	Sanya	2	2.19	1.33
N222592	2019	Sanya	3	2.34	1.51
N222593	2019	Sanya	1	2.17	1.64
N222593	2019	Sanya	2	2.62	1.61
N222593	2019	Sanya	3	2.22	1.14
N222594	2019	Sanya	1	2.25	1.67
N222594	2019	Sanya	2	1.83	1.9
N222594	2019	Sanya	3	1.95	1.45
N222595	2019	Sanya	1	2.75	1.49
N222595	2019	Sanya	2	2.54	1.47
N222595	2019	Sanya	3	2.59	1.48
N222596	2019	Sanya	1	1.96	1.42
N222596	2019	Sanya	2	1.3	1.06
N222596	2019	Sanya	3	1.41	1.42
N222597	2019	Sanya	1	3.02	2.02
N222597	2019	Sanya	2	2.13	1.63
N222597	2019	Sanya	3	1.88	1.26
N222598	2019	Sanya	1	3.39	-
N222598	2019	Sanya	2	1.53	1.86
N222598	2019	Sanya	3	1.24	-
N222599	2019	Sanya	1	3.49	1.79
N222599	2019	Sanya	2	2.92	1.95
N222599	2019	Sanya	3	1.83	1.8
N222600	2019	Sanya	1	2.76	1.69
N222600	2019	Sanya	2	2.18	1.67
N222600	2019	Sanya	3	2.03	1.84
N222601	2019	Sanya	1	2.65	1.32
N222601	2019	Sanya	2	2.44	1.35
N222601	2019	Sanya	3	3.13	1.34
N222602	2019	Sanya	1	2.43	2.11
N222602	2019	Sanya	2	2.46	1.54
N222602	2019	Sanya	3	2.4	1.99
N222603	2019	Sanya	1	1.8	1.89
N222603	2019	Sanya	2	1.84	1.26
N222603	2019	Sanya	3	-	-
N222604	2019	Sanya	1	2.96	1.54
N222604	2019	Sanya	2	2.09	1.43
N222604	2019	Sanya	3	2.79	2.09
N222605	2019	Sanya	1	3.22	2.04
N222605	2019	Sanya	2	2.88	2.16
N222605	2019	Sanya	3	2.97	1.83

Line	Year	Location	Replication	SD (mm)	BD (mm)
N222606	2019	Sanya	1	2	2.31
N222606	2019	Sanya	2	3.43	2.24
N222606	2019	Sanya	3	2.47	1.77
N222607	2019	Sanya	1	2.61	2.2
N222607	2019	Sanya	2	3.11	2.62
N222607	2019	Sanya	3	2.03	1.83
N222608	2019	Sanya	1	1.36	1.87
N222608	2019	Sanya	2	3.32	2.23
N222608	2019	Sanya	3	2.55	2.23
N222609	2019	Sanya	1	1.83	1.53
N222609	2019	Sanya	2	1.91	1.61
N222609	2019	Sanya	3	2.12	1.84
N222610	2019	Sanya	1	2.78	1.76
N222610	2019	Sanya	2	2.62	1.15
N222610	2019	Sanya	3	2.51	1.79
N222611	2019	Sanya	1	2.35	1.6
N222611	2019	Sanya	2	2.29	1.36
N222611	2019	Sanya	3	2.22	1.67
N222612	2019	Sanya	1	3.04	1.73
N222612	2019	Sanya	2	2.48	1.4
N222612	2019	Sanya	3	2.46	1.9
N222613	2019	Sanya	1	1.83	1.36
N222613	2019	Sanya	2	2.19	1.71
N222613	2019	Sanya	3	2.2	1.53
N222614	2019	Sanya	1	2.65	2.21
N222614	2019	Sanya	2	2.18	1.84
N222614	2019	Sanya	3	3.03	1.85
N222615	2019	Sanya	1	1.49	1.46
N222615	2019	Sanya	2	1.47	1.47
N222615	2019	Sanya	3	1.48	1.13
N222616	2019	Sanya	1	2.08	1.57
N222616	2019	Sanya	2	2.66	1.74
N222616	2019	Sanya	3	3.06	1.91
N222617	2019	Sanya	1	1.81	1.47
N222617	2019	Sanya	2	2.06	1.78
N222617	2019	Sanya	3	3.22	2.86
N222618	2019	Sanya	1	-	-
N222618	2019	Sanya	2	2.63	1.58
N222618	2019	Sanya	3	2.1	1.57
N222619	2019	Sanya	1	1.81	1.48
N222619	2019	Sanya	2	1.88	1.81
N222619	2019	Sanya	3	1.62	1.67
N222620	2019	Sanya	1	1.64	1.26
N222620	2019	Sanya	2	1.34	1.02
N222620	2019	Sanya	3	1.77	1.24

Line	Year	Location	Replication	SD (mm)	BD (mm)
N222621	2019	Sanya	1	1.74	1.68
N222621	2019	Sanya	2	3.34	1.77
N222621	2019	Sanya	3	2.33	1.89
N222622	2019	Sanya	1	1.92	1.48
N222622	2019	Sanya	2	1.95	1.57
N222622	2019	Sanya	3	1.75	1.33
N222623	2019	Sanya	1	1.55	1.22
N222623	2019	Sanya	2	1.61	1.4
N222623	2019	Sanya	3	1.67	1.25
N222624	2019	Sanya	1	1.77	1.21
N222624	2019	Sanya	2	1.79	1.19
N222624	2019	Sanya	3	1.62	1.27
N222625	2019	Sanya	1	2.17	1.62
N222625	2019	Sanya	2	2.3	1.72
N222625	2019	Sanya	3	2.32	1.36
N222628	2019	Sanya	1	2.08	2.17
N222628	2019	Sanya	2	2.04	1.47
N222628	2019	Sanya	3	1.95	1.38
N222629	2019	Sanya	1	2.41	1.51
N222629	2019	Sanya	2	2.3	1.99
N222629	2019	Sanya	3	2.2	1.74
N222630	2019	Sanya	1	2.07	1.44
N222630	2019	Sanya	2	1.95	1.93
N222630	2019	Sanya	3	1.92	1.61
N222631	2019	Sanya	1	2.21	1.84
N222631	2019	Sanya	2	1.7	1.24
N222631	2019	Sanya	3	2.11	1.65
N222632	2019	Sanya	1	2.04	1.68
N222632	2019	Sanya	2	2.3	2.25
N222632	2019	Sanya	3	1.91	1.69
N222633	2019	Sanya	1	1.95	1.55
N222633	2019	Sanya	2	2.3	1.67
N222633	2019	Sanya	3	2.97	1.91
N222634	2019	Sanya	1	2	1.86
N222634	2019	Sanya	2	2.49	1.29
N222634	2019	Sanya	3	2.07	1.37
N222635	2019	Sanya	1	1.39	1.55
N222635	2019	Sanya	2	1.87	2.08
N222635	2019	Sanya	3	2.86	2.08
N222636	2019	Sanya	1	1.97	0.97
N222636	2019	Sanya	2	2.26	1.69
N222636	2019	Sanya	3	2.12	1.56
N222637	2019	Sanya	1	2.68	1.41
N222637	2019	Sanya	2	2.69	3.04
N222637	2019	Sanya	3	2.03	2.1

Line	Year	Location	Replication	SD (mm)	BD (mm)
N222638	2019	Sanya	1	-	-
N222638	2019	Sanya	2	-	-
N222638	2019	Sanya	3	-	-
N222639	2019	Sanya	1	2.29	1.64
N222639	2019	Sanya	2	2.63	1.78
N222639	2019	Sanya	3	2.54	1.38
N222640	2019	Sanya	1	3.09	2.54
N222640	2019	Sanya	2	3.18	2.76
N222640	2019	Sanya	3	2.28	2.37
N222641	2019	Sanya	1	2.55	1.15
N222641	2019	Sanya	2	2.49	1.21
N222641	2019	Sanya	3	1.77	0.98
N222642	2019	Sanya	1	2.56	1.3
N222642	2019	Sanya	2	3.15	2.36
N222642	2019	Sanya	3	2.5	2
N222643	2019	Sanya	1	1.77	-
N222643	2019	Sanya	2	2.49	1.86
N222643	2019	Sanya	3	2.01	1.51
N222644	2019	Sanya	1	2.79	1.39
N222644	2019	Sanya	2	2.42	1.38
N222644	2019	Sanya	3	2.37	1.57
N222645	2019	Sanya	1	1.92	1.57
N222645	2019	Sanya	2	2.04	1.67
N222645	2019	Sanya	3	2.21	1.72
N222646	2019	Sanya	1	2.41	1.72
N222646	2019	Sanya	2	1.92	1.41
N222646	2019	Sanya	3	1.79	1.5
N222647	2019	Sanya	1	2.64	1.61
N222647	2019	Sanya	2	2.16	1.44
N222647	2019	Sanya	3	2.28	1.42
N222648	2019	Sanya	1	1.83	1.19
N222648	2019	Sanya	2	2.28	1.85
N222648	2019	Sanya	3	2.11	1.3
N222649	2019	Sanya	1	2.09	0.97
N222649	2019	Sanya	2	1.66	0.88
N222649	2019	Sanya	3	1.66	0.84
N222650	2019	Sanya	1	1.73	1.86
N222650	2019	Sanya	2	1.87	1.91
N222650	2019	Sanya	3	2.19	1.6
N222651	2019	Sanya	1	3.18	2.18
N222651	2019	Sanya	2	3.17	1.98
N222651	2019	Sanya	3	3.3	2.23
N222652	2019	Sanya	1	1.97	0.85
N222652	2019	Sanya	2	2.72	1.8
N222652	2019	Sanya	3	2.87	2.2

Line	Year	Location	Replication	SD (mm)	BD (mm)
N222653	2019	Sanya	1	2.5	1.73
N222653	2019	Sanya	2	2.68	1.86
N222653	2019	Sanya	3	1.42	1.58
N222654	2019	Sanya	1	3.38	2.01
N222654	2019	Sanya	2	4.18	2.26
N222654	2019	Sanya	3	2.83	2.03
N222655	2019	Sanya	1	1.36	2.28
N222655	2019	Sanya	2	1.39	-
N222655	2019	Sanya	3	2.13	1.56
N222656	2019	Sanya	1	1.29	1.12
N222656	2019	Sanya	2	2.16	1.26
N222656	2019	Sanya	3	2.71	1.8
N222657	2019	Sanya	1	2.31	1.55
N222657	2019	Sanya	2	1.7	1.51
N222657	2019	Sanya	3	1.83	1.36
N222658	2019	Sanya	1	1.61	1.57
N222658	2019	Sanya	2	2.49	2.05
N222658	2019	Sanya	3	2.36	1.66
N222659	2019	Sanya	1	3.47	2.31
N222659	2019	Sanya	2	3.4	2.28
N222659	2019	Sanya	3	3.7	2.46
N222660	2019	Sanya	1	1.97	2.19
N222660	2019	Sanya	2	2.14	1.96
N222660	2019	Sanya	3	2.13	2.13
N222661	2019	Sanya	1	1.31	1.41
N222661	2019	Sanya	2	1.7	1.65
N222661	2019	Sanya	3	1.51	1.73
N222662	2019	Sanya	1	2.24	2.29
N222662	2019	Sanya	2	4.32	2.22
N222662	2019	Sanya	3	2.77	2.05
N222663	2019	Sanya	1	3.27	2.07
N222663	2019	Sanya	2	3.89	2.19
N222663	2019	Sanya	3	3.04	2.47
N222664	2019	Sanya	1	1.08	1.46
N222664	2019	Sanya	2	1.17	1.09
N222664	2019	Sanya	3	1.05	1.3
N222665	2019	Sanya	1	2.62	2.08
N222665	2019	Sanya	2	2.22	1.33
N222665	2019	Sanya	3	2.28	1.54
N222666	2019	Sanya	1	2.83	1.65
N222666	2019	Sanya	2	3.23	1.63
N222666	2019	Sanya	3	3.19	1.75
N222667	2019	Sanya	1	3.48	2.39
N222667	2019	Sanya	2	2.56	1.23
N222667	2019	Sanya	3	2.49	2.02

Line	Year	Location	Replication	SD (mm)	BD (mm)
N222668	2019	Sanya	1	1.79	1.6
N222668	2019	Sanya	2	1.34	1.51
N222668	2019	Sanya	3	1.08	0.67
N222669	2019	Sanya	1	2.06	1.07
N222669	2019	Sanya	2	1.51	1.05
N222669	2019	Sanya	3	1.98	1.71
N222670	2019	Sanya	1	1.85	1.94
N222670	2019	Sanya	2	2.08	1.89
N222670	2019	Sanya	3	2.27	1.66
N222671	2019	Sanya	1	2.3	1.65
N222671	2019	Sanya	2	2.65	1.91
N222671	2019	Sanya	3	2.75	2.13
N222672	2019	Sanya	1	1.73	1.96
N222672	2019	Sanya	2	2.14	2.33
N222672	2019	Sanya	3	3.33	2.45
N222673	2019	Sanya	1	2.08	2.22
N222673	2019	Sanya	2	2.03	1.33
N222673	2019	Sanya	3	1.45	-
N222674	2019	Sanya	1	2.12	1.25
N222674	2019	Sanya	2	1.71	1.1
N222674	2019	Sanya	3	2.02	1.75
N222675	2019	Sanya	1	1.96	1.89
N222675	2019	Sanya	2	2.19	2.25
N222675	2019	Sanya	3	2.51	1.82
N222676	2019	Sanya	1	1.8	1.24
N222676	2019	Sanya	2	2.56	2.7
N222676	2019	Sanya	3	2.4	1.83
N222677	2019	Sanya	1	2.27	2.01
N222677	2019	Sanya	2	1.95	1.49
N222677	2019	Sanya	3	2.66	1.89
N222678	2019	Sanya	1	2.16	1.47
N222678	2019	Sanya	2	2.08	1.6
N222678	2019	Sanya	3	1.71	1.32
N222679	2019	Sanya	1	1.78	1.92
N222679	2019	Sanya	2	2.37	2.42
N222679	2019	Sanya	3	2.35	2.06
N222680	2019	Sanya	1	3.39	2.28
N222680	2019	Sanya	2	3.07	1.74
N222680	2019	Sanya	3	3.09	2.32
N222681	2019	Sanya	1	3.57	2.68
N222681	2019	Sanya	2	2.61	2.5
N222681	2019	Sanya	3	2.67	2.43
N222682	2019	Sanya	1	3.93	2.71
N222682	2019	Sanya	2	2.34	1.78
N222682	2019	Sanya	3	2.4	1.81

Line	Year	Location	Replication	SD (mm)	BD (mm)
N222683	2019	Sanya	1	3.39	2.35
N222683	2019	Sanya	2	3.27	2.11
N222683	2019	Sanya	3	3.34	2.04
N222684	2019	Sanya	1	2.99	1.32
N222684	2019	Sanya	2	3.15	1.46
N222684	2019	Sanya	3	3.01	1.92
N222685	2019	Sanya	1	3.9	2.87
N222685	2019	Sanya	2	2.91	2.29
N222685	2019	Sanya	3	3.06	2.28
N222686	2019	Sanya	1	3.29	2.22
N222686	2019	Sanya	2	3.22	2.29
N222686	2019	Sanya	3	3.56	2.58
N222687	2019	Sanya	1	1.94	1.53
N222687	2019	Sanya	2	1.93	2.11
N222687	2019	Sanya	3	2.67	1.75
N222688	2019	Sanya	1	1.55	1.27
N222688	2019	Sanya	2	1.73	1.57
N222688	2019	Sanya	3	1.94	1.15
N222689	2019	Sanya	1	2.21	1.67
N222689	2019	Sanya	2	2.18	1.87
N222689	2019	Sanya	3	2.39	1.66
N222690	2019	Sanya	1	2.6	2.18
N222690	2019	Sanya	2	2.23	2.42
N222690	2019	Sanya	3	2.52	2.46
N222691	2019	Sanya	1	2.22	1.69
N222691	2019	Sanya	2	2.01	1.82
N222691	2019	Sanya	3	1.78	1.65
N222692	2019	Sanya	1	1.84	1.73
N222692	2019	Sanya	2	1.98	1.72
N222692	2019	Sanya	3	1.82	1.54
N222693	2019	Sanya	1	2.19	1.99
N222693	2019	Sanya	2	3.04	2.03
N222693	2019	Sanya	3	2.66	1.89
N222694	2019	Sanya	1	1.61	1.11
N222694	2019	Sanya	2	1.61	1.05
N222694	2019	Sanya	3	1.29	1.02
N222695	2019	Sanya	1	2.19	2.12
N222695	2019	Sanya	2	1.92	2.05
N222695	2019	Sanya	3	1.58	2.07
N222696	2019	Sanya	1	1.89	1.51
N222696	2019	Sanya	2	1.75	1.65
N222696	2019	Sanya	3	1.53	1.15
N222697	2019	Sanya	1	1.97	1.7
N222697	2019	Sanya	2	1.76	2.1
N222697	2019	Sanya	3	1.58	1.73

Line	Year	Location	Replication	SD (mm)	BD (mm)
N222698	2019	Sanya	1	2.16	1.44
N222698	2019	Sanya	2	1.58	1.33
N222698	2019	Sanya	3	1.52	1.39
N222699	2019	Sanya	1	1.62	2.37
N222699	2019	Sanya	2	1.38	2.48
N222699	2019	Sanya	3	1.65	2.17
N222700	2019	Sanya	1	2.93	2.2
N222700	2019	Sanya	2	3.07	1.88
N222700	2019	Sanya	3	3.44	2.17
N222701	2019	Sanya	1	3.66	3.07
N222701	2019	Sanya	2	2.47	3.03
N222701	2019	Sanya	3	3.17	2.17
N222702	2019	Sanya	1	3	1.59
N222702	2019	Sanya	2	1.28	1.52
N222702	2019	Sanya	3	1.84	1.12
N222703	2019	Sanya	1	-	-
N222703	2019	Sanya	2	1.39	1.92
N222703	2019	Sanya	3	2.03	2.06
N222704	2019	Sanya	1	2.12	1.2
N222704	2019	Sanya	2	2.05	1.48
N222704	2019	Sanya	3	2.56	1.76
N222705	2019	Sanya	1	1.84	1.13
N222705	2019	Sanya	2	2.22	1.85
N222705	2019	Sanya	3	-	1.55
N222706	2019	Sanya	1	2.98	2.46
N222706	2019	Sanya	2	2.99	1.81
N222706	2019	Sanya	3	2.71	2.52
N222707	2019	Sanya	1	2.27	1.02
N222707	2019	Sanya	2	2.15	1.29
N222707	2019	Sanya	3	2.19	1.36
N222708	2019	Sanya	1	1.74	0.81
N222708	2019	Sanya	2	2.03	1.05
N222708	2019	Sanya	3	1.21	1.25
N222709	2019	Sanya	1	1.85	1.67
N222709	2019	Sanya	2	1.88	1.32
N222709	2019	Sanya	3	1.76	1.43
N222710	2019	Sanya	1	3.22	2.43
N222710	2019	Sanya	2	2.1	2.44
N222710	2019	Sanya	3	2.97	2.66
N222711	2019	Sanya	1	2.73	1.88
N222711	2019	Sanya	2	3.38	2.24
N222711	2019	Sanya	3	2.47	1.49
N222712	2019	Sanya	1	2.07	1.94
N222712	2019	Sanya	2	2.46	2.29
N222712	2019	Sanya	3	1.66	1.98

Line	Year	Location	Replication	SD (mm)	BD (mm)
N222713	2019	Sanya	1	2.58	1.83
N222713	2019	Sanya	2	2.17	1.59
N222713	2019	Sanya	3	2.09	1.89
N222715	2019	Sanya	1	2.47	1.32
N222715	2019	Sanya	2	2.31	1.63
N222715	2019	Sanya	3	2.16	1.5
N222716	2019	Sanya	1	2.45	2.32
N222716	2019	Sanya	2	2.92	2.1
N222716	2019	Sanya	3	3.57	3
N222717	2019	Sanya	1	3.93	2.83
N222717	2019	Sanya	2	3.81	2.43
N222717	2019	Sanya	3	3.79	2.24
N222718	2019	Sanya	1	2.2	1.8
N222718	2019	Sanya	2	2.33	2.17
N222718	2019	Sanya	3	2.66	1.54
N222719	2019	Sanya	1	2.98	1.76
N222719	2019	Sanya	2	3.21	2.01
N222719	2019	Sanya	3	2.04	-
N222720	2019	Sanya	1	3.18	1.9
N222720	2019	Sanya	2	2.23	1.36
N222720	2019	Sanya	3	2.68	1.66
N222721	2019	Sanya	1	1.38	1.48
N222721	2019	Sanya	2	1.24	-
N222721	2019	Sanya	3	2.03	1.49
N222722	2019	Sanya	1	2.07	1.35
N222722	2019	Sanya	2	1.96	1.31
N222722	2019	Sanya	3	2.73	1.4
N222723	2019	Sanya	1	2.31	3.23
N222723	2019	Sanya	2	3.19	2.38
N222723	2019	Sanya	3	3.05	2.92
N222724	2019	Sanya	1	3.16	2.19
N222724	2019	Sanya	2	3.55	2.33
N222724	2019	Sanya	3	2.78	2.13
N222725	2019	Sanya	1	3.9	2.82
N222725	2019	Sanya	2	3.48	2.38
N222725	2019	Sanya	3	3.32	3.45
N222726	2019	Sanya	1	3.03	1.83
N222726	2019	Sanya	2	2.8	2.02
N222726	2019	Sanya	3	2.43	1.83
N222727	2019	Sanya	1	1.41	0.71
N222727	2019	Sanya	2	1.23	-
N222727	2019	Sanya	3	-	-
N222728	2019	Sanya	1	2.71	1.69
N222728	2019	Sanya	2	2.36	1.55
N222728	2019	Sanya	3	2.87	1.66

Line	Year	Location	Replication	SD (mm)	BD (mm)
N222729	2019	Sanya	1	2.04	1.54
N222729	2019	Sanya	2	3.6	1.85
N222729	2019	Sanya	3	2.63	1.9
N222730	2019	Sanya	1	2.94	2.23
N222730	2019	Sanya	2	2.64	2.15
N222730	2019	Sanya	3	3.19	2.05
N222731	2019	Sanya	1	2.58	1.8
N222731	2019	Sanya	2	1.94	1.67
N222731	2019	Sanya	3	1.78	1.34
N222732	2019	Sanya	1	1.55	1.59
N222732	2019	Sanya	2	1.43	1.93
N222732	2019	Sanya	3	1.47	1.55
N222733	2019	Sanya	1	3.38	3.19
N222733	2019	Sanya	2	1.96	2.14
N222733	2019	Sanya	3	4.46	2.73
N222734	2019	Sanya	1	4.36	2.48
N222734	2019	Sanya	2	4.96	2.93
N222734	2019	Sanya	3	3.42	2.94
N222735	2019	Sanya	1	2.88	2.37
N222735	2019	Sanya	2	2.25	2.32
N222735	2019	Sanya	3	2.56	2.14
N222736	2019	Sanya	1	2.41	1.86
N222736	2019	Sanya	2	2.55	3.03
N222736	2019	Sanya	3	2.79	2.06
N222737	2019	Sanya	1	2.69	2.24
N222737	2019	Sanya	2	2.13	2
N222737	2019	Sanya	3	2.06	1.72
N222738	2019	Sanya	1	3.29	1.68
N222738	2019	Sanya	2	1.95	1.66
N222738	2019	Sanya	3	2.23	1.48
N222739	2019	Sanya	1	3.36	1.38
N222739	2019	Sanya	2	2.6	1.68
N222739	2019	Sanya	3	2.78	1.83
N222740	2019	Sanya	1	2.87	2.02
N222740	2019	Sanya	2	2.74	1.59
N222740	2019	Sanya	3	2.78	1.43
N222741	2019	Sanya	1	2.37	2.27
N222741	2019	Sanya	2	2.3	1.98
N222741	2019	Sanya	3	2.26	2.12
N222742	2019	Sanya	1	1.08	-
N222742	2019	Sanya	2	1.64	1.28
N222742	2019	Sanya	3	-	-
N222743	2019	Sanya	1	2.69	2.07
N222743	2019	Sanya	2	2.21	1.87
N222743	2019	Sanya	3	2.87	2.47

Line	Year	Location	Replication	SD (mm)	BD (mm)
N222744	2019	Sanya	1	2.88	1.75
N222744	2019	Sanya	2	2.44	1.42
N222744	2019	Sanya	3	3.32	1.35
N222745	2019	Sanya	1	2.66	1.64
N222745	2019	Sanya	2	2.49	2.03
N222745	2019	Sanya	3	3.05	1.67
N222746	2019	Sanya	1	2.42	1.97
N222746	2019	Sanya	2	1.43	1.51
N222746	2019	Sanya	3	1.54	1.2
N222747	2019	Sanya	1	2.84	3
N222747	2019	Sanya	2	3.38	1.94
N222747	2019	Sanya	3	3.78	2.19
N222748	2019	Sanya	1	2.28	1.33
N222748	2019	Sanya	2	2.11	2.16
N222748	2019	Sanya	3	2.89	1.87
N222749	2019	Sanya	1	2.24	1.76
N222749	2019	Sanya	2	2.8	1.7
N222749	2019	Sanya	3	2.64	1.59
N222750	2019	Sanya	1	1.69	1.51
N222750	2019	Sanya	2	1.72	1.07
N222750	2019	Sanya	3	2.09	2.02
N222751	2019	Sanya	1	2.85	2.05
N222751	2019	Sanya	2	3.61	2.78
N222751	2019	Sanya	3	3.8	2.72
N222752	2019	Sanya	1	1.95	1.26
N222752	2019	Sanya	2	2.19	1.32
N222752	2019	Sanya	3	2.33	1.28
N222753	2019	Sanya	1	2.61	1.48
N222753	2019	Sanya	2	3.03	1.76
N222753	2019	Sanya	3	2.32	1.69
N222754	2019	Sanya	1	1.38	1.49
N222754	2019	Sanya	2	1.97	1.7
N222754	2019	Sanya	3	1.73	1.62
N222755	2019	Sanya	1	3.63	1.67
N222755	2019	Sanya	2	2.68	1.51
N222755	2019	Sanya	3	2.32	1.3
N222756	2019	Sanya	1	2.94	2.06
N222756	2019	Sanya	2	3.1	1.64
N222756	2019	Sanya	3	3.46	1.83
N222757	2019	Sanya	1	3.23	2.38
N222757	2019	Sanya	2	3.67	2.35
N222757	2019	Sanya	3	3.98	2.43
N222759	2019	Sanya	1	2.76	2.16
N222759	2019	Sanya	2	3.69	2.16
N222759	2019	Sanya	3	1.89	1.79

Line	Year	Location	Replication	SD (mm)	BD (mm)
N222760	2019	Sanya	1	2.71	2.35
N222760	2019	Sanya	2	2.56	2.47
N222760	2019	Sanya	3	3.19	1.93
N222761	2019	Sanya	1	1.98	1.49
N222761	2019	Sanya	2	1.88	1.8
N222761	2019	Sanya	3	1.95	2.41
N222762	2019	Sanya	1	2.57	2.08
N222762	2019	Sanya	2	2.42	1.61
N222762	2019	Sanya	3	2.37	1.37
N222763	2019	Sanya	1	2.41	1.94
N222763	2019	Sanya	2	2.62	1.56
N222763	2019	Sanya	3	2.8	1.79
N222764	2019	Sanya	1	1.99	1.63
N222764	2019	Sanya	2	1.66	1.61
N222764	2019	Sanya	3	2.58	2.11
N222765	2019	Sanya	1	2.23	1.75
N222765	2019	Sanya	2	1.78	1.56
N222765	2019	Sanya	3	2.02	1.58
N222766	2019	Sanya	1	1.89	1.72
N222766	2019	Sanya	2	2.36	1.94
N222766	2019	Sanya	3	1.53	1.74
N222767	2019	Sanya	1	2.38	1.23
N222767	2019	Sanya	2	2.01	1.59
N222767	2019	Sanya	3	2.38	1.97
N222768	2019	Sanya	1	1.69	1.22
N222768	2019	Sanya	2	-	-
N222768	2019	Sanya	3	-	-
N222769	2019	Sanya	1	3.24	1.88
N222769	2019	Sanya	2	2.98	1.92
N222769	2019	Sanya	3	3.39	1.85
N222770	2019	Sanya	1	2.23	2.05
N222770	2019	Sanya	2	2.44	1.48
N222770	2019	Sanya	3	3.18	1.77
N222771	2019	Sanya	1	2.36	1.52
N222771	2019	Sanya	2	2.48	1.85
N222771	2019	Sanya	3	1.12	1.95
N222772	2019	Sanya	1	1.43	1.56
N222772	2019	Sanya	2	1.25	1.49
N222772	2019	Sanya	3	1.86	1.57
N222773	2019	Sanya	1	1.99	1.6
N222773	2019	Sanya	2	1.14	1.19
N222773	2019	Sanya	3	1.75	1.77
N222774	2019	Sanya	1	1.49	1.93
N222774	2019	Sanya	2	-	-
N222774	2019	Sanya	3	1.82	1.66

Line	Year	Location	Replication	SD (mm)	BD (mm)
N222775	2019	Sanya	1	2.88	1.53
N222775	2019	Sanya	2	2.42	2.16
N222775	2019	Sanya	3	2.69	1.6
N222776	2019	Sanya	1	1.68	1.75
N222776	2019	Sanya	2	1.73	0.95
N222776	2019	Sanya	3	1.55	1.58
N222777	2019	Sanya	1	1.88	1.41
N222777	2019	Sanya	2	1.59	0.88
N222777	2019	Sanya	3	1.94	1.54
N222778	2019	Sanya	1	2.31	1.85
N222778	2019	Sanya	2	2.24	2.04
N222778	2019	Sanya	3	2.6	1.82
N222779	2019	Sanya	1	1.96	1.33
N222779	2019	Sanya	2	3.13	1.88
N222779	2019	Sanya	3	2.17	1.16
N222780	2019	Sanya	1	3.73	2.95
N222780	2019	Sanya	2	2.61	2.34
N222780	2019	Sanya	3	2.69	2.05
N222781	2019	Sanya	1	1.44	1.83
N222781	2019	Sanya	2	2.26	1.42
N222781	2019	Sanya	3	1.82	1.17
N222782	2019	Sanya	1	3.2	1.9
N222782	2019	Sanya	2	3.39	1.51
N222782	2019	Sanya	3	3.17	2.54
N222783	2019	Sanya	1	-	-
N222783	2019	Sanya	2	-	-
N222783	2019	Sanya	3	-	-
N222784	2019	Sanya	1	3.01	2.14
N222784	2019	Sanya	2	2.94	2.36
N222784	2019	Sanya	3	2.16	2.19
N222785	2019	Sanya	1	1.59	0.89
N222785	2019	Sanya	2	1.62	1
N222785	2019	Sanya	3	1.1	0.88
N222786	2019	Sanya	1	2.71	2.09
N222786	2019	Sanya	2	2.51	1.82
N222786	2019	Sanya	3	2.53	1.72
N222787	2019	Sanya	1	3.02	1.91
N222787	2019	Sanya	2	2.87	2.2
N222787	2019	Sanya	3	2.64	2.02
N222788	2019	Sanya	1	2.47	2.16
N222788	2019	Sanya	2	2.05	1.94
N222788	2019	Sanya	3	1.89	1.9
N222789	2019	Sanya	1	4.27	2.31
N222789	2019	Sanya	2	3.18	2.18
N222789	2019	Sanya	3	3.92	2.83

Line	Year	Location	Replication	SD (mm)	BD (mm)
N222790	2019	Sanya	1	2.22	2.19
N222790	2019	Sanya	2	1.9	2.13
N222790	2019	Sanya	3	2.18	2.17
N222791	2019	Sanya	1	2.03	2.62
N222791	2019	Sanya	2	2.48	3.41
N222791	2019	Sanya	3	3.24	3.2
N222792	2019	Sanya	1	2.69	1.56
N222792	2019	Sanya	2	2.42	0.93
N222792	2019	Sanya	3	2.29	1.41
N222793	2019	Sanya	1	1.9	1.43
N222793	2019	Sanya	2	2.37	1.52
N222793	2019	Sanya	3	3.28	2.31
N222794	2019	Sanya	1	2.76	2.18
N222794	2019	Sanya	2	1.74	1.66
N222794	2019	Sanya	3	2.24	1.88
N222795	2019	Sanya	1	3.68	2.64
N222795	2019	Sanya	2	2.94	2.22
N222795	2019	Sanya	3	2.22	2.06
N222796	2019	Sanya	1	0.58	1.22
N222796	2019	Sanya	2	2.03	1.27
N222796	2019	Sanya	3	1.95	2.07
N222797	2019	Sanya	1	2.22	2.28
N222797	2019	Sanya	2	2.91	2.04
N222797	2019	Sanya	3	1.95	2.03
N222798	2019	Sanya	1	2.25	1.99
N222798	2019	Sanya	2	1.66	2.01
N222798	2019	Sanya	3	2.29	2.25
N222799	2019	Sanya	1	3.07	1.85
N222799	2019	Sanya	2	3.13	1.43
N222799	2019	Sanya	3	2.7	2.29
N222800	2019	Sanya	1	1.56	1.19
N222800	2019	Sanya	2	1.53	0.85
N222800	2019	Sanya	3	1.66	1.37
N222801	2019	Sanya	1	3.55	2.3
N222801	2019	Sanya	2	3.9	2.41
N222801	2019	Sanya	3	3.78	2.44
N222802	2019	Sanya	1	1.93	2.05
N222802	2019	Sanya	2	2.26	1.79
N222802	2019	Sanya	3	2.81	2.79
N222803	2019	Sanya	1	2.13	1.47
N222803	2019	Sanya	2	2.62	1.96
N222803	2019	Sanya	3	2.37	1.6
N222804	2019	Sanya	1	1.89	1.89
N222804	2019	Sanya	2	2.24	1.78
N222804	2019	Sanya	3	1.95	2.02

Line	Year	Location	Replication	SD (mm)	BD (mm)
N222805	2019	Sanya	1	2.93	1.78
N222805	2019	Sanya	2	1.98	1.57
N222805	2019	Sanya	3	1.65	1.15
N222806	2019	Sanya	1	1.9	1.06
N222806	2019	Sanya	2	1.6	1.28
N222806	2019	Sanya	3	1.87	1.18
N222807	2019	Sanya	1	2.79	1.74
N222807	2019	Sanya	2	2.86	1.43
N222807	2019	Sanya	3	2.07	1.44
N222808	2019	Sanya	1	3.29	2.28
N222808	2019	Sanya	2	3.02	2.37
N222808	2019	Sanya	3	3	2.08
N222809	2019	Sanya	1	1.75	2.08
N222809	2019	Sanya	2	1.82	1.35
N222809	2019	Sanya	3	1.68	1.86
N222810	2019	Sanya	1	1.5	0.83
N222810	2019	Sanya	2	1.78	1.11
N222810	2019	Sanya	3	1.99	1.12
N222811	2019	Sanya	1	1.77	1.47
N222811	2019	Sanya	2	1.75	1.74
N222811	2019	Sanya	3	1.27	0.79
N222812	2019	Sanya	1	1.61	1.89
N222812	2019	Sanya	2	1.62	1.28
N222812	2019	Sanya	3	1.59	1.36
N222813	2019	Sanya	1	-	-
N222813	2019	Sanya	2	3.1	1.91
N222813	2019	Sanya	3	2.38	1.88
N222814	2019	Sanya	1	2.77	1.95
N222814	2019	Sanya	2	2.55	2.89
N222814	2019	Sanya	3	3.07	1.82
N222815	2019	Sanya	1	2.29	1.54
N222815	2019	Sanya	2	2.72	2.21
N222815	2019	Sanya	3	2.98	1.95
N222816	2019	Sanya	1	2.24	1.74
N222816	2019	Sanya	2	1.54	2.14
N222816	2019	Sanya	3	1.98	1.86
N222817	2019	Sanya	1	2.18	2.27
N222817	2019	Sanya	2	2.2	2.15
N222817	2019	Sanya	3	3.09	2.41
N222818	2019	Sanya	1	2.61	1.97
N222818	2019	Sanya	2	2.89	1.74
N222818	2019	Sanya	3	2.47	1.4
N222819	2019	Sanya	1	2.2	1.8
N222819	2019	Sanya	2	2.67	1.85
N222819	2019	Sanya	3	2.13	2.24

Line	Year	Location	Replication	SD (mm)	BD (mm)
N222820	2019	Sanya	1	2.77	1.49
N222820	2019	Sanya	2	3.11	1.71
N222820	2019	Sanya	3	3.07	1.79
N222821	2019	Sanya	1	1.87	1.33
N222821	2019	Sanya	2	1.72	1.43
N222821	2019	Sanya	3	1.82	2.09
N222822	2019	Sanya	1	1.84	1.9
N222822	2019	Sanya	2	2.84	1.71
N222822	2019	Sanya	3	2.41	1.69
N222823	2019	Sanya	1	2.72	1.89
N222823	2019	Sanya	2	2.73	1.14
N222823	2019	Sanya	3	2.36	1.5
N222824	2019	Sanya	1	1.11	1.29
N222824	2019	Sanya	2	1.59	1.47
N222824	2019	Sanya	3	2.26	1.36
N222825	2019	Sanya	1	1.87	1.21
N222825	2019	Sanya	2	1.49	1.56
N222825	2019	Sanya	3	1.5	1.67
N222826	2019	Sanya	1	1.83	1.44
N222826	2019	Sanya	2	1.77	1.75
N222826	2019	Sanya	3	1.73	1.02
N222827	2019	Sanya	1	1.79	1.53
N222827	2019	Sanya	2	2.18	1.56
N222827	2019	Sanya	3	2.31	1.39
N222828	2019	Sanya	1	2.26	1.76
N222828	2019	Sanya	2	1.78	-
N222828	2019	Sanya	3	1.76	2.01
N222829	2019	Sanya	1	3.01	2.12
N222829	2019	Sanya	2	2.94	1.9
N222829	2019	Sanya	3	2.29	1.5
N222830	2019	Sanya	1	2.31	1.24
N222830	2019	Sanya	2	1.71	1.84
N222830	2019	Sanya	3	1.95	1.76
N222831	2019	Sanya	1	1.8	1.41
N222831	2019	Sanya	2	1.67	1.5
N222831	2019	Sanya	3	2.67	1.65
N222832	2019	Sanya	1	2.43	1.8
N222832	2019	Sanya	2	2.27	1.73
N222832	2019	Sanya	3	2.71	2.25
N222833	2019	Sanya	1	3.76	2.9
N222833	2019	Sanya	2	4.04	2.83
N222833	2019	Sanya	3	3.72	2.89
N222834	2019	Sanya	1	1.94	1.41
N222834	2019	Sanya	2	1.78	1.2
N222834	2019	Sanya	3	1.52	0.98

Line	Year	Location	Replication	SD (mm)	BD (mm)
N222835	2019	Sanya	1	4.41	2.46
N222835	2019	Sanya	2	2.27	1.91
N222835	2019	Sanya	3	2.92	2.09
N222836	2019	Sanya	1	2.26	1.86
N222836	2019	Sanya	2	2.68	1.32
N222836	2019	Sanya	3	2.43	1.48
N222837	2019	Sanya	1	2.7	1.47
N222837	2019	Sanya	2	2.29	1.42
N222837	2019	Sanya	3	2.53	1.65
N222838	2019	Sanya	1	1.53	1.35
N222838	2019	Sanya	2	1.92	1.53
N222838	2019	Sanya	3	1.67	1.36
N222839	2019	Sanya	1	2.12	1.89
N222839	2019	Sanya	2	1.88	1.72
N222839	2019	Sanya	3	2.26	1.57
N222840	2019	Sanya	1	2.51	1.95
N222840	2019	Sanya	2	2.61	2.07
N222840	2019	Sanya	3	3.07	2.93
N222841	2019	Sanya	1	2.33	1.67
N222841	2019	Sanya	2	2.12	1.48
N222841	2019	Sanya	3	1.39	1.49
N222842	2019	Sanya	1	1.46	1.07
N222842	2019	Sanya	2	1.98	1.45
N222842	2019	Sanya	3	1.64	1.13
N222843	2019	Sanya	1	2.94	2.19
N222843	2019	Sanya	2	3.12	2.6
N222843	2019	Sanya	3	2.7	2.25
N222844	2019	Sanya	1	2.45	1.43
N222844	2019	Sanya	2	1.85	1.44
N222844	2019	Sanya	3	2.74	1.76
N222845	2019	Sanya	1	2.01	1.28
N222845	2019	Sanya	2	2.14	2.32
N222845	2019	Sanya	3	1.89	2.04
N222846	2019	Sanya	1	2.31	2
N222846	2019	Sanya	2	2.38	2.9
N222846	2019	Sanya	3	2.37	3.16
N222847	2019	Sanya	1	2.42	1.9
N222847	2019	Sanya	2	2.64	1.67
N222847	2019	Sanya	3	2.18	1.84
N222848	2019	Sanya	1	1.6	1.79
N222848	2019	Sanya	2	2.48	1.7
N222848	2019	Sanya	3	2.19	2
N222849	2019	Sanya	1	2.45	1.86
N222849	2019	Sanya	2	1.77	1.72
N222849	2019	Sanya	3	2.12	1.53

Line	Year	Location	Replication	SD (mm)	BD (mm)
N222850	2019	Sanya	1	3.12	2.12
N222850	2019	Sanya	2	2.68	2.38
N222850	2019	Sanya	3	4.99	3.09
N222851	2019	Sanya	1	-	-
N222851	2019	Sanya	2	-	-
N222851	2019	Sanya	3	-	-
N222852	2019	Sanya	1	1.75	2.32
N222852	2019	Sanya	2	2.19	2.03
N222852	2019	Sanya	3	2.7	1.55
N222853	2019	Sanya	1	2.02	1.56
N222853	2019	Sanya	2	2.12	1.49
N222853	2019	Sanya	3	2.02	1.57
N222854	2019	Sanya	1	1.6	1.67
N222854	2019	Sanya	2	2.04	1.52
N222854	2019	Sanya	3	1.79	1.84
ZD41	2019	Sanya	1	4.38	2.59
ZD41	2019	Sanya	2	3.95	2.51
ZD41	2019	Sanya	3	4.05	2.37
ZYD02878	2019	Sanya	1	1.15	1.06
ZYD02878	2019	Sanya	2	0.91	1.83
ZYD02878	2019	Sanya	3	1.02	-
N222488	2020	JingZhou	1	4.34	1.98
N222488	2020	JingZhou	2	3.55	1.78
N222488	2020	JingZhou	3	4.22	2.11
N222489	2020	JingZhou	1	4.5	2.35
N222489	2020	JingZhou	2	3.76	2.17
N222489	2020	JingZhou	3	4.33	2
N222490	2020	JingZhou	1	4.19	2.05
N222490	2020	JingZhou	2	3.71	1.83
N222490	2020	JingZhou	3	2.54	1.69
N222491	2020	JingZhou	1	3.38	2.53
N222491	2020	JingZhou	2	4.14	2.16
N222491	2020	JingZhou	3	4.56	2.49
N222492	2020	JingZhou	1	3.46	1.73
N222492	2020	JingZhou	2	3.97	1.75
N222492	2020	JingZhou	3	1.94	1.66
N222493	2020	JingZhou	1	1.66	1.47
N222493	2020	JingZhou	2	1.15	-
N222493	2020	JingZhou	3	1.68	1.49
N222494	2020	JingZhou	1	4.17	2.23
N222494	2020	JingZhou	2	2.22	2.03
N222494	2020	JingZhou	3	3.8	1.97
N222495	2020	JingZhou	1	3.01	1.48
N222495	2020	JingZhou	2	2.11	1.44
N222495	2020	JingZhou	3	-	-

Line	Year	Location	Replication	SD (mm)	BD (mm)
N222496	2020	JingZhou	1	4.22	2.04
N222496	2020	JingZhou	2	4.23	2.34
N222496	2020	JingZhou	3	3.84	2.34
N222497	2020	JingZhou	1	2.98	2.3
N222497	2020	JingZhou	2	5.63	2.18
N222497	2020	JingZhou	3	4.8	2.3
N222498	2020	JingZhou	1	2.94	1.79
N222498	2020	JingZhou	2	3.22	2.15
N222498	2020	JingZhou	3	2.67	1.74
N222499	2020	JingZhou	1	3.23	2.09
N222499	2020	JingZhou	2	4.48	2.41
N222499	2020	JingZhou	3	4.83	3
N222500	2020	JingZhou	1	2.24	1.24
N222500	2020	JingZhou	2	2.99	1.91
N222500	2020	JingZhou	3	1.63	1.33
N222501	2020	JingZhou	1	1.76	1.45
N222501	2020	JingZhou	2	2.83	1.67
N222501	2020	JingZhou	3	2.41	1.21
N222502	2020	JingZhou	1	3.3	1.9
N222502	2020	JingZhou	2	3.86	1.99
N222502	2020	JingZhou	3	3.76	1.73
N222503	2020	JingZhou	1	4	2.46
N222503	2020	JingZhou	2	4.49	2.38
N222503	2020	JingZhou	3	2.99	2.33
N222504	2020	JingZhou	1	4.55	2.02
N222504	2020	JingZhou	2	5.75	2.27
N222504	2020	JingZhou	3	4.07	2
N222505	2020	JingZhou	1	-	-
N222505	2020	JingZhou	2	5.16	-
N222505	2020	JingZhou	3	4.68	-
N222506	2020	JingZhou	1	4.7	2.75
N222506	2020	JingZhou	2	3.76	2.02
N222506	2020	JingZhou	3	4.47	2.2
N222507	2020	JingZhou	1	4.4	2.86
N222507	2020	JingZhou	2	4.87	2.35
N222507	2020	JingZhou	3	3.77	2.15
N222508	2020	JingZhou	1	4.59	2.67
N222508	2020	JingZhou	2	5.98	2.68
N222508	2020	JingZhou	3	3.83	2.22
N222509	2020	JingZhou	1	5.23	2.8
N222509	2020	JingZhou	2	7.37	3
N222509	2020	JingZhou	3	6.22	2.62
N222510	2020	JingZhou	1	2.88	2.05
N222510	2020	JingZhou	2	3.08	2.25
N222510	2020	JingZhou	3	2.72	1.85

Line	Year	Location	Replication	SD (mm)	BD (mm)
N222511	2020	JingZhou	1	4.25	2.11
N222511	2020	JingZhou	2	3.7	2.01
N222511	2020	JingZhou	3	2.58	1.47
N222512	2020	JingZhou	1	2.71	1.67
N222512	2020	JingZhou	2	3.86	2.72
N222512	2020	JingZhou	3	3.9	2.55
N222513	2020	JingZhou	1	3.23	1.98
N222513	2020	JingZhou	2	-	-
N222513	2020	JingZhou	3	3.28	2.05
N222514	2020	JingZhou	1	4.53	2.48
N222514	2020	JingZhou	2	4.44	2.5
N222514	2020	JingZhou	3	4.23	2.15
N222515	2020	JingZhou	1	7.44	2.8
N222515	2020	JingZhou	2	6.37	2.87
N222515	2020	JingZhou	3	4.71	2.9
N222516	2020	JingZhou	1	4.27	2.46
N222516	2020	JingZhou	2	2.84	1.92
N222516	2020	JingZhou	3	3.63	2.12
N222517	2020	JingZhou	1	5.08	2.52
N222517	2020	JingZhou	2	5.25	2.53
N222517	2020	JingZhou	3	5.09	2.42
N222518	2020	JingZhou	1	3.42	2.13
N222518	2020	JingZhou	2	5.28	2.07
N222518	2020	JingZhou	3	4.08	2.05
N222519	2020	JingZhou	1	3.48	1.78
N222519	2020	JingZhou	2	3.05	2.11
N222519	2020	JingZhou	3	3.66	2.05
N222520	2020	JingZhou	1	4.41	2.3
N222520	2020	JingZhou	2	3.9	2.35
N222520	2020	JingZhou	3	3.25	2.31
N222521	2020	JingZhou	1	5.12	-
N222521	2020	JingZhou	2	8.84	3.18
N222521	2020	JingZhou	3	5.05	2.52
N222522	2020	JingZhou	1	4.07	2.33
N222522	2020	JingZhou	2	4.16	2.39
N222522	2020	JingZhou	3	3.68	2.3
N222523	2020	JingZhou	1	6.59	3.47
N222523	2020	JingZhou	2	8.36	4.2
N222523	2020	JingZhou	3	7.43	3.3
N222524	2020	JingZhou	1	5.14	3.03
N222524	2020	JingZhou	2	4.57	2.45
N222524	2020	JingZhou	3	7.87	3.23
N222525	2020	JingZhou	1	2.76	2.22
N222525	2020	JingZhou	2	3.31	2.58
N222525	2020	JingZhou	3	2.56	2.32

Line	Year	Location	Replication	SD (mm)	BD (mm)
N222526	2020	JingZhou	1	4.37	2
N222526	2020	JingZhou	2	3.82	2.05
N222526	2020	JingZhou	3	4.22	2.02
N222527	2020	JingZhou	1	5.11	2.18
N222527	2020	JingZhou	2	3.76	1.8
N222527	2020	JingZhou	3	3.33	1.79
N222528	2020	JingZhou	1	1.66	1.56
N222528	2020	JingZhou	2	5.88	2.06
N222528	2020	JingZhou	3	3.81	1.76
N222529	2020	JingZhou	1	5.83	3.18
N222529	2020	JingZhou	2	4	2.48
N222529	2020	JingZhou	3	5.99	3.15
N222530	2020	JingZhou	1	2.93	2.22
N222530	2020	JingZhou	2	4.73	2.22
N222530	2020	JingZhou	3	3.3	1.71
N222531	2020	JingZhou	1	3.15	1.93
N222531	2020	JingZhou	2	4.96	1.95
N222531	2020	JingZhou	3	5.23	2
N222532	2020	JingZhou	1	1.43	0.96
N222532	2020	JingZhou	2	1.28	0.51
N222532	2020	JingZhou	3	1.23	1.24
N222533	2020	JingZhou	1	3.89	1.85
N222533	2020	JingZhou	2	4.28	2.26
N222533	2020	JingZhou	3	2.58	1.98
N222534	2020	JingZhou	1	2.94	1.97
N222534	2020	JingZhou	2	3.63	2.08
N222534	2020	JingZhou	3	-	-
N222535	2020	JingZhou	1	2.41	1.26
N222535	2020	JingZhou	2	6.46	2.41
N222535	2020	JingZhou	3	3.73	2.02
N222536	2020	JingZhou	1	4.13	1.71
N222536	2020	JingZhou	2	2.97	1.75
N222536	2020	JingZhou	3	2.33	1.61
N222537	2020	JingZhou	1	4	2.2
N222537	2020	JingZhou	2	2.94	1.97
N222537	2020	JingZhou	3	3.16	2.37
N222538	2020	JingZhou	1	6.38	2.79
N222538	2020	JingZhou	2	7.13	3.01
N222538	2020	JingZhou	3	7.38	2.91
N222539	2020	JingZhou	1	4.85	2.57
N222539	2020	JingZhou	2	4.36	1.85
N222539	2020	JingZhou	3	4.56	1.95
N222540	2020	JingZhou	1	3.8	2.06
N222540	2020	JingZhou	2	7.65	2.75
N222540	2020	JingZhou	3	5.86	2.61

Line	Year	Location	Replication	SD (mm)	BD (mm)
N222541	2020	JingZhou	1	4.85	2.91
N222541	2020	JingZhou	2	4.6	2.58
N222541	2020	JingZhou	3	4.31	3.02
N222542	2020	JingZhou	1	3.05	1.69
N222542	2020	JingZhou	2	2.1	1.43
N222542	2020	JingZhou	3	2.59	1.42
N222543	2020	JingZhou	1	4.52	2.32
N222543	2020	JingZhou	2	5.87	2.26
N222543	2020	JingZhou	3	4.31	2.5
N222544	2020	JingZhou	1	2.92	1.81
N222544	2020	JingZhou	2	4.51	2.15
N222544	2020	JingZhou	3	4.17	1.95
N222545	2020	JingZhou	1	3.76	1.84
N222545	2020	JingZhou	2	3.9	2
N222545	2020	JingZhou	3	4.53	1.73
N222546	2020	JingZhou	1	5.06	2.1
N222546	2020	JingZhou	2	4.7	1.97
N222546	2020	JingZhou	3	4.33	1.91
N222547	2020	JingZhou	1	1.99	1.69
N222547	2020	JingZhou	2	3.57	1.76
N222547	2020	JingZhou	3	3.06	1.75
N222548	2020	JingZhou	1	5.24	2.57
N222548	2020	JingZhou	2	4.98	2.28
N222548	2020	JingZhou	3	5.42	2.32
N222549	2020	JingZhou	1	5.09	2.51
N222549	2020	JingZhou	2	3.47	1.77
N222549	2020	JingZhou	3	4.41	2.32
N222550	2020	JingZhou	1	3.65	2.33
N222550	2020	JingZhou	2	3.98	2.31
N222550	2020	JingZhou	3	4.03	2.45
N222551	2020	JingZhou	1	4.16	2.28
N222551	2020	JingZhou	2	3.99	2.16
N222551	2020	JingZhou	3	3.45	2.08
N222552	2020	JingZhou	1	3.82	2.38
N222552	2020	JingZhou	2	4.99	2.36
N222552	2020	JingZhou	3	4.5	2.16
N222553	2020	JingZhou	1	2.25	2.32
N222553	2020	JingZhou	2	3.65	2.2
N222553	2020	JingZhou	3	2.4	1.92
N222554	2020	JingZhou	1	2.55	1.55
N222554	2020	JingZhou	2	1.91	1.68
N222554	2020	JingZhou	3	2.27	1.44
N222555	2020	JingZhou	1	2.9	1.87
N222555	2020	JingZhou	2	2.56	1.9
N222555	2020	JingZhou	3	2.14	1.65

Line	Year	Location	Replication	SD (mm)	BD (mm)
N222556	2020	JingZhou	1	2.87	1.78
N222556	2020	JingZhou	2	2.23	1.33
N222556	2020	JingZhou	3	3.03	1.49
N222557	2020	JingZhou	1	2.47	1.6
N222557	2020	JingZhou	2	3.47	1.88
N222557	2020	JingZhou	3	3.27	1.87
N222558	2020	JingZhou	1	3.01	1.63
N222558	2020	JingZhou	2	3.41	1.71
N222558	2020	JingZhou	3	3.09	1.81
N222559	2020	JingZhou	1	3.93	2.11
N222559	2020	JingZhou	2	3.75	1.97
N222559	2020	JingZhou	3	3.2	2.02
N222560	2020	JingZhou	1	2.39	1.42
N222560	2020	JingZhou	2	3.27	1.95
N222560	2020	JingZhou	3	3.16	1.56
N222561	2020	JingZhou	1	3.18	1.74
N222561	2020	JingZhou	2	2.67	1.95
N222561	2020	JingZhou	3	2.22	1.52
N222562	2020	JingZhou	1	6.93	2.37
N222562	2020	JingZhou	2	5.66	2.47
N222562	2020	JingZhou	3	4.48	2.32
N222563	2020	JingZhou	1	3.24	1.69
N222563	2020	JingZhou	2	3.56	1.64
N222563	2020	JingZhou	3	1.97	1.76
N222564	2020	JingZhou	1	2.45	1.63
N222564	2020	JingZhou	2	2.91	1.82
N222564	2020	JingZhou	3	2.3	1.6
N222565	2020	JingZhou	1	3.21	2.64
N222565	2020	JingZhou	2	2.5	1.7
N222565	2020	JingZhou	3	2.57	1.69
N222566	2020	JingZhou	1	7.54	2.87
N222566	2020	JingZhou	2	6.26	2.59
N222566	2020	JingZhou	3	7.23	2.72
N222567	2020	JingZhou	1	6.36	2.76
N222567	2020	JingZhou	2	5.25	2.34
N222567	2020	JingZhou	3	4.73	2.29
N222568	2020	JingZhou	1	3.51	2.08
N222568	2020	JingZhou	2	2.96	1.67
N222568	2020	JingZhou	3	2.84	1.99
N222569	2020	JingZhou	1	1.62	1.65
N222569	2020	JingZhou	2	1.69	1.26
N222569	2020	JingZhou	3	1.85	1.21
N222570	2020	JingZhou	1	2.55	1.74
N222570	2020	JingZhou	2	2.58	1.67
N222570	2020	JingZhou	3	2.76	1.76

Line	Year	Location	Replication	SD (mm)	BD (mm)
N222571	2020	JingZhou	1	1.24	1.4
N222571	2020	JingZhou	2	2.12	1.25
N222571	2020	JingZhou	3	-	-
N222572	2020	JingZhou	1	2.91	2
N222572	2020	JingZhou	2	2.58	2
N222572	2020	JingZhou	3	2.85	1.9
N222573	2020	JingZhou	1	1.87	1.19
N222573	2020	JingZhou	2	2.73	1.35
N222573	2020	JingZhou	3	1.76	1.34
N222574	2020	JingZhou	1	4.03	1.91
N222574	2020	JingZhou	2	2.86	1.88
N222574	2020	JingZhou	3	3.65	1.97
N222575	2020	JingZhou	1	4.24	2.49
N222575	2020	JingZhou	2	3.73	2.13
N222575	2020	JingZhou	3	3.15	2.4
N222576	2020	JingZhou	1	4.27	3.41
N222576	2020	JingZhou	2	5.18	2.53
N222576	2020	JingZhou	3	4.46	2.25
N222577	2020	JingZhou	1	3.71	1.53
N222577	2020	JingZhou	2	3.47	1.6
N222577	2020	JingZhou	3	3.42	1.7
N222578	2020	JingZhou	1	4.3	1.88
N222578	2020	JingZhou	2	2.93	1.86
N222578	2020	JingZhou	3	2.96	1.63
N222579	2020	JingZhou	1	8.32	3.04
N222579	2020	JingZhou	2	8.96	3.34
N222579	2020	JingZhou	3	9.56	3.03
N222580	2020	JingZhou	1	4.98	2.56
N222580	2020	JingZhou	2	5.12	2.97
N222580	2020	JingZhou	3	5.67	3.35
N222581	2020	JingZhou	1	3.74	2.2
N222581	2020	JingZhou	2	3.17	2.43
N222581	2020	JingZhou	3	4.62	2.55
N222582	2020	JingZhou	1	5.16	2.22
N222582	2020	JingZhou	2	3.42	2.4
N222582	2020	JingZhou	3	4.93	2.3
N222583	2020	JingZhou	1	1.9	1.49
N222583	2020	JingZhou	2	3.87	2.34
N222583	2020	JingZhou	3	5.22	2.57
N222584	2020	JingZhou	1	2.43	1.96
N222584	2020	JingZhou	2	3.62	2.19
N222584	2020	JingZhou	3	2.58	2
N222585	2020	JingZhou	1	6.11	2.7
N222585	2020	JingZhou	2	8.24	2.81
N222585	2020	JingZhou	3	5.81	2.75

Line	Year	Location	Replication	SD (mm)	BD (mm)
N222586	2020	JingZhou	1	4.51	2.04
N222586	2020	JingZhou	2	4.01	2.33
N222586	2020	JingZhou	3	5.94	1.95
N222587	2020	JingZhou	1	3.84	2.17
N222587	2020	JingZhou	2	3.92	2.48
N222587	2020	JingZhou	3	4.46	2.73
N222588	2020	JingZhou	1	3.59	1.89
N222588	2020	JingZhou	2	3.13	1.67
N222588	2020	JingZhou	3	2.41	1.7
N222589	2020	JingZhou	1	3.74	1.85
N222589	2020	JingZhou	2	5.1	1.93
N222589	2020	JingZhou	3	2.89	1.89
N222590	2020	JingZhou	1	5.14	2.32
N222590	2020	JingZhou	2	5.18	2.19
N222590	2020	JingZhou	3	6.22	2.36
N222591	2020	JingZhou	1	2.56	1.9
N222591	2020	JingZhou	2	2.54	2
N222591	2020	JingZhou	3	2.94	1.84
N222592	2020	JingZhou	1	5.5	2.29
N222592	2020	JingZhou	2	4.57	2.13
N222592	2020	JingZhou	3	3.67	1.93
N222593	2020	JingZhou	1	3.33	1.9
N222593	2020	JingZhou	2	3.47	1.93
N222593	2020	JingZhou	3	3.61	2.05
N222594	2020	JingZhou	1	2.86	1.88
N222594	2020	JingZhou	2	3.06	2.22
N222594	2020	JingZhou	3	2.83	1.92
N222595	2020	JingZhou	1	3.94	1.78
N222595	2020	JingZhou	2	1.57	0.47
N222595	2020	JingZhou	3	3.68	2.09
N222596	2020	JingZhou	1	2.09	1.51
N222596	2020	JingZhou	2	2.21	1.64
N222596	2020	JingZhou	3	1.77	1.22
N222597	2020	JingZhou	1	7.46	3.12
N222597	2020	JingZhou	2	7.62	3.17
N222597	2020	JingZhou	3	6.12	2.89
N222598	2020	JingZhou	1	2.56	2.02
N222598	2020	JingZhou	2	2	1.6
N222598	2020	JingZhou	3	2.01	2.23
N222599	2020	JingZhou	1	3.83	2.19
N222599	2020	JingZhou	2	3.14	2.42
N222599	2020	JingZhou	3	3.09	2.03
N222600	2020	JingZhou	1	2.86	2.24
N222600	2020	JingZhou	2	2.42	1.68
N222600	2020	JingZhou	3	3.09	2.09

Line	Year	Location	Replication	SD (mm)	BD (mm)
N222601	2020	JingZhou	1	2.71	1.6
N222601	2020	JingZhou	2	3.58	1.68
N222601	2020	JingZhou	3	3.01	1.58
N222602	2020	JingZhou	1	4.91	2.5
N222602	2020	JingZhou	2	4.37	2.17
N222602	2020	JingZhou	3	4.32	2.43
N222603	2020	JingZhou	1	4.01	2.93
N222603	2020	JingZhou	2	4.5	2.59
N222603	2020	JingZhou	3	6.66	2.65
N222604	2020	JingZhou	1	3.51	2.25
N222604	2020	JingZhou	2	4.04	2.6
N222604	2020	JingZhou	3	4.01	2.52
N222605	2020	JingZhou	1	5.3	2.88
N222605	2020	JingZhou	2	4.88	2.29
N222605	2020	JingZhou	3	6.37	2.59
N222606	2020	JingZhou	1	4.15	2.04
N222606	2020	JingZhou	2	3.13	1.87
N222606	2020	JingZhou	3	3.35	1.78
N222607	2020	JingZhou	1	5.07	2.26
N222607	2020	JingZhou	2	4.03	2.33
N222607	2020	JingZhou	3	5.18	2.26
N222608	2020	JingZhou	1	3.39	2.11
N222608	2020	JingZhou	2	2.93	1.98
N222608	2020	JingZhou	3	3.26	2.15
N222609	2020	JingZhou	1	3.35	1.76
N222609	2020	JingZhou	2	2.16	1.54
N222609	2020	JingZhou	3	4.67	1.68
N222610	2020	JingZhou	1	3.98	2.61
N222610	2020	JingZhou	2	3.36	2.16
N222610	2020	JingZhou	3	2.81	2.18
N222611	2020	JingZhou	1	3.57	2.19
N222611	2020	JingZhou	2	4.66	2.25
N222611	2020	JingZhou	3	2.9	2.19
N222612	2020	JingZhou	1	4.76	2.16
N222612	2020	JingZhou	2	-	-
N222612	2020	JingZhou	3	3.48	1.98
N222613	2020	JingZhou	1	3.88	1.95
N222613	2020	JingZhou	2	4.37	1.94
N222613	2020	JingZhou	3	2.29	1.68
N222614	2020	JingZhou	1	6.19	2.28
N222614	2020	JingZhou	2	3.32	2.2
N222614	2020	JingZhou	3	2.63	2.35
N222615	2020	JingZhou	1	3.73	2.16
N222615	2020	JingZhou	2	3.53	2.15
N222615	2020	JingZhou	3	3.35	2.08

Line	Year	Location	Replication	SD (mm)	BD (mm)
N222616	2020	JingZhou	1	5.96	3.13
N222616	2020	JingZhou	2	6.82	3.28
N222616	2020	JingZhou	3	7.07	2.76
N222617	2020	JingZhou	1	3.09	2.31
N222617	2020	JingZhou	2	3.22	2.32
N222617	2020	JingZhou	3	3.3	2.21
N222618	2020	JingZhou	1	3.87	1.82
N222618	2020	JingZhou	2	5.21	1.99
N222618	2020	JingZhou	3	4.69	1.89
N222619	2020	JingZhou	1	2.24	1.5
N222619	2020	JingZhou	2	2.91	1.42
N222619	2020	JingZhou	3	2.88	1.61
N222620	2020	JingZhou	1	2.11	1.02
N222620	2020	JingZhou	2	3.03	1.63
N222620	2020	JingZhou	3	2.38	1.34
N222621	2020	JingZhou	1	2.75	1.77
N222621	2020	JingZhou	2	4.46	2.74
N222621	2020	JingZhou	3	2.57	1.58
N222622	2020	JingZhou	1	5.45	2.14
N222622	2020	JingZhou	2	4.35	1.96
N222622	2020	JingZhou	3	4.98	2.11
N222623	2020	JingZhou	1	1.86	1.76
N222623	2020	JingZhou	2	3.48	1.69
N222623	2020	JingZhou	3	3.75	1.86
N222624	2020	JingZhou	1	3.64	2.03
N222624	2020	JingZhou	2	5.23	2.12
N222624	2020	JingZhou	3	5.32	1.89
N222625	2020	JingZhou	1	4.06	2.5
N222625	2020	JingZhou	2	3.36	2.07
N222625	2020	JingZhou	3	3.36	1.97
N222628	2020	JingZhou	1	5.25	2.25
N222628	2020	JingZhou	2	6.05	2.97
N222628	2020	JingZhou	3	3.62	2.61
N222629	2020	JingZhou	1	2.3	1.89
N222629	2020	JingZhou	2	2.62	1.6
N222629	2020	JingZhou	3	2.22	1.8
N222630	2020	JingZhou	1	4.64	2.47
N222630	2020	JingZhou	2	3.83	2.35
N222630	2020	JingZhou	3	2.64	2.37
N222631	2020	JingZhou	1	3.54	2.07
N222631	2020	JingZhou	2	4.47	2.17
N222631	2020	JingZhou	3	4.69	2.22
N222632	2020	JingZhou	1	3.98	2.6
N222632	2020	JingZhou	2	4.12	2.96
N222632	2020	JingZhou	3	4.02	2.42

Line	Year	Location	Replication	SD (mm)	BD (mm)
N222633	2020	JingZhou	1	2.96	2.44
N222633	2020	JingZhou	2	4.17	2.38
N222633	2020	JingZhou	3	2.64	1.9
N222634	2020	JingZhou	1	2.86	1.64
N222634	2020	JingZhou	2	2.45	1.68
N222634	2020	JingZhou	3	3.19	1.76
N222635	2020	JingZhou	1	1.35	1.08
N222635	2020	JingZhou	2	1.79	1.14
N222635	2020	JingZhou	3	1.67	1.45
N222636	2020	JingZhou	1	2.49	1.22
N222636	2020	JingZhou	2	2.14	1.61
N222636	2020	JingZhou	3	2.03	1.21
N222637	2020	JingZhou	1	3.02	1.92
N222637	2020	JingZhou	2	2.25	1.49
N222637	2020	JingZhou	3	2.97	1.86
N222638	2020	JingZhou	1	1.85	1.95
N222638	2020	JingZhou	2	-	-
N222638	2020	JingZhou	3	-	-
N222639	2020	JingZhou	1	2.83	2.28
N222639	2020	JingZhou	2	3.48	2.16
N222639	2020	JingZhou	3	2.81	2.03
N222640	2020	JingZhou	1	2.74	1.8
N222640	2020	JingZhou	2	4.25	1.79
N222640	2020	JingZhou	3	3.11	1.92
N222641	2020	JingZhou	1	2.61	1.58
N222641	2020	JingZhou	2	3.51	1.44
N222641	2020	JingZhou	3	2.17	1.33
N222642	2020	JingZhou	1	2.96	2.35
N222642	2020	JingZhou	2	4.43	2.14
N222642	2020	JingZhou	3	3.9	2.25
N222643	2020	JingZhou	1	3.71	1.91
N222643	2020	JingZhou	2	4.35	2.33
N222643	2020	JingZhou	3	4.69	2.23
N222644	2020	JingZhou	1	2.35	1.76
N222644	2020	JingZhou	2	3.63	1.93
N222644	2020	JingZhou	3	2.89	2.09
N222645	2020	JingZhou	1	2.55	1.57
N222645	2020	JingZhou	2	4.47	1.98
N222645	2020	JingZhou	3	3.14	1.91
N222646	2020	JingZhou	1	7.2	2.88
N222646	2020	JingZhou	2	5.08	2.43
N222646	2020	JingZhou	3	8.21	2.63
N222647	2020	JingZhou	1	2.59	1.46
N222647	2020	JingZhou	2	3.93	1.68
N222647	2020	JingZhou	3	2.45	1.44

Line	Year	Location	Replication	SD (mm)	BD (mm)
N222648	2020	JingZhou	1	2.84	1.77
N222648	2020	JingZhou	2	2.24	1.45
N222648	2020	JingZhou	3	2.74	1.54
N222649	2020	JingZhou	1	2.87	2.24
N222649	2020	JingZhou	2	3.7	2.05
N222649	2020	JingZhou	3	3.18	1.99
N222650	2020	JingZhou	1	2.71	1.62
N222650	2020	JingZhou	2	2.97	1.81
N222650	2020	JingZhou	3	2.94	1.88
N222651	2020	JingZhou	1	4.03	2.33
N222651	2020	JingZhou	2	-	-
N222651	2020	JingZhou	3	4.2	2.75
N222652	2020	JingZhou	1	2.4	1.78
N222652	2020	JingZhou	2	2.59	1.89
N222652	2020	JingZhou	3	2.23	1.73
N222653	2020	JingZhou	1	1.49	1.3
N222653	2020	JingZhou	2	1.73	1.33
N222653	2020	JingZhou	3	1.24	0.85
N222654	2020	JingZhou	1	2.58	1.67
N222654	2020	JingZhou	2	2.06	1.33
N222654	2020	JingZhou	3	2.74	1.53
N222655	2020	JingZhou	1	5.12	2.31
N222655	2020	JingZhou	2	4.78	2.33
N222655	2020	JingZhou	3	4.24	2.5
N222656	2020	JingZhou	1	2.85	1.74
N222656	2020	JingZhou	2	3.09	1.77
N222656	2020	JingZhou	3	2.51	1.7
N222657	2020	JingZhou	1	4.92	2.18
N222657	2020	JingZhou	2	3.57	2.16
N222657	2020	JingZhou	3	3.16	2.15
N222658	2020	JingZhou	1	6.77	2.92
N222658	2020	JingZhou	2	6.52	2.74
N222658	2020	JingZhou	3	6.64	2.6
N222659	2020	JingZhou	1	4.68	2.29
N222659	2020	JingZhou	2	5.52	2.62
N222659	2020	JingZhou	3	4.58	2.39
N222660	2020	JingZhou	1	3.49	2.04
N222660	2020	JingZhou	2	3.84	2.88
N222660	2020	JingZhou	3	5.23	2.22
N222661	2020	JingZhou	1	7.04	2.63
N222661	2020	JingZhou	2	4.84	2.9
N222661	2020	JingZhou	3	6.06	2.31
N222662	2020	JingZhou	1	3.8	2.13
N222662	2020	JingZhou	2	5.29	2.34
N222662	2020	JingZhou	3	5	2.87

Line	Year	Location	Replication	SD (mm)	BD (mm)
N222663	2020	JingZhou	1	3.67	1.63
N222663	2020	JingZhou	2	3.12	1.79
N222663	2020	JingZhou	3	4.89	1.73
N222664	2020	JingZhou	1	1.75	1.54
N222664	2020	JingZhou	2	1.56	1.27
N222664	2020	JingZhou	3	1.58	1.22
N222665	2020	JingZhou	1	3.78	1.97
N222665	2020	JingZhou	2	2.8	1.91
N222665	2020	JingZhou	3	4.87	2.11
N222666	2020	JingZhou	1	4.07	2.54
N222666	2020	JingZhou	2	4.98	2.45
N222666	2020	JingZhou	3	4.26	2.68
N222667	2020	JingZhou	1	4.32	2.35
N222667	2020	JingZhou	2	4.34	2.2
N222667	2020	JingZhou	3	4.17	2.03
N222668	2020	JingZhou	1	2.16	1.43
N222668	2020	JingZhou	2	1.85	1.72
N222668	2020	JingZhou	3	2.34	1.84
N222669	2020	JingZhou	1	2.93	1.96
N222669	2020	JingZhou	2	4.84	2.41
N222669	2020	JingZhou	3	2.35	1.58
N222670	2020	JingZhou	1	3.64	1.99
N222670	2020	JingZhou	2	3.31	2.12
N222670	2020	JingZhou	3	3.81	1.7
N222671	2020	JingZhou	1	4.76	2
N222671	2020	JingZhou	2	3.1	2.06
N222671	2020	JingZhou	3	4.76	2.02
N222672	2020	JingZhou	1	2.04	1.87
N222672	2020	JingZhou	2	2.23	1.8
N222672	2020	JingZhou	3	3.26	2.01
N222673	2020	JingZhou	1	1.92	1.59
N222673	2020	JingZhou	2	2.49	1.89
N222673	2020	JingZhou	3	2.19	2.12
N222674	2020	JingZhou	1	5.96	2.42
N222674	2020	JingZhou	2	6.88	2.85
N222674	2020	JingZhou	3	6.27	2.76
N222675	2020	JingZhou	1	6.66	2.21
N222675	2020	JingZhou	2	4.68	2.02
N222675	2020	JingZhou	3	3.46	1.9
N222676	2020	JingZhou	1	5.08	2.3
N222676	2020	JingZhou	2	6.12	3.08
N222676	2020	JingZhou	3	5.48	3.19
N222677	2020	JingZhou	1	2.67	1.81
N222677	2020	JingZhou	2	2.58	2.18
N222677	2020	JingZhou	3	2.54	3.18

Line	Year	Location	Replication	SD (mm)	BD (mm)
N222678	2020	JingZhou	1	2.96	1.81
N222678	2020	JingZhou	2	2.39	1.69
N222678	2020	JingZhou	3	3.34	1.74
N222679	2020	JingZhou	1	3.99	2.8
N222679	2020	JingZhou	2	5.95	2.98
N222679	2020	JingZhou	3	4.49	2.41
N222680	2020	JingZhou	1	4.78	2.33
N222680	2020	JingZhou	2	5.54	2.46
N222680	2020	JingZhou	3	6.3	2.51
N222681	2020	JingZhou	1	6.7	2.84
N222681	2020	JingZhou	2	5.81	3.25
N222681	2020	JingZhou	3	5.29	3
N222682	2020	JingZhou	1	6.94	2.84
N222682	2020	JingZhou	2	3.97	2.89
N222682	2020	JingZhou	3	5.4	2.22
N222683	2020	JingZhou	1	7.61	2.76
N222683	2020	JingZhou	2	7.39	2.66
N222683	2020	JingZhou	3	-	-
N222684	2020	JingZhou	1	4.83	1.91
N222684	2020	JingZhou	2	5.49	2.13
N222684	2020	JingZhou	3	5.4	2.05
N222685	2020	JingZhou	1	7.49	3.14
N222685	2020	JingZhou	2	9.87	4.19
N222685	2020	JingZhou	3	8.91	3.04
N222686	2020	JingZhou	1	5.54	2.54
N222686	2020	JingZhou	2	6.46	2.42
N222686	2020	JingZhou	3	4.76	2.21
N222687	2020	JingZhou	1	3.49	1.75
N222687	2020	JingZhou	2	-	-
N222687	2020	JingZhou	3	3.41	1.74
N222688	2020	JingZhou	1	1.64	1.13
N222688	2020	JingZhou	2	-	-
N222688	2020	JingZhou	3	-	-
N222689	2020	JingZhou	1	3.59	1.81
N222689	2020	JingZhou	2	1.5	1.73
N222689	2020	JingZhou	3	2.92	1.59
N222690	2020	JingZhou	1	4.85	2.91
N222690	2020	JingZhou	2	6.53	3.12
N222690	2020	JingZhou	3	5.29	2.72
N222691	2020	JingZhou	1	3.52	2.28
N222691	2020	JingZhou	2	2.08	2.14
N222691	2020	JingZhou	3	2.36	1.85
N222692	2020	JingZhou	1	3.86	1.93
N222692	2020	JingZhou	2	2.28	1.83
N222692	2020	JingZhou	3	2.49	1.69

Line	Year	Location	Replication	SD (mm)	BD (mm)
N222693	2020	JingZhou	1	2.86	1.69
N222693	2020	JingZhou	2	2.62	1.62
N222693	2020	JingZhou	3	2.44	1.57
N222694	2020	JingZhou	1	3.36	1.95
N222694	2020	JingZhou	2	3.72	2.11
N222694	2020	JingZhou	3	3.31	1.85
N222695	2020	JingZhou	1	5.18	1.86
N222695	2020	JingZhou	2	3.14	2
N222695	2020	JingZhou	3	3.15	2.01
N222696	2020	JingZhou	1	2.22	1.09
N222696	2020	JingZhou	2	2.91	2
N222696	2020	JingZhou	3	2.43	1.49
N222697	2020	JingZhou	1	3.34	1.9
N222697	2020	JingZhou	2	2.69	1.53
N222697	2020	JingZhou	3	1.58	1.52
N222698	2020	JingZhou	1	2.73	1.56
N222698	2020	JingZhou	2	4.9	1.58
N222698	2020	JingZhou	3	2.89	1.43
N222699	2020	JingZhou	1	2.46	1.52
N222699	2020	JingZhou	2	2.53	1.81
N222699	2020	JingZhou	3	2.12	1.21
N222700	2020	JingZhou	1	4.43	2.64
N222700	2020	JingZhou	2	5.3	2.48
N222700	2020	JingZhou	3	4.61	2.86
N222701	2020	JingZhou	1	4.22	2.88
N222701	2020	JingZhou	2	7	2.65
N222701	2020	JingZhou	3	6.21	2.75
N222702	2020	JingZhou	1	3.95	1.95
N222702	2020	JingZhou	2	3.92	2.03
N222702	2020	JingZhou	3	2.54	1.8
N222703	2020	JingZhou	1	1.58	2.04
N222703	2020	JingZhou	2	2.63	2.45
N222703	2020	JingZhou	3	2.46	2.3
N222704	2020	JingZhou	1	6.5	2.33
N222704	2020	JingZhou	2	3.14	2.16
N222704	2020	JingZhou	3	3.07	1.75
N222705	2020	JingZhou	1	2.12	1.43
N222705	2020	JingZhou	2	2.32	1.96
N222705	2020	JingZhou	3	1.82	1.68
N222706	2020	JingZhou	1	2.41	1.88
N222706	2020	JingZhou	2	3.4	2.13
N222706	2020	JingZhou	3	2.46	1.74
N222707	2020	JingZhou	1	3.2	1.9
N222707	2020	JingZhou	2	1.68	2.18
N222707	2020	JingZhou	3	3.11	1.82

Line	Year	Location	Replication	SD (mm)	BD (mm)
N222708	2020	JingZhou	1	1.9	1.47
N222708	2020	JingZhou	2	2.15	1.35
N222708	2020	JingZhou	3	2.36	1.35
N222709	2020	JingZhou	1	3.16	2.31
N222709	2020	JingZhou	2	3.67	2.39
N222709	2020	JingZhou	3	3.46	1.8
N222710	2020	JingZhou	1	1.6	1.85
N222710	2020	JingZhou	2	2.43	1.63
N222710	2020	JingZhou	3	1.89	1.79
N222711	2020	JingZhou	1	3.33	1.7
N222711	2020	JingZhou	2	2.62	1.71
N222711	2020	JingZhou	3	2.11	1.63
N222712	2020	JingZhou	1	2.11	2.02
N222712	2020	JingZhou	2	2.78	1.92
N222712	2020	JingZhou	3	2.85	1.71
N222713	2020	JingZhou	1	3.36	1.76
N222713	2020	JingZhou	2	5.68	1.76
N222713	2020	JingZhou	3	3.01	1.45
N222715	2020	JingZhou	1	5.18	2.25
N222715	2020	JingZhou	2	4.56	2.17
N222715	2020	JingZhou	3	4.42	2.16
N222716	2020	JingZhou	1	3.55	1.93
N222716	2020	JingZhou	2	3.88	2.23
N222716	2020	JingZhou	3	3.39	2.05
N222717	2020	JingZhou	1	5.32	2.57
N222717	2020	JingZhou	2	7.16	2.53
N222717	2020	JingZhou	3	5.23	2.57
N222718	2020	JingZhou	1	4.19	2.67
N222718	2020	JingZhou	2	6.71	2.67
N222718	2020	JingZhou	3	6.46	2.97
N222719	2020	JingZhou	1	3.35	2.2
N222719	2020	JingZhou	2	3.06	1.93
N222719	2020	JingZhou	3	3.98	1.9
N222720	2020	JingZhou	1	5.27	2.56
N222720	2020	JingZhou	2	6.2	2.4
N222720	2020	JingZhou	3	5.88	2.55
N222721	2020	JingZhou	1	1.65	0.99
N222721	2020	JingZhou	2	1.17	0.85
N222721	2020	JingZhou	3	1.64	1.36
N222722	2020	JingZhou	1	4.22	2.01
N222722	2020	JingZhou	2	2.58	1.79
N222722	2020	JingZhou	3	4.05	1.71
N222723	2020	JingZhou	1	1.72	1.37
N222723	2020	JingZhou	2	1.63	1.45
N222723	2020	JingZhou	3	1.81	1.49

Line	Year	Location	Replication	SD (mm)	BD (mm)
N222724	2020	JingZhou	1	4.09	1.93
N222724	2020	JingZhou	2	2.02	1.44
N222724	2020	JingZhou	3	2.34	1.55
N222725	2020	JingZhou	1	2.63	1.59
N222725	2020	JingZhou	2	1.94	1.39
N222725	2020	JingZhou	3	1.87	1.29
N222726	2020	JingZhou	1	2.45	1.74
N222726	2020	JingZhou	2	2.3	1.29
N222726	2020	JingZhou	3	2.48	1.72
N222727	2020	JingZhou	1	1.14	1.85
N222727	2020	JingZhou	2	1.28	1.07
N222727	2020	JingZhou	3	1.31	1.1
N222728	2020	JingZhou	1	4.14	2.13
N222728	2020	JingZhou	2	2.82	2.01
N222728	2020	JingZhou	3	2.99	1.95
N222729	2020	JingZhou	1	4.99	2.69
N222729	2020	JingZhou	2	3.85	2.19
N222729	2020	JingZhou	3	2.81	2.04
N222730	2020	JingZhou	1	3.48	2.17
N222730	2020	JingZhou	2	2.91	1.84
N222730	2020	JingZhou	3	3.78	1.61
N222731	2020	JingZhou	1	1.72	1.51
N222731	2020	JingZhou	2	1.93	1.98
N222731	2020	JingZhou	3	4.81	2.24
N222732	2020	JingZhou	1	4.75	2.32
N222732	2020	JingZhou	2	6.09	2.83
N222732	2020	JingZhou	3	4.49	2.33
N222733	2020	JingZhou	1	5.05	2.57
N222733	2020	JingZhou	2	5.35	3.11
N222733	2020	JingZhou	3	5.39	2.59
N222734	2020	JingZhou	1	7.02	2.96
N222734	2020	JingZhou	2	5.97	3.22
N222734	2020	JingZhou	3	5.93	2.77
N222735	2020	JingZhou	1	2.21	2.13
N222735	2020	JingZhou	2	3.35	2.12
N222735	2020	JingZhou	3	2.07	2.07
N222736	2020	JingZhou	1	4.68	1.99
N222736	2020	JingZhou	2	3.08	1.79
N222736	2020	JingZhou	3	3.29	1.76
N222737	2020	JingZhou	1	4.29	2.27
N222737	2020	JingZhou	2	3.1	1.89
N222737	2020	JingZhou	3	2.81	2.89
N222738	2020	JingZhou	1	2.17	1.36
N222738	2020	JingZhou	2	1.75	1.3
N222738	2020	JingZhou	3	1.44	1.54

Line	Year	Location	Replication	SD (mm)	BD (mm)
N222739	2020	JingZhou	1	2.48	1.66
N222739	2020	JingZhou	2	2.51	1.77
N222739	2020	JingZhou	3	2.14	1.64
N222740	2020	JingZhou	1	5.59	2.68
N222740	2020	JingZhou	2	5.16	2.18
N222740	2020	JingZhou	3	4.2	2.22
N222741	2020	JingZhou	1	3.3	1.9
N222741	2020	JingZhou	2	4.36	2.24
N222741	2020	JingZhou	3	4.62	2.1
N222742	2020	JingZhou	1	1.48	1.17
N222742	2020	JingZhou	2	2.19	1.59
N222742	2020	JingZhou	3	1.11	1.67
N222743	2020	JingZhou	1	4.4	1.8
N222743	2020	JingZhou	2	3.8	2.12
N222743	2020	JingZhou	3	3.87	1.95
N222744	2020	JingZhou	1	2.9	1.96
N222744	2020	JingZhou	2	3.68	1.78
N222744	2020	JingZhou	3	1.93	1.51
N222745	2020	JingZhou	1	4.86	2.41
N222745	2020	JingZhou	2	6.22	2.45
N222745	2020	JingZhou	3	5.91	2.35
N222746	2020	JingZhou	1	1.87	1.63
N222746	2020	JingZhou	2	2	1.7
N222746	2020	JingZhou	3	2.38	2.11
N222747	2020	JingZhou	1	2.64	2.18
N222747	2020	JingZhou	2	2.25	1.76
N222747	2020	JingZhou	3	2.67	1.94
N222748	2020	JingZhou	1	4.12	1.97
N222748	2020	JingZhou	2	3.39	2.04
N222748	2020	JingZhou	3	4.48	2.07
N222749	2020	JingZhou	1	2.28	1.28
N222749	2020	JingZhou	2	2	1.44
N222749	2020	JingZhou	3	2.76	1.73
N222750	2020	JingZhou	1	2.16	1.59
N222750	2020	JingZhou	2	1.74	1.31
N222750	2020	JingZhou	3	1.82	1.66
N222751	2020	JingZhou	1	3.24	2.05
N222751	2020	JingZhou	2	3.61	2.32
N222751	2020	JingZhou	3	3.58	2.49
N222752	2020	JingZhou	1	3.63	2.29
N222752	2020	JingZhou	2	3.49	2.04
N222752	2020	JingZhou	3	2.64	1.97
N222753	2020	JingZhou	1	4.96	2.21
N222753	2020	JingZhou	2	5.67	2.63
N222753	2020	JingZhou	3	4.79	2.23

Line	Year	Location	Replication	SD (mm)	BD (mm)
N222754	2020	JingZhou	1	2.47	1.76
N222754	2020	JingZhou	2	3.56	2.28
N222754	2020	JingZhou	3	3.11	2.34
N222755	2020	JingZhou	1	5.26	2.36
N222755	2020	JingZhou	2	4.71	2.38
N222755	2020	JingZhou	3	6.36	2.56
N222756	2020	JingZhou	1	5.92	2.63
N222756	2020	JingZhou	2	5.64	2.61
N222756	2020	JingZhou	3	3.11	2.19
N222757	2020	JingZhou	1	6.2	2.56
N222757	2020	JingZhou	2	6	2.61
N222757	2020	JingZhou	3	8.38	2.74
N222759	2020	JingZhou	1	5.06	1.96
N222759	2020	JingZhou	2	4.59	1.94
N222759	2020	JingZhou	3	4.14	1.7
N222760	2020	JingZhou	1	3.88	2.05
N222760	2020	JingZhou	2	5.23	2.29
N222760	2020	JingZhou	3	-	-
N222761	2020	JingZhou	1	3.69	2.08
N222761	2020	JingZhou	2	3.82	2.2
N222761	2020	JingZhou	3	3.37	2.11
N222762	2020	JingZhou	1	3.32	2.11
N222762	2020	JingZhou	2	4.34	2.2
N222762	2020	JingZhou	3	3.45	1.96
N222763	2020	JingZhou	1	2.16	2.4
N222763	2020	JingZhou	2	1.99	1.64
N222763	2020	JingZhou	3	2.25	1.52
N222764	2020	JingZhou	1	3.79	2.01
N222764	2020	JingZhou	2	4.5	2.17
N222764	2020	JingZhou	3	5.69	2.34
N222765	2020	JingZhou	1	2.12	1.75
N222765	2020	JingZhou	2	2.01	1.53
N222765	2020	JingZhou	3	2.61	1.56
N222766	2020	JingZhou	1	4.54	2.42
N222766	2020	JingZhou	2	5.84	2.23
N222766	2020	JingZhou	3	3.18	1.78
N222767	2020	JingZhou	1	4.24	2.52
N222767	2020	JingZhou	2	4.87	2.11
N222767	2020	JingZhou	3	3.78	2.33
N222768	2020	JingZhou	1	2.39	1.54
N222768	2020	JingZhou	2	2.53	1.51
N222768	2020	JingZhou	3	2.34	1.54
N222769	2020	JingZhou	1	2.59	1.53
N222769	2020	JingZhou	2	2.54	1.58
N222769	2020	JingZhou	3	1.88	1.34

Line	Year	Location	Replication	SD (mm)	BD (mm)
N222770	2020	JingZhou	1	1.48	1.49
N222770	2020	JingZhou	2	2.49	2.81
N222770	2020	JingZhou	3	2.89	1.3
N222771	2020	JingZhou	1	2.98	1.96
N222771	2020	JingZhou	2	3.02	2.07
N222771	2020	JingZhou	3	2.92	1.77
N222772	2020	JingZhou	1	4.37	2.32
N222772	2020	JingZhou	2	4.1	2.53
N222772	2020	JingZhou	3	5.25	2.31
N222773	2020	JingZhou	1	2.78	1.74
N222773	2020	JingZhou	2	3.15	1.75
N222773	2020	JingZhou	3	2.97	1.96
N222774	2020	JingZhou	1	4	1.96
N222774	2020	JingZhou	2	4.02	1.92
N222774	2020	JingZhou	3	5.05	2.21
N222775	2020	JingZhou	1	-	-
N222775	2020	JingZhou	2	1.17	-
N222775	2020	JingZhou	3	1.78	1.03
N222776	2020	JingZhou	1	2.92	1.99
N222776	2020	JingZhou	2	1.69	2.19
N222776	2020	JingZhou	3	2.21	1.82
N222777	2020	JingZhou	1	1.78	1.35
N222777	2020	JingZhou	2	1.98	1.21
N222777	2020	JingZhou	3	0.9	1.14
N222778	2020	JingZhou	1	2.08	1.48
N222778	2020	JingZhou	2	1.78	1.25
N222778	2020	JingZhou	3	2.35	1.23
N222779	2020	JingZhou	1	2.76	1.88
N222779	2020	JingZhou	2	2.66	1.73
N222779	2020	JingZhou	3	2.68	1.35
N222780	2020	JingZhou	1	5.31	2.42
N222780	2020	JingZhou	2	4.19	2.65
N222780	2020	JingZhou	3	6.85	2.69
N222781	2020	JingZhou	1	5.56	2
N222781	2020	JingZhou	2	3.21	1.83
N222781	2020	JingZhou	3	3.21	1.83
N222782	2020	JingZhou	1	3.83	1.79
N222782	2020	JingZhou	2	4.66	2.13
N222782	2020	JingZhou	3	3.13	1.78
N222783	2020	JingZhou	1	-	-
N222783	2020	JingZhou	2	-	-
N222783	2020	JingZhou	3	-	-
N222784	2020	JingZhou	1	5.43	2.14
N222784	2020	JingZhou	2	3.53	1.81
N222784	2020	JingZhou	3	4.7	2.08

Line	Year	Location	Replication	SD (mm)	BD (mm)
N222785	2020	JingZhou	1	2.69	2.01
N222785	2020	JingZhou	2	3.82	1.97
N222785	2020	JingZhou	3	4.07	2.07
N222786	2020	JingZhou	1	4.24	1.67
N222786	2020	JingZhou	2	4.69	1.84
N222786	2020	JingZhou	3	5.12	1.91
N222787	2020	JingZhou	1	3.88	1.79
N222787	2020	JingZhou	2	2.24	1.85
N222787	2020	JingZhou	3	2.07	1.79
N222788	2020	JingZhou	1	2.28	1.65
N222788	2020	JingZhou	2	2.95	1.85
N222788	2020	JingZhou	3	2.27	1.74
N222789	2020	JingZhou	1	4.4	2.71
N222789	2020	JingZhou	2	4.82	2.27
N222789	2020	JingZhou	3	3.76	2.32
N222790	2020	JingZhou	1	2.54	2.02
N222790	2020	JingZhou	2	2.38	2.4
N222790	2020	JingZhou	3	1.99	1.43
N222791	2020	JingZhou	1	2.95	1.92
N222791	2020	JingZhou	2	3.18	2.39
N222791	2020	JingZhou	3	2.57	1.92
N222792	2020	JingZhou	1	2.78	1.54
N222792	2020	JingZhou	2	2.05	1.68
N222792	2020	JingZhou	3	2.59	1.39
N222793	2020	JingZhou	1	5.86	2.14
N222793	2020	JingZhou	2	4.35	2.33
N222793	2020	JingZhou	3	4.19	2.2
N222794	2020	JingZhou	1	3	1.79
N222794	2020	JingZhou	2	3.57	1.54
N222794	2020	JingZhou	3	2.26	1.82
N222795	2020	JingZhou	1	2.91	2.11
N222795	2020	JingZhou	2	2.49	2.1
N222795	2020	JingZhou	3	3.81	2.3
N222796	2020	JingZhou	1	1.86	1.42
N222796	2020	JingZhou	2	1.72	1.35
N222796	2020	JingZhou	3	2.01	1.34
N222797	2020	JingZhou	1	3.57	2.2
N222797	2020	JingZhou	2	3.71	1.8
N222797	2020	JingZhou	3	4.07	2.2
N222798	2020	JingZhou	1	3.3	2.54
N222798	2020	JingZhou	2	3.86	2.45
N222798	2020	JingZhou	3	3.32	2.37
N222799	2020	JingZhou	1	4.84	2.23
N222799	2020	JingZhou	2	3.17	2.08
N222799	2020	JingZhou	3	2.66	2.03

Line	Year	Location	Replication	SD (mm)	BD (mm)
N222800	2020	JingZhou	1	3.15	1.55
N222800	2020	JingZhou	2	2.65	1.58
N222800	2020	JingZhou	3	2.13	1.54
N222801	2020	JingZhou	1	6.32	2.83
N222801	2020	JingZhou	2	5.27	2.8
N222801	2020	JingZhou	3	6.21	2.73
N222802	2020	JingZhou	1	2.46	1.77
N222802	2020	JingZhou	2	2.19	1.7
N222802	2020	JingZhou	3	1.65	1.34
N222803	2020	JingZhou	1	1.59	1.28
N222803	2020	JingZhou	2	2.37	1.52
N222803	2020	JingZhou	3	1.53	1.14
N222804	2020	JingZhou	1	2.36	1.7
N222804	2020	JingZhou	2	2.96	1.53
N222804	2020	JingZhou	3	2.57	1.4
N222805	2020	JingZhou	1	3.62	2.34
N222805	2020	JingZhou	2	3.26	1.91
N222805	2020	JingZhou	3	5.35	2.2
N222806	2020	JingZhou	1	5.36	2.34
N222806	2020	JingZhou	2	4.75	2.44
N222806	2020	JingZhou	3	4.74	2.12
N222807	2020	JingZhou	1	4.63	1.82
N222807	2020	JingZhou	2	3.78	2.03
N222807	2020	JingZhou	3	4.9	2.06
N222808	2020	JingZhou	1	6.86	3.22
N222808	2020	JingZhou	2	6.52	2.81
N222808	2020	JingZhou	3	5.45	2.86
N222809	2020	JingZhou	1	3.57	1.7
N222809	2020	JingZhou	2	1.7	1.55
N222809	2020	JingZhou	3	3.15	1.95
N222810	2020	JingZhou	1	4.05	2.09
N222810	2020	JingZhou	2	3.59	2.08
N222810	2020	JingZhou	3	3.22	2.25
N222811	2020	JingZhou	1	1.91	1.64
N222811	2020	JingZhou	2	1.85	1.45
N222811	2020	JingZhou	3	1.99	1.21
N222812	2020	JingZhou	1	3.29	2.22
N222812	2020	JingZhou	2	2.09	1.63
N222812	2020	JingZhou	3	2.39	1.98
N222813	2020	JingZhou	1	4.73	2.15
N222813	2020	JingZhou	2	4.93	2.22
N222813	2020	JingZhou	3	4.56	2.28
N222814	2020	JingZhou	1	5.35	2.8
N222814	2020	JingZhou	2	4.38	2.59
N222814	2020	JingZhou	3	2.69	2.29

Line	Year	Location	Replication	SD (mm)	BD (mm)
N222815	2020	JingZhou	1	5.81	2.59
N222815	2020	JingZhou	2	3.13	2.55
N222815	2020	JingZhou	3	3.91	2.24
N222816	2020	JingZhou	1	5.42	2.54
N222816	2020	JingZhou	2	5.92	2.92
N222816	2020	JingZhou	3	4.85	2.99
N222817	2020	JingZhou	1	6.59	2.4
N222817	2020	JingZhou	2	5.52	2.71
N222817	2020	JingZhou	3	6.35	2.51
N222818	2020	JingZhou	1	3.4	1.74
N222818	2020	JingZhou	2	-	-
N222818	2020	JingZhou	3	6.74	2.62
N222819	2020	JingZhou	1	6.04	2.67
N222819	2020	JingZhou	2	6.36	2.56
N222819	2020	JingZhou	3	5.5	2.51
N222820	2020	JingZhou	1	2.22	1.78
N222820	2020	JingZhou	2	2.92	2.01
N222820	2020	JingZhou	3	3.07	2.15
N222821	2020	JingZhou	1	4.03	2.28
N222821	2020	JingZhou	2	3.69	1.89
N222821	2020	JingZhou	3	4.42	2.26
N222822	2020	JingZhou	1	4.02	1.85
N222822	2020	JingZhou	2	4.96	1.92
N222822	2020	JingZhou	3	5.31	1.82
N222823	2020	JingZhou	1	3.05	2.44
N222823	2020	JingZhou	2	3.12	2.45
N222823	2020	JingZhou	3	3.02	2.27
N222824	2020	JingZhou	1	2.44	1.56
N222824	2020	JingZhou	2	2.89	1.49
N222824	2020	JingZhou	3	2.29	1.32
N222825	2020	JingZhou	1	2.79	1.75
N222825	2020	JingZhou	2	3.81	1.54
N222825	2020	JingZhou	3	4.32	1.66
N222826	2020	JingZhou	1	4.2	2.52
N222826	2020	JingZhou	2	3.55	2.21
N222826	2020	JingZhou	3	2.9	2.18
N222827	2020	JingZhou	1	2.74	1.81
N222827	2020	JingZhou	2	3.51	2.36
N222827	2020	JingZhou	3	3.18	1.92
N222828	2020	JingZhou	1	3.36	1.92
N222828	2020	JingZhou	2	-	-
N222828	2020	JingZhou	3	-	-
N222829	2020	JingZhou	1	3.23	1.81
N222829	2020	JingZhou	2	3.33	2.27
N222829	2020	JingZhou	3	3.47	1.91

Line	Year	Location	Replication	SD (mm)	BD (mm)
N222830	2020	JingZhou	1	5.43	2.36
N222830	2020	JingZhou	2	5.23	2.2
N222830	2020	JingZhou	3	4.06	2.72
N222831	2020	JingZhou	1	5.6	2.23
N222831	2020	JingZhou	2	4.37	2.22
N222831	2020	JingZhou	3	6.09	2.25
N222832	2020	JingZhou	1	6.99	2.88
N222832	2020	JingZhou	2	9.22	3.06
N222832	2020	JingZhou	3	7.31	3.66
N222833	2020	JingZhou	1	5.38	2.38
N222833	2020	JingZhou	2	4.85	2.68
N222833	2020	JingZhou	3	-	-
N222834	2020	JingZhou	1	3.82	1.96
N222834	2020	JingZhou	2	3.04	2.07
N222834	2020	JingZhou	3	3.21	1.87
N222835	2020	JingZhou	1	7.93	3.76
N222835	2020	JingZhou	2	7.7	2.94
N222835	2020	JingZhou	3	4.68	2.77
N222836	2020	JingZhou	1	1.71	1.27
N222836	2020	JingZhou	2	2.05	1.8
N222836	2020	JingZhou	3	2.98	1.61
N222837	2020	JingZhou	1	4.18	2.37
N222837	2020	JingZhou	2	5.65	2.53
N222837	2020	JingZhou	3	5.8	2.9
N222838	2020	JingZhou	1	5.07	1.91
N222838	2020	JingZhou	2	4.05	2.1
N222838	2020	JingZhou	3	3.18	1.74
N222839	2020	JingZhou	1	3.49	1.8
N222839	2020	JingZhou	2	2.98	1.81
N222839	2020	JingZhou	3	2.9	1.6
N222840	2020	JingZhou	1	3.56	2.06
N222840	2020	JingZhou	2	4.12	2.33
N222840	2020	JingZhou	3	3.47	2.69
N222841	2020	JingZhou	1	2.34	1.86
N222841	2020	JingZhou	2	2.49	2.2
N222841	2020	JingZhou	3	2.66	1.58
N222842	2020	JingZhou	1	6.2	3.15
N222842	2020	JingZhou	2	6.34	3
N222842	2020	JingZhou	3	5.82	2.86
N222843	2020	JingZhou	1	3.46	2.32
N222843	2020	JingZhou	2	3.7	2.26
N222843	2020	JingZhou	3	3.6	2.41
N222844	2020	JingZhou	1	4.04	2.24
N222844	2020	JingZhou	2	3.57	1.99
N222844	2020	JingZhou	3	3.32	2.48

Line	Year	Location	Replication	SD (mm)	BD (mm)
N222845	2020	JingZhou	1	3.62	1.88
N222845	2020	JingZhou	2	2.58	1.75
N222845	2020	JingZhou	3	2.67	2.12
N222846	2020	JingZhou	1	4.84	2.81
N222846	2020	JingZhou	2	5.36	2.73
N222846	2020	JingZhou	3	5.4	2.76
N222847	2020	JingZhou	1	2.45	1.81
N222847	2020	JingZhou	2	2.67	1.78
N222847	2020	JingZhou	3	3.15	1.98
N222848	2020	JingZhou	1	6.47	2.94
N222848	2020	JingZhou	2	6.04	2.54
N222848	2020	JingZhou	3	6.5	2.43
N222849	2020	JingZhou	1	3.89	2.24
N222849	2020	JingZhou	2	3.76	2.1
N222849	2020	JingZhou	3	4.94	2.03
N222850	2020	JingZhou	1	6.65	3.47
N222850	2020	JingZhou	2	11.88	3.73
N222850	2020	JingZhou	3	6.7	3.27
N222851	2020	JingZhou	1	6.01	1.92
N222851	2020	JingZhou	2	-	-
N222851	2020	JingZhou	3	-	-
N222852	2020	JingZhou	1	3.33	2.15
N222852	2020	JingZhou	2	2.08	1.73
N222852	2020	JingZhou	3	2.16	1.84
N222853	2020	JingZhou	1	3.23	1.64
N222853	2020	JingZhou	2	2.79	1.78
N222853	2020	JingZhou	3	3.08	1.91
N222854	2020	JingZhou	1	4.86	2.07
N222854	2020	JingZhou	2	4.18	2.16
N222854	2020	JingZhou	3	4.7	2.24
ZD41	2020	JingZhou	1	4.62	2.6
ZD41	2020	JingZhou	2	3.78	2.64
ZD41	2020	JingZhou	3	3.85	2.52
ZYD02878	2020	JingZhou	1	1.11	0.99
ZYD02878	2020	JingZhou	2	1.04	0.88
ZYD02878	2020	JingZhou	3	-	-
N222488	2021	ShangQiu	1	4.43	-
N222488	2021	ShangQiu	2	-	-
N222488	2021	ShangQiu	3	-	-
N222489	2021	ShangQiu	1	6.03	-
N222489	2021	ShangQiu	2	5.55	-
N222489	2021	ShangQiu	3	5.79	-
N222490	2021	ShangQiu	1	10.14	-
N222490	2021	ShangQiu	2	4.77	-
N222490	2021	ShangQiu	3	4.61	-

Line	Year	Location	Replication	SD (mm)	BD (mm)
N222491	2021	ShangQiu	1	4.32	-
N222491	2021	ShangQiu	2	8.41	-
N222491	2021	ShangQiu	3	8.74	-
N222492	2021	ShangQiu	1	6.99	-
N222492	2021	ShangQiu	2	5.28	-
N222492	2021	ShangQiu	3	4.78	-
N222493	2021	ShangQiu	1	4.42	-
N222493	2021	ShangQiu	2	3.76	-
N222493	2021	ShangQiu	3	4.17	-
N222494	2021	ShangQiu	1	8.36	-
N222494	2021	ShangQiu	2	5.91	-
N222494	2021	ShangQiu	3	7.05	-
N222495	2021	ShangQiu	1	6.07	-
N222495	2021	ShangQiu	2	6.39	-
N222495	2021	ShangQiu	3	5.34	-
N222496	2021	ShangQiu	1	5.32	-
N222496	2021	ShangQiu	2	5.8	-
N222496	2021	ShangQiu	3	5.03	-
N222497	2021	ShangQiu	1	4.92	-
N222497	2021	ShangQiu	2	5.43	-
N222497	2021	ShangQiu	3	7.27	-
N222498	2021	ShangQiu	1	5.2	-
N222498	2021	ShangQiu	2	6.48	-
N222498	2021	ShangQiu	3	5.3	-
N222499	2021	ShangQiu	1	4.69	-
N222499	2021	ShangQiu	2	4.6	-
N222499	2021	ShangQiu	3	5.17	-
N222500	2021	ShangQiu	1	4.22	-
N222500	2021	ShangQiu	2	3.56	-
N222500	2021	ShangQiu	3	2.51	-
N222501	2021	ShangQiu	1	4.72	-
N222501	2021	ShangQiu	2	5.22	-
N222501	2021	ShangQiu	3	5.09	-
N222502	2021	ShangQiu	1	6.69	-
N222502	2021	ShangQiu	2	5.02	-
N222502	2021	ShangQiu	3	4.25	-
N222503	2021	ShangQiu	1	5	-
N222503	2021	ShangQiu	2	5.56	-
N222503	2021	ShangQiu	3	4.31	-
N222504	2021	ShangQiu	1	4.98	-
N222504	2021	ShangQiu	2	5.55	-
N222504	2021	ShangQiu	3	5.06	-
N222505	2021	ShangQiu	1	5.48	-
N222505	2021	ShangQiu	2	6.9	-
N222505	2021	ShangQiu	3	6.15	-

Line	Year	Location	Replication	SD (mm)	BD (mm)
N222506	2021	ShangQiu	1	5.94	-
N222506	2021	ShangQiu	2	5.6	-
N222506	2021	ShangQiu	3	3.15	-
N222507	2021	ShangQiu	1	6.29	-
N222507	2021	ShangQiu	2	4	-
N222507	2021	ShangQiu	3	6.8	-
N222508	2021	ShangQiu	1	5.55	-
N222508	2021	ShangQiu	2	6.48	-
N222508	2021	ShangQiu	3	4.93	-
N222509	2021	ShangQiu	1	4.11	-
N222509	2021	ShangQiu	2	5.35	-
N222509	2021	ShangQiu	3	3.03	-
N222510	2021	ShangQiu	1	4.84	-
N222510	2021	ShangQiu	2	5.7	-
N222510	2021	ShangQiu	3	4.94	-
N222511	2021	ShangQiu	1	5.23	-
N222511	2021	ShangQiu	2	5.48	-
N222511	2021	ShangQiu	3	4.2	-
N222512	2021	ShangQiu	1	7.1	-
N222512	2021	ShangQiu	2	8.42	-
N222512	2021	ShangQiu	3	8.14	-
N222513	2021	ShangQiu	1	4.77	-
N222513	2021	ShangQiu	2	3.33	-
N222513	2021	ShangQiu	3	4.26	-
N222514	2021	ShangQiu	1	4.19	-
N222514	2021	ShangQiu	2	11.47	-
N222514	2021	ShangQiu	3	5.64	-
N222515	2021	ShangQiu	1	7.36	-
N222515	2021	ShangQiu	2	5.15	-
N222515	2021	ShangQiu	3	6.23	-
N222516	2021	ShangQiu	1	3.85	-
N222516	2021	ShangQiu	2	4.92	-
N222516	2021	ShangQiu	3	5.72	-
N222517	2021	ShangQiu	1	4.29	-
N222517	2021	ShangQiu	2	4.13	-
N222517	2021	ShangQiu	3	6.3	-
N222518	2021	ShangQiu	1	3.46	-
N222518	2021	ShangQiu	2	4.41	-
N222518	2021	ShangQiu	3	5.38	-
N222519	2021	ShangQiu	1	4.76	-
N222519	2021	ShangQiu	2	2.74	-
N222519	2021	ShangQiu	3	5.29	-
N222520	2021	ShangQiu	1	5.49	-
N222520	2021	ShangQiu	2	5.37	-
N222520	2021	ShangQiu	3	4.7	-

Line	Year	Location	Replication	SD (mm)	BD (mm)
N222521	2021	ShangQiu	1	7.06	-
N222521	2021	ShangQiu	2	6.54	-
N222521	2021	ShangQiu	3	8.04	-
N222522	2021	ShangQiu	1	5.09	-
N222522	2021	ShangQiu	2	4.92	-
N222522	2021	ShangQiu	3	3.87	-
N222523	2021	ShangQiu	1	8.65	-
N222523	2021	ShangQiu	2	7.7	-
N222523	2021	ShangQiu	3	6.73	-
N222524	2021	ShangQiu	1	4.78	-
N222524	2021	ShangQiu	2	7.11	-
N222524	2021	ShangQiu	3	5.7	-
N222525	2021	ShangQiu	1	5.42	-
N222525	2021	ShangQiu	2	-	-
N222525	2021	ShangQiu	3	-	-
N222526	2021	ShangQiu	1	4.86	-
N222526	2021	ShangQiu	2	5.38	-
N222526	2021	ShangQiu	3	3.98	-
N222527	2021	ShangQiu	1	5.58	-
N222527	2021	ShangQiu	2	5.61	-
N222527	2021	ShangQiu	3	4.59	-
N222528	2021	ShangQiu	1	4.45	-
N222528	2021	ShangQiu	2	4.15	-
N222528	2021	ShangQiu	3	5.58	-
N222529	2021	ShangQiu	1	5.48	-
N222529	2021	ShangQiu	2	5.29	-
N222529	2021	ShangQiu	3	2.63	-
N222530	2021	ShangQiu	1	3.79	-
N222530	2021	ShangQiu	2	4.14	-
N222530	2021	ShangQiu	3	5.2	-
N222531	2021	ShangQiu	1	4.68	-
N222531	2021	ShangQiu	2	3.57	-
N222531	2021	ShangQiu	3	3.81	-
N222532	2021	ShangQiu	1	-	-
N222532	2021	ShangQiu	2	-	-
N222532	2021	ShangQiu	3	-	-
N222533	2021	ShangQiu	1	6.45	-
N222533	2021	ShangQiu	2	4.8	-
N222533	2021	ShangQiu	3	5.82	-
N222534	2021	ShangQiu	1	-	-
N222534	2021	ShangQiu	2	-	-
N222534	2021	ShangQiu	3	-	-
N222535	2021	ShangQiu	1	6.88	-
N222535	2021	ShangQiu	2	6.37	-
N222535	2021	ShangQiu	3	4	-

Line	Year	Location	Replication	SD (mm)	BD (mm)
N222536	2021	ShangQiu	1	5.29	-
N222536	2021	ShangQiu	2	4.08	-
N222536	2021	ShangQiu	3	3.16	-
N222537	2021	ShangQiu	1	-	-
N222537	2021	ShangQiu	2	-	-
N222537	2021	ShangQiu	3	-	-
N222538	2021	ShangQiu	1	5.92	-
N222538	2021	ShangQiu	2	4.75	-
N222538	2021	ShangQiu	3	5.86	-
N222539	2021	ShangQiu	1	4.59	-
N222539	2021	ShangQiu	2	2.64	-
N222539	2021	ShangQiu	3	7.3	-
N222540	2021	ShangQiu	1	4.98	-
N222540	2021	ShangQiu	2	5.72	-
N222540	2021	ShangQiu	3	-	-
N222541	2021	ShangQiu	1	6.69	-
N222541	2021	ShangQiu	2	7.59	-
N222541	2021	ShangQiu	3	2.44	-
N222542	2021	ShangQiu	1	4.88	-
N222542	2021	ShangQiu	2	5.11	-
N222542	2021	ShangQiu	3	-	-
N222543	2021	ShangQiu	1	6.37	-
N222543	2021	ShangQiu	2	6.59	-
N222543	2021	ShangQiu	3	7.8	-
N222544	2021	ShangQiu	1	4.65	-
N222544	2021	ShangQiu	2	4.03	-
N222544	2021	ShangQiu	3	5.01	-
N222545	2021	ShangQiu	1	5.99	-
N222545	2021	ShangQiu	2	6.04	-
N222545	2021	ShangQiu	3	5.18	-
N222546	2021	ShangQiu	1	5.72	-
N222546	2021	ShangQiu	2	6.41	-
N222546	2021	ShangQiu	3	4.7	-
N222547	2021	ShangQiu	1	5.75	-
N222547	2021	ShangQiu	2	6.51	-
N222547	2021	ShangQiu	3	6.07	-
N222548	2021	ShangQiu	1	6.59	-
N222548	2021	ShangQiu	2	6.51	-
N222548	2021	ShangQiu	3	7.62	-
N222549	2021	ShangQiu	1	5.69	-
N222549	2021	ShangQiu	2	5.5	-
N222549	2021	ShangQiu	3	5.54	-
N222550	2021	ShangQiu	1	7.63	-
N222550	2021	ShangQiu	2	7.17	-
N222550	2021	ShangQiu	3	7.57	-

Line	Year	Location	Replication	SD (mm)	BD (mm)
N222551	2021	ShangQiu	1	5.38	-
N222551	2021	ShangQiu	2	3.39	-
N222551	2021	ShangQiu	3	4.49	-
N222552	2021	ShangQiu	1	4.56	-
N222552	2021	ShangQiu	2	4.87	-
N222552	2021	ShangQiu	3	6.3	-
N222553	2021	ShangQiu	1	6.11	-
N222553	2021	ShangQiu	2	-	-
N222553	2021	ShangQiu	3	-	-
N222554	2021	ShangQiu	1	4.4	-
N222554	2021	ShangQiu	2	4.26	-
N222554	2021	ShangQiu	3	3.97	-
N222555	2021	ShangQiu	1	4.26	-
N222555	2021	ShangQiu	2	4.61	-
N222555	2021	ShangQiu	3	6.15	-
N222556	2021	ShangQiu	1	6.03	-
N222556	2021	ShangQiu	2	6.51	-
N222556	2021	ShangQiu	3	5.54	-
N222557	2021	ShangQiu	1	6.75	-
N222557	2021	ShangQiu	2	5.87	-
N222557	2021	ShangQiu	3	6.36	-
N222558	2021	ShangQiu	1	3.24	-
N222558	2021	ShangQiu	2	5.83	-
N222558	2021	ShangQiu	3	-	-
N222559	2021	ShangQiu	1	5.44	-
N222559	2021	ShangQiu	2	4.15	-
N222559	2021	ShangQiu	3	7.9	-
N222560	2021	ShangQiu	1	6.14	-
N222560	2021	ShangQiu	2	6.09	-
N222560	2021	ShangQiu	3	7.39	-
N222561	2021	ShangQiu	1	6.02	-
N222561	2021	ShangQiu	2	4.41	-
N222561	2021	ShangQiu	3	5.11	-
N222562	2021	ShangQiu	1	7.01	-
N222562	2021	ShangQiu	2	7.36	-
N222562	2021	ShangQiu	3	7.13	-
N222563	2021	ShangQiu	1	4.53	-
N222563	2021	ShangQiu	2	4.43	-
N222563	2021	ShangQiu	3	4.72	-
N222564	2021	ShangQiu	1	-	-
N222564	2021	ShangQiu	2	-	-
N222564	2021	ShangQiu	3	-	-
N222565	2021	ShangQiu	1	8.72	-
N222565	2021	ShangQiu	2	4.84	-
N222565	2021	ShangQiu	3	8.18	-

Line	Year	Location	Replication	SD (mm)	BD (mm)
N222566	2021	ShangQiu	1	6.9	-
N222566	2021	ShangQiu	2	6.7	-
N222566	2021	ShangQiu	3	7.21	-
N222567	2021	ShangQiu	1	6.12	-
N222567	2021	ShangQiu	2	6.15	-
N222567	2021	ShangQiu	3	7.52	-
N222568	2021	ShangQiu	1	7.22	-
N222568	2021	ShangQiu	2	-	-
N222568	2021	ShangQiu	3	6.25	-
N222569	2021	ShangQiu	1	4.89	-
N222569	2021	ShangQiu	2	4.62	-
N222569	2021	ShangQiu	3	3.58	-
N222570	2021	ShangQiu	1	6.66	-
N222570	2021	ShangQiu	2	6.42	-
N222570	2021	ShangQiu	3	5.97	-
N222571	2021	ShangQiu	1	5.37	-
N222571	2021	ShangQiu	2	4.03	-
N222571	2021	ShangQiu	3	6.11	-
N222572	2021	ShangQiu	1	4.22	-
N222572	2021	ShangQiu	2	5.95	-
N222572	2021	ShangQiu	3	6.17	-
N222573	2021	ShangQiu	1	3.62	-
N222573	2021	ShangQiu	2	7.57	-
N222573	2021	ShangQiu	3	8.18	-
N222574	2021	ShangQiu	1	4.99	-
N222574	2021	ShangQiu	2	4.74	-
N222574	2021	ShangQiu	3	5.82	-
N222575	2021	ShangQiu	1	5.89	-
N222575	2021	ShangQiu	2	4.48	-
N222575	2021	ShangQiu	3	3.56	-
N222576	2021	ShangQiu	1	6.12	-
N222576	2021	ShangQiu	2	6.74	-
N222576	2021	ShangQiu	3	7.36	-
N222577	2021	ShangQiu	1	6.03	-
N222577	2021	ShangQiu	2	6.88	-
N222577	2021	ShangQiu	3	5.26	-
N222578	2021	ShangQiu	1	4.07	-
N222578	2021	ShangQiu	2	4.16	-
N222578	2021	ShangQiu	3	4.67	-
N222579	2021	ShangQiu	1	9.17	-
N222579	2021	ShangQiu	2	8.96	-
N222579	2021	ShangQiu	3	9.15	-
N222580	2021	ShangQiu	1	9.98	-
N222580	2021	ShangQiu	2	-	-
N222580	2021	ShangQiu	3	-	-

Line	Year	Location	Replication	SD (mm)	BD (mm)
N222581	2021	ShangQiu	1	6.97	-
N222581	2021	ShangQiu	2	6.21	-
N222581	2021	ShangQiu	3	7.01	-
N222582	2021	ShangQiu	1	8.66	-
N222582	2021	ShangQiu	2	7.63	-
N222582	2021	ShangQiu	3	-	-
N222583	2021	ShangQiu	1	4.57	-
N222583	2021	ShangQiu	2	7.12	-
N222583	2021	ShangQiu	3	6.31	-
N222584	2021	ShangQiu	1	6.48	-
N222584	2021	ShangQiu	2	3.26	-
N222584	2021	ShangQiu	3	4.92	-
N222585	2021	ShangQiu	1	5.62	-
N222585	2021	ShangQiu	2	6.97	-
N222585	2021	ShangQiu	3	7.47	-
N222586	2021	ShangQiu	1	6.07	-
N222586	2021	ShangQiu	2	5.71	-
N222586	2021	ShangQiu	3	6.15	-
N222587	2021	ShangQiu	1	6.16	-
N222587	2021	ShangQiu	2	6.16	-
N222587	2021	ShangQiu	3	6.72	-
N222588	2021	ShangQiu	1	5.69	-
N222588	2021	ShangQiu	2	4.04	-
N222588	2021	ShangQiu	3	4.85	-
N222589	2021	ShangQiu	1	5.87	-
N222589	2021	ShangQiu	2	4.14	-
N222589	2021	ShangQiu	3	5.38	-
N222590	2021	ShangQiu	1	3.25	-
N222590	2021	ShangQiu	2	4.95	-
N222590	2021	ShangQiu	3	5.33	-
N222591	2021	ShangQiu	1	4.74	-
N222591	2021	ShangQiu	2	4.63	-
N222591	2021	ShangQiu	3	4.26	-
N222592	2021	ShangQiu	1	6.66	-
N222592	2021	ShangQiu	2	6.81	-
N222592	2021	ShangQiu	3	6.62	-
N222593	2021	ShangQiu	1	2.9	-
N222593	2021	ShangQiu	2	3.57	-
N222593	2021	ShangQiu	3	4.24	-
N222594	2021	ShangQiu	1	4.09	-
N222594	2021	ShangQiu	2	4.79	-
N222594	2021	ShangQiu	3	-	-
N222595	2021	ShangQiu	1	4.76	-
N222595	2021	ShangQiu	2	4.2	-
N222595	2021	ShangQiu	3	5.52	-

Line	Year	Location	Replication	SD (mm)	BD (mm)
N222596	2021	ShangQiu	1	4.71	-
N222596	2021	ShangQiu	2	3.36	-
N222596	2021	ShangQiu	3	-	-
N222597	2021	ShangQiu	1	9.29	-
N222597	2021	ShangQiu	2	8.13	-
N222597	2021	ShangQiu	3	9.26	-
N222598	2021	ShangQiu	1	5.02	-
N222598	2021	ShangQiu	2	2.6	-
N222598	2021	ShangQiu	3	3.38	-
N222599	2021	ShangQiu	1	5.35	-
N222599	2021	ShangQiu	2	7.67	-
N222599	2021	ShangQiu	3	5.89	-
N222600	2021	ShangQiu	1	5.52	-
N222600	2021	ShangQiu	2	5.67	-
N222600	2021	ShangQiu	3	4.84	-
N222601	2021	ShangQiu	1	6.07	-
N222601	2021	ShangQiu	2	5.14	-
N222601	2021	ShangQiu	3	6.85	-
N222602	2021	ShangQiu	1	4.48	-
N222602	2021	ShangQiu	2	3.76	-
N222602	2021	ShangQiu	3	3.26	-
N222603	2021	ShangQiu	1	4.65	-
N222603	2021	ShangQiu	2	7.65	-
N222603	2021	ShangQiu	3	5.96	-
N222604	2021	ShangQiu	1	4.15	-
N222604	2021	ShangQiu	2	4.33	-
N222604	2021	ShangQiu	3	4.26	-
N222605	2021	ShangQiu	1	6.61	-
N222605	2021	ShangQiu	2	6.94	-
N222605	2021	ShangQiu	3	4.63	-
N222606	2021	ShangQiu	1	5.42	-
N222606	2021	ShangQiu	2	5.1	-
N222606	2021	ShangQiu	3	5.77	-
N222607	2021	ShangQiu	1	5.8	-
N222607	2021	ShangQiu	2	7.24	-
N222607	2021	ShangQiu	3	4.89	-
N222608	2021	ShangQiu	1	5.38	-
N222608	2021	ShangQiu	2	5.73	-
N222608	2021	ShangQiu	3	7.16	-
N222609	2021	ShangQiu	1	2.22	-
N222609	2021	ShangQiu	2	3.08	-
N222609	2021	ShangQiu	3	-	-
N222610	2021	ShangQiu	1	5.36	-
N222610	2021	ShangQiu	2	7.22	-
N222610	2021	ShangQiu	3	6.67	-

Line	Year	Location	Replication	SD (mm)	BD (mm)
N222611	2021	ShangQiu	1	4.1	-
N222611	2021	ShangQiu	2	4.4	-
N222611	2021	ShangQiu	3	4.24	-
N222612	2021	ShangQiu	1	4.55	-
N222612	2021	ShangQiu	2	5.24	-
N222612	2021	ShangQiu	3	6.17	-
N222613	2021	ShangQiu	1	4.5	-
N222613	2021	ShangQiu	2	3.15	-
N222613	2021	ShangQiu	3	-	-
N222614	2021	ShangQiu	1	6.12	-
N222614	2021	ShangQiu	2	3.77	-
N222614	2021	ShangQiu	3	5.11	-
N222615	2021	ShangQiu	1	5.36	-
N222615	2021	ShangQiu	2	4.05	-
N222615	2021	ShangQiu	3	4.44	-
N222616	2021	ShangQiu	1	6.12	-
N222616	2021	ShangQiu	2	6.66	-
N222616	2021	ShangQiu	3	5.39	-
N222617	2021	ShangQiu	1	3.59	-
N222617	2021	ShangQiu	2	4.25	-
N222617	2021	ShangQiu	3	4.37	-
N222618	2021	ShangQiu	1	4.61	-
N222618	2021	ShangQiu	2	4.75	-
N222618	2021	ShangQiu	3	5.4	-
N222619	2021	ShangQiu	1	4.75	-
N222619	2021	ShangQiu	2	3.82	-
N222619	2021	ShangQiu	3	4.44	-
N222620	2021	ShangQiu	1	4.69	-
N222620	2021	ShangQiu	2	-	-
N222620	2021	ShangQiu	3	4.19	-
N222621	2021	ShangQiu	1	6.04	-
N222621	2021	ShangQiu	2	4.2	-
N222621	2021	ShangQiu	3	7.13	-
N222622	2021	ShangQiu	1	5.16	-
N222622	2021	ShangQiu	2	3.47	-
N222622	2021	ShangQiu	3	-	-
N222623	2021	ShangQiu	1	3.86	-
N222623	2021	ShangQiu	2	4.14	-
N222623	2021	ShangQiu	3	5.27	-
N222624	2021	ShangQiu	1	4.65	-
N222624	2021	ShangQiu	2	3.65	-
N222624	2021	ShangQiu	3	4.42	-
N222625	2021	ShangQiu	1	4.63	-
N222625	2021	ShangQiu	2	4.03	-
N222625	2021	ShangQiu	3	5.7	-

Line	Year	Location	Replication	SD (mm)	BD (mm)
N222628	2021	ShangQiu	1	5.53	-
N222628	2021	ShangQiu	2	4.71	-
N222628	2021	ShangQiu	3	5.34	-
N222629	2021	ShangQiu	1	3.84	-
N222629	2021	ShangQiu	2	4.55	-
N222629	2021	ShangQiu	3	5.08	-
N222630	2021	ShangQiu	1	6.74	-
N222630	2021	ShangQiu	2	7.14	-
N222630	2021	ShangQiu	3	6.73	-
N222631	2021	ShangQiu	1	4.12	-
N222631	2021	ShangQiu	2	5.21	-
N222631	2021	ShangQiu	3	4.13	-
N222632	2021	ShangQiu	1	5.58	-
N222632	2021	ShangQiu	2	6.31	-
N222632	2021	ShangQiu	3	4.89	-
N222633	2021	ShangQiu	1	5.53	-
N222633	2021	ShangQiu	2	7.43	-
N222633	2021	ShangQiu	3	5.24	-
N222634	2021	ShangQiu	1	3.78	-
N222634	2021	ShangQiu	2	4.38	-
N222634	2021	ShangQiu	3	4.24	-
N222635	2021	ShangQiu	1	2.66	-
N222635	2021	ShangQiu	2	3.34	-
N222635	2021	ShangQiu	3	2.94	-
N222636	2021	ShangQiu	1	3.8	-
N222636	2021	ShangQiu	2	6.04	-
N222636	2021	ShangQiu	3	6.13	-
N222637	2021	ShangQiu	1	4.29	-
N222637	2021	ShangQiu	2	5.81	-
N222637	2021	ShangQiu	3	5.25	-
N222638	2021	ShangQiu	1	4.41	-
N222638	2021	ShangQiu	2	-	-
N222638	2021	ShangQiu	3	-	-
N222639	2021	ShangQiu	1	4.26	-
N222639	2021	ShangQiu	2	7.16	-
N222639	2021	ShangQiu	3	7.05	-
N222640	2021	ShangQiu	1	5.05	-
N222640	2021	ShangQiu	2	5.29	-
N222640	2021	ShangQiu	3	5.03	-
N222641	2021	ShangQiu	1	7.38	-
N222641	2021	ShangQiu	2	6.19	-
N222641	2021	ShangQiu	3	11.7	-
N222642	2021	ShangQiu	1	5.98	-
N222642	2021	ShangQiu	2	6.43	-
N222642	2021	ShangQiu	3	5.9	-

Line	Year	Location	Replication	SD (mm)	BD (mm)
N222643	2021	ShangQiu	1	6.28	-
N222643	2021	ShangQiu	2	-	-
N222643	2021	ShangQiu	3	-	-
N222644	2021	ShangQiu	1	4.72	-
N222644	2021	ShangQiu	2	5.65	-
N222644	2021	ShangQiu	3	3.62	-
N222645	2021	ShangQiu	1	4.52	-
N222645	2021	ShangQiu	2	4.95	-
N222645	2021	ShangQiu	3	6.16	-
N222646	2021	ShangQiu	1	6.87	-
N222646	2021	ShangQiu	2	5.51	-
N222646	2021	ShangQiu	3	5.9	-
N222647	2021	ShangQiu	1	7.3	-
N222647	2021	ShangQiu	2	6.73	-
N222647	2021	ShangQiu	3	8.47	-
N222648	2021	ShangQiu	1	4.47	-
N222648	2021	ShangQiu	2	2.83	-
N222648	2021	ShangQiu	3	2.73	-
N222649	2021	ShangQiu	1	5.08	-
N222649	2021	ShangQiu	2	4.12	-
N222649	2021	ShangQiu	3	-	-
N222650	2021	ShangQiu	1	7.06	-
N222650	2021	ShangQiu	2	7.32	-
N222650	2021	ShangQiu	3	6.3	-
N222651	2021	ShangQiu	1	6.2	-
N222651	2021	ShangQiu	2	5.21	-
N222651	2021	ShangQiu	3	7.19	-
N222652	2021	ShangQiu	1	6.69	-
N222652	2021	ShangQiu	2	6.65	-
N222652	2021	ShangQiu	3	7.05	-
N222653	2021	ShangQiu	1	5.07	-
N222653	2021	ShangQiu	2	3.78	-
N222653	2021	ShangQiu	3	4.58	-
N222654	2021	ShangQiu	1	6.79	-
N222654	2021	ShangQiu	2	7.31	-
N222654	2021	ShangQiu	3	6.15	-
N222655	2021	ShangQiu	1	7.28	-
N222655	2021	ShangQiu	2	7.8	-
N222655	2021	ShangQiu	3	8.18	-
N222656	2021	ShangQiu	1	5.61	-
N222656	2021	ShangQiu	2	4.83	-
N222656	2021	ShangQiu	3	6.22	-
N222657	2021	ShangQiu	1	5.37	-
N222657	2021	ShangQiu	2	5.1	-
N222657	2021	ShangQiu	3	4.91	-

Line	Year	Location	Replication	SD (mm)	BD (mm)
N222658	2021	ShangQiu	1	9.44	-
N222658	2021	ShangQiu	2	9.12	-
N222658	2021	ShangQiu	3	9.71	-
N222659	2021	ShangQiu	1	9.42	-
N222659	2021	ShangQiu	2	8.52	-
N222659	2021	ShangQiu	3	5.38	-
N222660	2021	ShangQiu	1	3.88	-
N222660	2021	ShangQiu	2	3.63	-
N222660	2021	ShangQiu	3	5.83	-
N222661	2021	ShangQiu	1	6.98	-
N222661	2021	ShangQiu	2	5.48	-
N222661	2021	ShangQiu	3	6.04	-
N222662	2021	ShangQiu	1	-	-
N222662	2021	ShangQiu	2	-	-
N222662	2021	ShangQiu	3	-	-
N222663	2021	ShangQiu	1	5.31	-
N222663	2021	ShangQiu	2	7.39	-
N222663	2021	ShangQiu	3	4.47	-
N222664	2021	ShangQiu	1	2.2	-
N222664	2021	ShangQiu	2	4.09	-
N222664	2021	ShangQiu	3	2.83	-
N222665	2021	ShangQiu	1	4.02	-
N222665	2021	ShangQiu	2	5.28	-
N222665	2021	ShangQiu	3	4.64	-
N222666	2021	ShangQiu	1	4.97	-
N222666	2021	ShangQiu	2	5.45	-
N222666	2021	ShangQiu	3	4.05	-
N222667	2021	ShangQiu	1	7.06	-
N222667	2021	ShangQiu	2	6.05	-
N222667	2021	ShangQiu	3	5.98	-
N222668	2021	ShangQiu	1	4.17	-
N222668	2021	ShangQiu	2	-	-
N222668	2021	ShangQiu	3	4.23	-
N222669	2021	ShangQiu	1	5.94	-
N222669	2021	ShangQiu	2	5.94	-
N222669	2021	ShangQiu	3	5.34	-
N222670	2021	ShangQiu	1	5.43	-
N222670	2021	ShangQiu	2	6.39	-
N222670	2021	ShangQiu	3	-	-
N222671	2021	ShangQiu	1	5.33	-
N222671	2021	ShangQiu	2	6.1	-
N222671	2021	ShangQiu	3	5.47	-
N222672	2021	ShangQiu	1	4.81	-
N222672	2021	ShangQiu	2	4.03	-
N222672	2021	ShangQiu	3	5.07	-

Line	Year	Location	Replication	SD (mm)	BD (mm)
N222673	2021	ShangQiu	1	-	-
N222673	2021	ShangQiu	2	-	-
N222673	2021	ShangQiu	3	-	-
N222674	2021	ShangQiu	1	6.05	-
N222674	2021	ShangQiu	2	5.33	-
N222674	2021	ShangQiu	3	5.47	-
N222675	2021	ShangQiu	1	3.32	-
N222675	2021	ShangQiu	2	2.73	-
N222675	2021	ShangQiu	3	2.48	-
N222676	2021	ShangQiu	1	6.19	-
N222676	2021	ShangQiu	2	-	-
N222676	2021	ShangQiu	3	7.9	-
N222677	2021	ShangQiu	1	4.7	-
N222677	2021	ShangQiu	2	4.39	-
N222677	2021	ShangQiu	3	5.08	-
N222678	2021	ShangQiu	1	5.53	-
N222678	2021	ShangQiu	2	5.35	-
N222678	2021	ShangQiu	3	3.89	-
N222679	2021	ShangQiu	1	6.18	-
N222679	2021	ShangQiu	2	3.93	-
N222679	2021	ShangQiu	3	4.38	-
N222680	2021	ShangQiu	1	4.76	-
N222680	2021	ShangQiu	2	7.26	-
N222680	2021	ShangQiu	3	4.5	-
N222681	2021	ShangQiu	1	9.67	-
N222681	2021	ShangQiu	2	8.78	-
N222681	2021	ShangQiu	3	7.32	-
N222682	2021	ShangQiu	1	7.13	-
N222682	2021	ShangQiu	2	4.63	-
N222682	2021	ShangQiu	3	6.19	-
N222683	2021	ShangQiu	1	9.22	-
N222683	2021	ShangQiu	2	5.84	-
N222683	2021	ShangQiu	3	7.16	-
N222684	2021	ShangQiu	1	6.42	-
N222684	2021	ShangQiu	2	6.6	-
N222684	2021	ShangQiu	3	5.86	-
N222685	2021	ShangQiu	1	10.88	-
N222685	2021	ShangQiu	2	-	-
N222685	2021	ShangQiu	3	9.98	-
N222686	2021	ShangQiu	1	6.22	-
N222686	2021	ShangQiu	2	6.16	-
N222686	2021	ShangQiu	3	6.18	-
N222687	2021	ShangQiu	1	5.05	-
N222687	2021	ShangQiu	2	5.81	-
N222687	2021	ShangQiu	3	5.19	-

Line	Year	Location	Replication	SD (mm)	BD (mm)
N222688	2021	ShangQiu	1	4.6	-
N222688	2021	ShangQiu	2	3.91	-
N222688	2021	ShangQiu	3	5.02	-
N222689	2021	ShangQiu	1	5.81	-
N222689	2021	ShangQiu	2	4.88	-
N222689	2021	ShangQiu	3	5.24	-
N222690	2021	ShangQiu	1	-	-
N222690	2021	ShangQiu	2	-	-
N222690	2021	ShangQiu	3	7	-
N222691	2021	ShangQiu	1	3.47	-
N222691	2021	ShangQiu	2	4.26	-
N222691	2021	ShangQiu	3	-	-
N222692	2021	ShangQiu	1	5.29	-
N222692	2021	ShangQiu	2	4.31	-
N222692	2021	ShangQiu	3	5.38	-
N222693	2021	ShangQiu	1	6.89	-
N222693	2021	ShangQiu	2	4.52	-
N222693	2021	ShangQiu	3	5.05	-
N222694	2021	ShangQiu	1	4.61	-
N222694	2021	ShangQiu	2	4.79	-
N222694	2021	ShangQiu	3	4.89	-
N222695	2021	ShangQiu	1	4.07	-
N222695	2021	ShangQiu	2	5.72	-
N222695	2021	ShangQiu	3	5.16	-
N222696	2021	ShangQiu	1	5.04	-
N222696	2021	ShangQiu	2	5.55	-
N222696	2021	ShangQiu	3	4.43	-
N222697	2021	ShangQiu	1	3.17	-
N222697	2021	ShangQiu	2	4.24	-
N222697	2021	ShangQiu	3	3.13	-
N222698	2021	ShangQiu	1	5.79	-
N222698	2021	ShangQiu	2	4.1	-
N222698	2021	ShangQiu	3	4.35	-
N222699	2021	ShangQiu	1	-	-
N222699	2021	ShangQiu	2	-	-
N222699	2021	ShangQiu	3	-	-
N222700	2021	ShangQiu	1	6.34	-
N222700	2021	ShangQiu	2	4.48	-
N222700	2021	ShangQiu	3	5.06	-
N222701	2021	ShangQiu	1	7.49	-
N222701	2021	ShangQiu	2	5.16	-
N222701	2021	ShangQiu	3	6.86	-
N222702	2021	ShangQiu	1	5.82	-
N222702	2021	ShangQiu	2	6.02	-
N222702	2021	ShangQiu	3	5.72	-

Line	Year	Location	Replication	SD (mm)	BD (mm)
N222703	2021	ShangQiu	1	-	-
N222703	2021	ShangQiu	2	-	-
N222703	2021	ShangQiu	3	-	-
N222704	2021	ShangQiu	1	5.74	-
N222704	2021	ShangQiu	2	6.35	-
N222704	2021	ShangQiu	3	7.62	-
N222705	2021	ShangQiu	1	5.72	-
N222705	2021	ShangQiu	2	2.55	-
N222705	2021	ShangQiu	3	5.72	-
N222706	2021	ShangQiu	1	5.84	-
N222706	2021	ShangQiu	2	4.99	-
N222706	2021	ShangQiu	3	5.89	-
N222707	2021	ShangQiu	1	4.48	-
N222707	2021	ShangQiu	2	4.53	-
N222707	2021	ShangQiu	3	5.48	-
N222708	2021	ShangQiu	1	5.44	-
N222708	2021	ShangQiu	2	3.77	-
N222708	2021	ShangQiu	3	-	-
N222709	2021	ShangQiu	1	5.66	-
N222709	2021	ShangQiu	2	5.52	-
N222709	2021	ShangQiu	3	5.9	-
N222710	2021	ShangQiu	1	-	-
N222710	2021	ShangQiu	2	-	-
N222710	2021	ShangQiu	3	-	-
N222711	2021	ShangQiu	1	4.36	-
N222711	2021	ShangQiu	2	4.38	-
N222711	2021	ShangQiu	3	4.76	-
N222712	2021	ShangQiu	1	5.52	-
N222712	2021	ShangQiu	2	5.35	-
N222712	2021	ShangQiu	3	4.18	-
N222713	2021	ShangQiu	1	5.69	-
N222713	2021	ShangQiu	2	5.37	-
N222713	2021	ShangQiu	3	6.2	-
N222715	2021	ShangQiu	1	6.28	-
N222715	2021	ShangQiu	2	6.48	-
N222715	2021	ShangQiu	3	6.32	-
N222716	2021	ShangQiu	1	5.94	-
N222716	2021	ShangQiu	2	8.75	-
N222716	2021	ShangQiu	3	-	-
N222717	2021	ShangQiu	1	7.44	-
N222717	2021	ShangQiu	2	9.3	-
N222717	2021	ShangQiu	3	9.66	-
N222718	2021	ShangQiu	1	4.77	-
N222718	2021	ShangQiu	2	8.6	-
N222718	2021	ShangQiu	3	6.57	-

Line	Year	Location	Replication	SD (mm)	BD (mm)
N222719	2021	ShangQiu	1	7.57	-
N222719	2021	ShangQiu	2	3.78	-
N222719	2021	ShangQiu	3	6.44	-
N222720	2021	ShangQiu	1	7.2	-
N222720	2021	ShangQiu	2	7.39	-
N222720	2021	ShangQiu	3	6.53	-
N222721	2021	ShangQiu	1	2.9	-
N222721	2021	ShangQiu	2	3.64	-
N222721	2021	ShangQiu	3	5.42	-
N222722	2021	ShangQiu	1	3.81	-
N222722	2021	ShangQiu	2	5.13	-
N222722	2021	ShangQiu	3	8.68	-
N222723	2021	ShangQiu	1	5.08	-
N222723	2021	ShangQiu	2	3.11	-
N222723	2021	ShangQiu	3	5.17	-
N222724	2021	ShangQiu	1	6.4	-
N222724	2021	ShangQiu	2	5.94	-
N222724	2021	ShangQiu	3	5.67	-
N222725	2021	ShangQiu	1	4.97	-
N222725	2021	ShangQiu	2	-	-
N222725	2021	ShangQiu	3	-	-
N222726	2021	ShangQiu	1	4.71	-
N222726	2021	ShangQiu	2	5.74	-
N222726	2021	ShangQiu	3	5.89	-
N222727	2021	ShangQiu	1	-	-
N222727	2021	ShangQiu	2	-	-
N222727	2021	ShangQiu	3	-	-
N222728	2021	ShangQiu	1	5.61	-
N222728	2021	ShangQiu	2	5.73	-
N222728	2021	ShangQiu	3	5.22	-
N222729	2021	ShangQiu	1	6.13	-
N222729	2021	ShangQiu	2	5.19	-
N222729	2021	ShangQiu	3	5.61	-
N222730	2021	ShangQiu	1	6.04	-
N222730	2021	ShangQiu	2	5.47	-
N222730	2021	ShangQiu	3	6.35	-
N222731	2021	ShangQiu	1	5.2	-
N222731	2021	ShangQiu	2	3	-
N222731	2021	ShangQiu	3	5.27	-
N222732	2021	ShangQiu	1	6.42	-
N222732	2021	ShangQiu	2	7.41	-
N222732	2021	ShangQiu	3	3.55	-
N222733	2021	ShangQiu	1	4.83	-
N222733	2021	ShangQiu	2	7.1	-
N222733	2021	ShangQiu	3	6.58	-

Line	Year	Location	Replication	SD (mm)	BD (mm)
N222734	2021	ShangQiu	1	5.72	-
N222734	2021	ShangQiu	2	7.26	-
N222734	2021	ShangQiu	3	7.32	-
N222735	2021	ShangQiu	1	6.31	-
N222735	2021	ShangQiu	2	9.13	-
N222735	2021	ShangQiu	3	5.71	-
N222736	2021	ShangQiu	1	5.02	-
N222736	2021	ShangQiu	2	6.49	-
N222736	2021	ShangQiu	3	4.9	-
N222737	2021	ShangQiu	1	3.96	-
N222737	2021	ShangQiu	2	4.53	-
N222737	2021	ShangQiu	3	5.6	-
N222738	2021	ShangQiu	1	4.34	-
N222738	2021	ShangQiu	2	4.09	-
N222738	2021	ShangQiu	3	3.27	-
N222739	2021	ShangQiu	1	4.93	-
N222739	2021	ShangQiu	2	-	-
N222739	2021	ShangQiu	3	4.81	-
N222740	2021	ShangQiu	1	6.31	-
N222740	2021	ShangQiu	2	6.46	-
N222740	2021	ShangQiu	3	4.47	-
N222741	2021	ShangQiu	1	4.04	-
N222741	2021	ShangQiu	2	5.24	-
N222741	2021	ShangQiu	3	6.16	-
N222742	2021	ShangQiu	1	4.07	-
N222742	2021	ShangQiu	2	5.16	-
N222742	2021	ShangQiu	3	6.34	-
N222743	2021	ShangQiu	1	4.84	-
N222743	2021	ShangQiu	2	7.26	-
N222743	2021	ShangQiu	3	6.31	-
N222744	2021	ShangQiu	1	6.16	-
N222744	2021	ShangQiu	2	5.24	-
N222744	2021	ShangQiu	3	4.91	-
N222745	2021	ShangQiu	1	7.37	-
N222745	2021	ShangQiu	2	7.22	-
N222745	2021	ShangQiu	3	6.15	-
N222746	2021	ShangQiu	1	3.58	-
N222746	2021	ShangQiu	2	2.99	-
N222746	2021	ShangQiu	3	3.66	-
N222747	2021	ShangQiu	1	5.19	-
N222747	2021	ShangQiu	2	-	-
N222747	2021	ShangQiu	3	-	-
N222748	2021	ShangQiu	1	4.18	-
N222748	2021	ShangQiu	2	4.77	-
N222748	2021	ShangQiu	3	4.72	-

Line	Year	Location	Replication	SD (mm)	BD (mm)
N222749	2021	ShangQiu	1	-	-
N222749	2021	ShangQiu	2	-	-
N222749	2021	ShangQiu	3	-	-
N222750	2021	ShangQiu	1	2.86	-
N222750	2021	ShangQiu	2	3.27	-
N222750	2021	ShangQiu	3	2.99	-
N222751	2021	ShangQiu	1	4.11	-
N222751	2021	ShangQiu	2	5.6	-
N222751	2021	ShangQiu	3	-	-
N222752	2021	ShangQiu	1	3.39	-
N222752	2021	ShangQiu	2	7.05	-
N222752	2021	ShangQiu	3	6.03	-
N222753	2021	ShangQiu	1	7.69	-
N222753	2021	ShangQiu	2	6.07	-
N222753	2021	ShangQiu	3	6.49	-
N222754	2021	ShangQiu	1	3.22	-
N222754	2021	ShangQiu	2	-	-
N222754	2021	ShangQiu	3	-	-
N222755	2021	ShangQiu	1	5.9	-
N222755	2021	ShangQiu	2	5.17	-
N222755	2021	ShangQiu	3	4.78	-
N222756	2021	ShangQiu	1	8.42	-
N222756	2021	ShangQiu	2	6.51	-
N222756	2021	ShangQiu	3	6.72	-
N222757	2021	ShangQiu	1	9.34	-
N222757	2021	ShangQiu	2	8.4	-
N222757	2021	ShangQiu	3	8.77	-
N222759	2021	ShangQiu	1	6.03	-
N222759	2021	ShangQiu	2	4.57	-
N222759	2021	ShangQiu	3	6.06	-
N222760	2021	ShangQiu	1	5.84	-
N222760	2021	ShangQiu	2	7.23	-
N222760	2021	ShangQiu	3	6.84	-
N222761	2021	ShangQiu	1	4.13	-
N222761	2021	ShangQiu	2	-	-
N222761	2021	ShangQiu	3	4.29	-
N222762	2021	ShangQiu	1	5.01	-
N222762	2021	ShangQiu	2	7.07	-
N222762	2021	ShangQiu	3	6.46	-
N222763	2021	ShangQiu	1	4.67	-
N222763	2021	ShangQiu	2	4.77	-
N222763	2021	ShangQiu	3	4.52	-
N222764	2021	ShangQiu	1	5.26	-
N222764	2021	ShangQiu	2	4.54	-
N222764	2021	ShangQiu	3	4.67	-

Line	Year	Location	Replication	SD (mm)	BD (mm)
N222765	2021	ShangQiu	1	5.84	-
N222765	2021	ShangQiu	2	5.38	-
N222765	2021	ShangQiu	3	3.19	-
N222766	2021	ShangQiu	1	6.9	-
N222766	2021	ShangQiu	2	4.39	-
N222766	2021	ShangQiu	3	4.87	-
N222767	2021	ShangQiu	1	7.1	-
N222767	2021	ShangQiu	2	6.32	-
N222767	2021	ShangQiu	3	5.32	-
N222768	2021	ShangQiu	1	5.02	-
N222768	2021	ShangQiu	2	4.6	-
N222768	2021	ShangQiu	3	2.53	-
N222769	2021	ShangQiu	1	3.65	-
N222769	2021	ShangQiu	2	5.17	-
N222769	2021	ShangQiu	3	-	-
N222770	2021	ShangQiu	1	5.21	-
N222770	2021	ShangQiu	2	3.95	-
N222770	2021	ShangQiu	3	2.73	-
N222771	2021	ShangQiu	1	6.07	-
N222771	2021	ShangQiu	2	4.67	-
N222771	2021	ShangQiu	3	4.96	-
N222772	2021	ShangQiu	1	4.78	-
N222772	2021	ShangQiu	2	6.09	-
N222772	2021	ShangQiu	3	5.16	-
N222773	2021	ShangQiu	1	3.92	-
N222773	2021	ShangQiu	2	3.04	-
N222773	2021	ShangQiu	3	4.26	-
N222774	2021	ShangQiu	1	3.74	-
N222774	2021	ShangQiu	2	5.23	-
N222774	2021	ShangQiu	3	5.49	-
N222775	2021	ShangQiu	1	4.31	-
N222775	2021	ShangQiu	2	3.46	-
N222775	2021	ShangQiu	3	5.65	-
N222776	2021	ShangQiu	1	3.48	-
N222776	2021	ShangQiu	2	4.76	-
N222776	2021	ShangQiu	3	5.65	-
N222777	2021	ShangQiu	1	-	-
N222777	2021	ShangQiu	2	-	-
N222777	2021	ShangQiu	3	-	-
N222778	2021	ShangQiu	1	-	-
N222778	2021	ShangQiu	2	-	-
N222778	2021	ShangQiu	3	-	-
N222779	2021	ShangQiu	1	6.56	-
N222779	2021	ShangQiu	2	5.67	-
N222779	2021	ShangQiu	3	4.69	-

Line	Year	Location	Replication	SD (mm)	BD (mm)
N222780	2021	ShangQiu	1	6.17	-
N222780	2021	ShangQiu	2	7.07	-
N222780	2021	ShangQiu	3	8.89	-
N222781	2021	ShangQiu	1	5.87	-
N222781	2021	ShangQiu	2	6.38	-
N222781	2021	ShangQiu	3	6.92	-
N222782	2021	ShangQiu	1	8.85	-
N222782	2021	ShangQiu	2	6.48	-
N222782	2021	ShangQiu	3	7.62	-
N222783	2021	ShangQiu	1	5.83	-
N222783	2021	ShangQiu	2	6.63	-
N222783	2021	ShangQiu	3	5.62	-
N222784	2021	ShangQiu	1	3.61	-
N222784	2021	ShangQiu	2	6.9	-
N222784	2021	ShangQiu	3	4.57	-
N222785	2021	ShangQiu	1	4.15	-
N222785	2021	ShangQiu	2	4.22	-
N222785	2021	ShangQiu	3	5.11	-
N222786	2021	ShangQiu	1	5.65	-
N222786	2021	ShangQiu	2	6.28	-
N222786	2021	ShangQiu	3	4.93	-
N222787	2021	ShangQiu	1	5.55	-
N222787	2021	ShangQiu	2	5.54	-
N222787	2021	ShangQiu	3	4.29	-
N222788	2021	ShangQiu	1	6.38	-
N222788	2021	ShangQiu	2	6.08	-
N222788	2021	ShangQiu	3	4	-
N222789	2021	ShangQiu	1	5.78	-
N222789	2021	ShangQiu	2	5.74	-
N222789	2021	ShangQiu	3	5.11	-
N222790	2021	ShangQiu	1	4.47	-
N222790	2021	ShangQiu	2	4.2	-
N222790	2021	ShangQiu	3	3.59	-
N222791	2021	ShangQiu	1	4.13	-
N222791	2021	ShangQiu	2	5.19	-
N222791	2021	ShangQiu	3	4.58	-
N222792	2021	ShangQiu	1	4.57	-
N222792	2021	ShangQiu	2	4.9	-
N222792	2021	ShangQiu	3	5.3	-
N222793	2021	ShangQiu	1	4.73	-
N222793	2021	ShangQiu	2	6.83	-
N222793	2021	ShangQiu	3	4.95	-
N222794	2021	ShangQiu	1	4.7	-
N222794	2021	ShangQiu	2	4.07	-
N222794	2021	ShangQiu	3	4.18	-

Line	Year	Location	Replication	SD (mm)	BD (mm)
N222795	2021	ShangQiu	1	6.61	-
N222795	2021	ShangQiu	2	7.09	-
N222795	2021	ShangQiu	3	6.54	-
N222796	2021	ShangQiu	1	3.25	-
N222796	2021	ShangQiu	2	3.37	-
N222796	2021	ShangQiu	3	3.77	-
N222797	2021	ShangQiu	1	4.12	-
N222797	2021	ShangQiu	2	3.82	-
N222797	2021	ShangQiu	3	4	-
N222798	2021	ShangQiu	1	5.79	-
N222798	2021	ShangQiu	2	5.54	-
N222798	2021	ShangQiu	3	5.9	-
N222799	2021	ShangQiu	1	7.33	-
N222799	2021	ShangQiu	2	7.71	-
N222799	2021	ShangQiu	3	6.87	-
N222800	2021	ShangQiu	1	5.25	-
N222800	2021	ShangQiu	2	3.65	-
N222800	2021	ShangQiu	3	4.96	-
N222801	2021	ShangQiu	1	8.89	-
N222801	2021	ShangQiu	2	9.67	-
N222801	2021	ShangQiu	3	9.14	-
N222802	2021	ShangQiu	1	5.12	-
N222802	2021	ShangQiu	2	4.16	-
N222802	2021	ShangQiu	3	3.94	-
N222803	2021	ShangQiu	1	5.34	-
N222803	2021	ShangQiu	2	5.09	-
N222803	2021	ShangQiu	3	-	-
N222804	2021	ShangQiu	1	3.32	-
N222804	2021	ShangQiu	2	4.92	-
N222804	2021	ShangQiu	3	3.53	-
N222805	2021	ShangQiu	1	4.48	-
N222805	2021	ShangQiu	2	4.65	-
N222805	2021	ShangQiu	3	4.08	-
N222806	2021	ShangQiu	1	6.15	-
N222806	2021	ShangQiu	2	5.35	-
N222806	2021	ShangQiu	3	4.68	-
N222807	2021	ShangQiu	1	5.47	-
N222807	2021	ShangQiu	2	5.58	-
N222807	2021	ShangQiu	3	5.11	-
N222808	2021	ShangQiu	1	6.62	-
N222808	2021	ShangQiu	2	7.63	-
N222808	2021	ShangQiu	3	9.22	-
N222809	2021	ShangQiu	1	3.91	-
N222809	2021	ShangQiu	2	4.65	-
N222809	2021	ShangQiu	3	4.27	-

Line	Year	Location	Replication	SD (mm)	BD (mm)
N222810	2021	ShangQiu	1	5.53	-
N222810	2021	ShangQiu	2	4.29	-
N222810	2021	ShangQiu	3	5.52	-
N222811	2021	ShangQiu	1	4.23	-
N222811	2021	ShangQiu	2	5	-
N222811	2021	ShangQiu	3	4.94	-
N222812	2021	ShangQiu	1	3.7	-
N222812	2021	ShangQiu	2	5.16	-
N222812	2021	ShangQiu	3	4.19	-
N222813	2021	ShangQiu	1	6.15	-
N222813	2021	ShangQiu	2	6.92	-
N222813	2021	ShangQiu	3	-	-
N222814	2021	ShangQiu	1	-	-
N222814	2021	ShangQiu	2	5.21	-
N222814	2021	ShangQiu	3	5.37	-
N222815	2021	ShangQiu	1	5.47	-
N222815	2021	ShangQiu	2	6.4	-
N222815	2021	ShangQiu	3	7.98	-
N222816	2021	ShangQiu	1	9.99	-
N222816	2021	ShangQiu	2	6.73	-
N222816	2021	ShangQiu	3	7.77	-
N222817	2021	ShangQiu	1	6.54	-
N222817	2021	ShangQiu	2	7.46	-
N222817	2021	ShangQiu	3	6.28	-
N222818	2021	ShangQiu	1	7.47	-
N222818	2021	ShangQiu	2	7.19	-
N222818	2021	ShangQiu	3	7.06	-
N222819	2021	ShangQiu	1	8.79	-
N222819	2021	ShangQiu	2	6.94	-
N222819	2021	ShangQiu	3	8.14	-
N222820	2021	ShangQiu	1	3.64	-
N222820	2021	ShangQiu	2	4.32	-
N222820	2021	ShangQiu	3	3.54	-
N222821	2021	ShangQiu	1	4.54	-
N222821	2021	ShangQiu	2	3.65	-
N222821	2021	ShangQiu	3	5.03	-
N222822	2021	ShangQiu	1	7.2	-
N222822	2021	ShangQiu	2	7.75	-
N222822	2021	ShangQiu	3	7.34	-
N222823	2021	ShangQiu	1	6.13	-
N222823	2021	ShangQiu	2	5.54	-
N222823	2021	ShangQiu	3	6.03	-
N222824	2021	ShangQiu	1	3.82	-
N222824	2021	ShangQiu	2	4.05	-
N222824	2021	ShangQiu	3	4	-

Line	Year	Location	Replication	SD (mm)	BD (mm)
N222825	2021	ShangQiu	1	4.82	-
N222825	2021	ShangQiu	2	4.17	-
N222825	2021	ShangQiu	3	5.07	-
N222826	2021	ShangQiu	1	5.07	-
N222826	2021	ShangQiu	2	5.41	-
N222826	2021	ShangQiu	3	4.9	-
N222827	2021	ShangQiu	1	4.76	-
N222827	2021	ShangQiu	2	3.48	-
N222827	2021	ShangQiu	3	5.25	-
N222828	2021	ShangQiu	1	4.78	-
N222828	2021	ShangQiu	2	5.02	-
N222828	2021	ShangQiu	3	4.58	-
N222829	2021	ShangQiu	1	4.98	-
N222829	2021	ShangQiu	2	4.86	-
N222829	2021	ShangQiu	3	5.41	-
N222830	2021	ShangQiu	1	4.99	-
N222830	2021	ShangQiu	2	5.55	-
N222830	2021	ShangQiu	3	4.58	-
N222831	2021	ShangQiu	1	6.21	-
N222831	2021	ShangQiu	2	4.58	-
N222831	2021	ShangQiu	3	5.48	-
N222832	2021	ShangQiu	1	4.46	-
N222832	2021	ShangQiu	2	5.76	-
N222832	2021	ShangQiu	3	9.93	-
N222833	2021	ShangQiu	1	9.44	-
N222833	2021	ShangQiu	2	8.77	-
N222833	2021	ShangQiu	3	7.41	-
N222834	2021	ShangQiu	1	4.39	-
N222834	2021	ShangQiu	2	4.75	-
N222834	2021	ShangQiu	3	3.53	-
N222835	2021	ShangQiu	1	3.72	-
N222835	2021	ShangQiu	2	9.42	-
N222835	2021	ShangQiu	3	9.54	-
N222836	2021	ShangQiu	1	4.47	-
N222836	2021	ShangQiu	2	5.3	-
N222836	2021	ShangQiu	3	4.07	-
N222837	2021	ShangQiu	1	7.01	-
N222837	2021	ShangQiu	2	6.82	-
N222837	2021	ShangQiu	3	7.75	-
N222838	2021	ShangQiu	1	4.95	-
N222838	2021	ShangQiu	2	6.51	-
N222838	2021	ShangQiu	3	7.3	-
N222839	2021	ShangQiu	1	5.61	-
N222839	2021	ShangQiu	2	5.12	-
N222839	2021	ShangQiu	3	5.55	-

Line	Year	Location	Replication	SD (mm)	BD (mm)
N222840	2021	ShangQiu	1	5.75	-
N222840	2021	ShangQiu	2	6.09	-
N222840	2021	ShangQiu	3	6.9	-
N222841	2021	ShangQiu	1	3.05	-
N222841	2021	ShangQiu	2	3.88	-
N222841	2021	ShangQiu	3	4.33	-
N222842	2021	ShangQiu	1	9.84	-
N222842	2021	ShangQiu	2	-	-
N222842	2021	ShangQiu	3	8.52	-
N222843	2021	ShangQiu	1	3.93	-
N222843	2021	ShangQiu	2	5.17	-
N222843	2021	ShangQiu	3	6.03	-
N222844	2021	ShangQiu	1	7.16	-
N222844	2021	ShangQiu	2	6.8	-
N222844	2021	ShangQiu	3	6.33	-
N222845	2021	ShangQiu	1	5.11	-
N222845	2021	ShangQiu	2	3.81	-
N222845	2021	ShangQiu	3	4.34	-
N222846	2021	ShangQiu	1	5.68	-
N222846	2021	ShangQiu	2	7.5	-
N222846	2021	ShangQiu	3	5.31	-
N222847	2021	ShangQiu	1	6.17	-
N222847	2021	ShangQiu	2	5.06	-
N222847	2021	ShangQiu	3	5.6	-
N222848	2021	ShangQiu	1	6.17	-
N222848	2021	ShangQiu	2	-	-
N222848	2021	ShangQiu	3	6.47	-
N222849	2021	ShangQiu	1	7.02	-
N222849	2021	ShangQiu	2	6.35	-
N222849	2021	ShangQiu	3	5.49	-
N222850	2021	ShangQiu	1	10.06	-
N222850	2021	ShangQiu	2	12.06	-
N222850	2021	ShangQiu	3	12.35	-
N222851	2021	ShangQiu	1	4.42	-
N222851	2021	ShangQiu	2	5.81	-
N222851	2021	ShangQiu	3	5.36	-
N222852	2021	ShangQiu	1	4.63	-
N222852	2021	ShangQiu	2	3.33	-
N222852	2021	ShangQiu	3	5.34	-
N222853	2021	ShangQiu	1	6.09	-
N222853	2021	ShangQiu	2	5.21	-
N222853	2021	ShangQiu	3	3.7	-
N222854	2021	ShangQiu	1	7.32	-
N222854	2021	ShangQiu	2	4.08	-
N222854	2021	ShangQiu	3	4.66	-

Line	Year	Location	Replication	SD (mm)	BD (mm)
ZD41	2021	ShangQiu	1	7.18	-
ZD41	2021	ShangQiu	2	10.28	-
ZD41	2021	ShangQiu	3	9.69	-
ZYD02878	2021	ShangQiu	1	3.42	-
ZYD02878	2021	ShangQiu	2	3.44	-
ZYD02878	2021	ShangQiu	3	3.4	-

SD: stem diameter; BD: branch diameter; JZ: Jingzhou; SY: Sanya; SQ: Shangqiu

Supplementary Table S2. P value of Pearson correlation among SD and BD in different environments

Trait	Environments	SD				BD			SD.BLUE	SD.mean
		2019JZ	2019SY	2020JZ	2021SQ	2019JZ	2019SY	2020JZ		
SD	2019JZ	/								
	2019SY	1.64E-05	/							
	2020JZ	0	1.37E-04	/						
	2021SQ	6.59E-12	3.17E-13	0	/					
BD	2019JZ	0	5.18E-06	0	4.44E-16	/				
	2019SY	2.62E-02	0	3.96E-03	9.68E-06	1.51E-04	/			
	2020JZ	4.44E-16	1.85E-06	0	0	0	1.48E-06	/		
BD.BLUE		0	0	0	0	0	0	0	0	0
BD.mean		0	0	0	0	0	0	0	0	0

SD: stem diameter; BD: branch diameter; JZ: Jingzhou; SY: Sanya; SQ: Shangqiu; BLUE: best linear unbiased estimate.

Supplementary Table S3. QTL mapping of SD and BD phenotypic in different environments, BLUE and mean using three methods

Serial No.	Trait	Environment	QTL	Chromosome	Start	End	PVE(%)	Add	P-value	LOD	Methods
1	SD	2019JZ	*qSD2-1*	2	27,201,003	27,243,883	3.67	/	0.0027	/	RTM-GWAS
2	SD	2019JZ	*qSD2-2*	2	27,243,883	27,381,884	2.06	/	0.0042	/	RTM-GWAS
3	SD	2019JZ	*qSD5-1*	5	7,858,918	7,923,552	2.66	/	0.0068	/	RTM-GWAS
4	SD	2019JZ	*qSD10-1*	10	5,347,014	6,926,536	6.11	/	0.0001	/	RTM-GWAS
5	SD	2019JZ	*qSD10-2*	10	41,181,895	41,231,103	8.03	/	0.0000	/	RTM-GWAS
6	SD	2019JZ	*qSD10-3*	10	45,475,505	45,622,801	2.98	/	0.0082	/	RTM-GWAS
7	SD	2019JZ	*qSD11-1*	11	26,556,902	26,591,373	3.10	/	0.0049	/	RTM-GWAS
8	SD	2019JZ	*qSD12-1*	12	10,621,355	11,150,969	2.99	/	0.0061	/	RTM-GWAS
9	SD	2019JZ	*qSD17-1*	17	2,526,930	2,664,000	5.01	/	0.0030	/	RTM-GWAS
10	SD	2019JZ	*qSD18-1*	18	19,303,346	19,430,527	1.66	/	0.0086	/	RTM-GWAS
11	SD	2019JZ	*qSD18-2*	18	55,674,297	55,991,102	5.52	/	0.0002	/	RTM-GWAS
12	SD	2019JZ	*qSD19-1*	19	20,727,913	23,101,391	3.48	/	0.0030	/	RTM-GWAS
13	SD	2019JZ	*qSD20-1*	20	38,842,806	38,875,325	3.09	/	0.0043	/	RTM-GWAS
14	SD	2019JZ	*qSD20-2*	20	44,666,415	44,746,133	2.38	/	0.0081	/	RTM-GWAS
15	SD	2019SY	*qSD1-1*	1	49,347,541	49,351,707	4.52	/	0.0009	/	RTM-GWAS
16	SD	2019SY	*qSD2-3*	2	43,703,500	43,710,097	5.01	/	0.0009	/	RTM-GWAS
17	SD	2019SY	*qSD3-1*	3	36,518,347	36,708,811	4.90	/	0.0008	/	RTM-GWAS
18	SD	2019SY	*qSD7-1*	7	14,012,455	14,094,620	4.14	/	0.0010	/	RTM-GWAS
19	SD	2019SY	*qSD8-2*	8	46,395,913	46,518,573	13.64	/	0.0000	/	RTM-GWAS
20	SD	2019SY	*qSD8-1*	8	16,613,265	16,660,545	4.12	/	0.0013	/	RTM-GWAS
21	SD	2019SY	*qSD15-1*	15	6,321,085	6,430,985	8.06	/	0.0000	/	RTM-GWAS
22	SD	2019SY	*qSD17-2*	17	37,778,039	37,853,866	4.47	/	0.0012	/	RTM-GWAS

Serial No.	Trait	Environment	QTL	Chromosome	Start	End	PVE(%)	Add	P-value	LOD	Methods
23	SD	2020JZ	*qSD2-4*	2	25,817,205	25,949,494	1.83	/	0.0027	/	RTM-GWAS
24	SD	2020JZ	*qSD2-5*	2	41,501,976	41,519,661	3.00	/	0.0003	/	RTM-GWAS
25	SD	2020JZ	*qSD2-6*	2	47,621,618	47,712,802	5.03	/	0.0000	/	RTM-GWAS
26	SD	2020JZ	*qSD7-2*	7	0	6,382,366	2.43	/	0.0019	/	RTM-GWAS
27	SD	2020JZ	*qSD7-3*	7	19,828,550	19,839,096	1.66	/	0.0036	/	RTM-GWAS
28	SD	2020JZ	*qSD8-3*	8	1,676,370	1,812,439	1.53	/	0.0098	/	RTM-GWAS
29	SD	2020JZ	*qSD8-4*	8	5,980,273	6,126,734	5.75	/	0.0000	/	RTM-GWAS
30	SD	2020JZ	*qSD8-5*	8	46,168,008	46,239,110	3.68	/	0.0002	/	RTM-GWAS
31	SD	2020JZ	*qSD10-4*	10	45,120,118	45,257,940	19.61	/	0.0000	/	RTM-GWAS
32	SD	2020JZ	*qSD11-2*	11	10,779,406	10,904,384	5.72	/	0.0000	/	RTM-GWAS
33	SD	2020JZ	*qSD15-2*	15	11,883,830	11,934,841	3.06	/	0.0018	/	RTM-GWAS
34	SD	2020JZ	*qSD18-3*	18	15,940,594	16,188,577	3.38	/	0.0001	/	RTM-GWAS
35	SD	2020JZ	*qSD18-4*	18	48,367,672	49,013,195	4.07	/	0.0001	/	RTM-GWAS
36	SD	2020JZ	*qSD19-2*	19	2,534,128	2,667,930	2.87	/	0.0038	/	RTM-GWAS
37	SD	2020JZ	*qSD20-3*	20	37,208,897	37,335,219	2.56	/	0.0028	/	RTM-GWAS
38	SD	2020JZ	*qSD20-4*	20	43,990,641	44,085,914	2.01	/	0.0028	/	RTM-GWAS
39	SD	2021SQ	*qSD2-7*	2	25,949,494	26,416,236	3.06	/	0.0009	/	RTM-GWAS
40	SD	2021SQ	*qSD4-1*	4	50,667,694	50,690,238	2.83	/	0.0092	/	RTM-GWAS
41	SD	2021SQ	*qSD8-6*	8	9,260,312	9,435,430	4.98	/	0.0014	/	RTM-GWAS
42	SD	2021SQ	*qSD8-7*	8	36,400,556	37,040,512	7.73	/	0.0000	/	RTM-GWAS
43	SD	2021SQ	*qSD8-8*	8	45,911,551	45,913,342	5.38	/	0.0014	/	RTM-GWAS
44	SD	2021SQ	*qSD9-1*	9	4,097,186	4,190,365	4.08	/	0.0057	/	RTM-GWAS
45	SD	2021SQ	*qSD9-2*	9	19,082,826	19,301,463	2.57	/	0.0076	/	RTM-GWAS

Serial No.	Trait	Environment	QTL	Chromosome	Start	End	PVE(%)	Add	P-value	LOD	Methods
46	SD	2021SQ	*qSD11-3*	11	31,936,739	31,953,512	3.01	/	0.0019	/	RTM-GWAS
47	SD	2021SQ	*qSD13-1*	13	28,337,047	28,710,672	5.17	/	0.0033	/	RTM-GWAS
48	SD	2021SQ	*qSD13-2*	13	31,799,883	31,848,373	3.79	/	0.0018	/	RTM-GWAS
49	SD	2021SQ	*qSD15-3*	15	6,982,474	7,021,640	6.33	/	0.0069	/	RTM-GWAS
50	SD	2021SQ	*qSD20-5*	20	44,404,567	44,407,089	3.66	/	0.0039	/	RTM-GWAS
51	SD	BLUE	*qSD2-8*	2	43,607,379	43,607,379	5.94	/	0.0000	/	RTM-GWAS
52	SD	BLUE	*qSD3-2*	3	37,475,674	37,475,674	2.52	/	0.0025	/	RTM-GWAS
53	SD	BLUE	*qSD5-2*	5	23,236,127	23,236,127	2.34	/	0.0032	/	RTM-GWAS
54	SD	BLUE	*qSD5-3*	5	24,803,769	24,803,769	1.72	/	0.0050	/	RTM-GWAS
55	SD	BLUE	*qSD8-9*	8	39,878,906	39,917,603	3.46	/	0.0005	/	RTM-GWAS
56	SD	BLUE	*qSD8-10*	8	46,364,044	46,364,044	8.52	/	0.0000	/	RTM-GWAS
57	SD	BLUE	*qSD9-3*	9	1,734,905	1,880,548	2.72	/	0.0032	/	RTM-GWAS
58	SD	BLUE	*qSD9-4*	9	28,312,724	28,312,724	1.69	/	0.0064	/	RTM-GWAS
59	SD	BLUE	*qSD10-5*	10	45,120,118	45,257,940	17.39	/	0.0000	/	RTM-GWAS
60	SD	BLUE	*qSD11-4*	11	11,043,519	11,043,519	4.46	/	0.0001	/	RTM-GWAS
61	SD	BLUE	*qSD13-3*	13	43,447,381	43,447,381	2.07	/	0.0038	/	RTM-GWAS
62	SD	BLUE	*qSD13-4*	13	45,152,104	45,152,104	1.76	/	0.0077	/	RTM-GWAS
63	SD	BLUE	*qSD15-4*	15	11,778,040	11,883,830	2.25	/	0.0028	/	RTM-GWAS
64	SD	BLUE	*qSD15-5*	15	5,532,206	5,532,206	4.63	/	0.0001	/	RTM-GWAS
65	SD	BLUE	*qSD16-1*	16	30,566,971	30,566,971	1.70	/	0.0070	/	RTM-GWAS
66	SD	BLUE	*qSD19-3*	19	4,506,176	4,506,176	1.82	/	0.0058	/	RTM-GWAS
67	SD	BLUE	*qSD20-6*	20	37,811,887	37,811,887	4.15	/	0.0002	/	RTM-GWAS
68	SD	BLUE	*qSD20-7*	20	44,666,415	44,666,415	2.95	/	0.0011	/	RTM-GWAS

Serial No.	Trait	Environment	QTL	Chromosome	Start	End	PVE(%)	Add	P-value	LOD	Methods
69	SD	mean	*qSD2-10*	2	41,501,976	41,519,661	1.46	/	0.0084	/	RTM-GWAS
70	SD	mean	*qSD2-9*	2	43,607,379	43,703,500	7.12	/	0.0000	/	RTM-GWAS
71	SD	mean	*qSD5-5*	5	24,803,769	27,738,758	1.72	/	0.0042	/	RTM-GWAS
72	SD	mean	*qSD5-4*	5	7,858,918	7,923,552	3.58	/	0.0003	/	RTM-GWAS
73	SD	mean	*qSD8-12*	8	14,694,018	14,812,948	2.31	/	0.0018	/	RTM-GWAS
74	SD	mean	*qSD8-13*	8	39,697,455	39,878,906	2.77	/	0.0004	/	RTM-GWAS
75	SD	mean	*qSD8-11*	8	46,364,044	46,395,913	5.79	/	0.0000	/	RTM-GWAS
76	SD	mean	*qSD10-6*	10	45,120,118	45,257,940	16.36	/	0.0000	/	RTM-GWAS
77	SD	mean	*qSD10-7*	10	41,520,177	41,675,137	3.77	/	0.0002	/	RTM-GWAS
78	SD	mean	*qSD10-8*	10	41,689,785	41,723,082	2.23	/	0.0010	/	RTM-GWAS
79	SD	mean	*qSD11-5*	11	11,043,519	11,092,389	4.67	/	0.0001	/	RTM-GWAS
80	SD	mean	*qSD13-7*	13	44,327,651	44,427,118	2.42	/	0.0008	/	RTM-GWAS
81	SD	mean	*qSD13-6*	13	37,145,616	37,175,077	3.25	/	0.0002	/	RTM-GWAS
82	SD	mean	*qSD13-5*	13	45,059,426	45,152,104	3.88	/	0.0003	/	RTM-GWAS
83	SD	mean	*qSD15-6*	15	6,889,315	6,982,474	4.00	/	0.0003	/	RTM-GWAS
84	SD	mean	*qSD18-5*	18	295,669	363,749	3.35	/	0.0003	/	RTM-GWAS
85	SD	mean	*qSD18-6*	18	48,309,457	48,367,672	2.83	/	0.0008	/	RTM-GWAS
86	SD	mean	*qSD20-8*	20	37,335,219	37,344,280	1.41	/	0.0087	/	RTM-GWAS
87	BD	2019JZ	*qBD2-1*	2	39,493,768	39,703,135	4.27	/	0.0006	/	RTM-GWAS
88	BD	2019JZ	*qBD5-1*	5	7,858,918	7,923,552	4.46	/	0.0002	/	RTM-GWAS
89	BD	2019JZ	*qBD8-1*	8	46,395,913	46,518,573	5.77	/	0.0001	/	RTM-GWAS
90	BD	2019JZ	*qBD9-1*	9	3,785,101	3,911,791	1.91	/	0.0043	/	RTM-GWAS
91	BD	2019JZ	*qBD9-2*	9	3,911,791	3,976,497	3.91	/	0.0007	/	RTM-GWAS

Serial No.	Trait	Environment	QTL	Chromosome	Start	End	PVE(%)	Add	P-value	LOD	Methods
92	BD	2019JZ	*qBD10-1*	10	45,475,505	45,622,801	17.46	/	0.0000	/	RTM-GWAS
93	BD	2019JZ	*qBD11-1*	11	8,739,403	9,363,712	2.60	/	0.0026	/	RTM-GWAS
94	BD	2019JZ	*qBD11-2*	11	10,609,377	10,744,281	5.15	/	0.0002	/	RTM-GWAS
95	BD	2019JZ	*qBD11-3*	11	10,904,384	11,043,519	2.29	/	0.0061	/	RTM-GWAS
96	BD	2019JZ	*qBD13-1*	13	37,025,399	37,145,616	4.56	/	0.0001	/	RTM-GWAS
97	BD	2019JZ	*qBD13-2*	13	38,041,203	38,067,451	4.01	/	0.0007	/	RTM-GWAS
98	BD	2019JZ	*qBD17-1*	17	9,731,268	9,764,051	2.13	/	0.0097	/	RTM-GWAS
99	BD	2019SY	*qBD3-1*	3	6,737,862	6,776,986	4.01	/	0.0013	/	RTM-GWAS
100	BD	2019SY	*qBD4-1*	4	48,403,516	48,519,846	3.46	/	0.0022	/	RTM-GWAS
101	BD	2019SY	*qBD5-2*	5	2,215,956	2,385,119	5.43	/	0.0003	/	RTM-GWAS
102	BD	2019SY	*qBD7-1*	7	18,151,571	18,335,168	2.74	/	0.0046	/	RTM-GWAS
103	BD	2019SY	*qBD7-2*	7	39,446,389	39,484,113	2.37	/	0.0075	/	RTM-GWAS
104	BD	2019SY	*qBD8-2*	8	17,599,584	17,848,472	5.40	/	0.0002	/	RTM-GWAS
105	BD	2019SY	*qBD8-3*	8	47,505,768	47,525,385	9.31	/	0.0000	/	RTM-GWAS
106	BD	2019SY	*qBD9-3*	9	37,983,058	38,139,379	3.80	/	0.0010	/	RTM-GWAS
107	BD	2019SY	*qBD10-2*	10	46,072,021	46,210,329	6.66	/	0.0001	/	RTM-GWAS
108	BD	2019SY	*qBD10-3*	10	47,076,496	47,110,242	2.44	/	0.0073	/	RTM-GWAS
109	BD	2019SY	*qBD11-4*	11	24,983,624	25,055,183	10.49	/	0.0000	/	RTM-GWAS
110	BD	2019SY	*qBD15-1*	15	7,247,739	7,353,069	3.59	/	0.0021	/	RTM-GWAS
111	BD	2019SY	*qBD19-1*	19	37,416,630	37,729,056	4.20	/	0.0003	/	RTM-GWAS
112	BD	2020JZ	*qBD2-2*	2	6,541,153	6,565,935	3.23	/	0.0007	/	RTM-GWAS
113	BD	2020JZ	*qBD2-3*	2	46,277,482	46,336,665	3.66	/	0.0006	/	RTM-GWAS
114	BD	2020JZ	*qBD3-2*	3	282,097	345,265	2.21	/	0.0039	/	RTM-GWAS

Serial No.	Trait	Environment	QTL	Chromosome	Start	End	PVE(%)	Add	P-value	LOD	Methods
115	BD	2020JZ	*qBD4-2*	4	13,690,192	14,592,070	5.16	/	0.0001	/	RTM-GWAS
116	BD	2020JZ	*qBD4-3*	4	19,000,528	19,935,526	2.39	/	0.0037	/	RTM-GWAS
117	BD	2020JZ	*qBD4-4*	4	33,900,234	34,005,811	3.17	/	0.0005	/	RTM-GWAS
118	BD	2020JZ	*qBD5-3*	5	0	584,541	3.31	/	0.0007	/	RTM-GWAS
119	BD	2020JZ	*qBD6-1*	6	11,037,943	11,108,003	1.98	/	0.0047	/	RTM-GWAS
120	BD	2020JZ	*qBD8-4*	8	6,917,898	6,937,219	7.09	/	0.0000	/	RTM-GWAS
121	BD	2020JZ	*qBD8-5*	8	47,021,800	47,061,716	2.93	/	0.0021	/	RTM-GWAS
122	BD	2020JZ	*qBD10-5*	10	45,120,118	45,257,940	15.11	/	0.0000	/	RTM-GWAS
123	BD	2020JZ	*qBD10-4*	10	39,592,113	39,630,566	2.11	/	0.0038	/	RTM-GWAS
124	BD	2020JZ	*qBD11-5*	11	10,779,406	10,904,384	7.24	/	0.0000	/	RTM-GWAS
125	BD	2020JZ	*qBD15-2*	15	12,253,558	12,304,443	4.35	/	0.0003	/	RTM-GWAS
126	BD	2020JZ	*qBD16-1*	16	30,566,971	30,623,866	1.74	/	0.0080	/	RTM-GWAS
127	BD	2020JZ	*qBD18-1*	18	15,940,594	16,188,577	4.02	/	0.0001	/	RTM-GWAS
128	BD	2020JZ	*qBD20-1*	20	1,855,970	2,013,720	1.80	/	0.0097	/	RTM-GWAS
129	BD	BLUE	*qBD4-5*	4	52,044,264	52,261,891	2.98	/	0.0031	/	RTM-GWAS
130	BD	BLUE	*qBD7-3*	7	10,919,362	11,202,690	2.78	/	0.0036	/	RTM-GWAS
131	BD	BLUE	*qBD8-6*	8	16,111,014	16,210,000	2.52	/	0.0043	/	RTM-GWAS
132	BD	BLUE	*qBD8-7*	8	46,945,460	47,021,800	10.48	/	0.0000	/	RTM-GWAS
133	BD	BLUE	*qBD10-6*	10	1,495,432	1,498,141	5.06	/	0.0011	/	RTM-GWAS
134	BD	BLUE	*qBD10-7*	10	46,053,381	46,072,021	15.14	/	0.0020	/	RTM-GWAS
135	BD	BLUE	*qBD11-6*	11	24,823,012	24,983,624	6.41	/	0.0003	/	RTM-GWAS
136	BD	BLUE	*qBD11-7*	11	31,107,187	31,154,196	2.42	/	0.0038	/	RTM-GWAS
137	BD	BLUE	*qBD12-1*	12	5,475,077	5,724,257	2.06	/	0.0072	/	RTM-GWAS

Serial No.	Trait	Environment	QTL	Chromosome	Start	End	PVE(%)	Add	P-value	LOD	Methods
138	BD	BLUE	*qBD12-2*	12	36,269,441	36,402,072	3.25	/	0.0036	/	RTM-GWAS
139	BD	BLUE	*qBD15-3*	15	10,827,709	10,860,771	8.62	/	0.0001	/	RTM-GWAS
140	BD	BLUE	*qBD19-2*	19	38,058,638	38,188,500	2.64	/	0.0054	/	RTM-GWAS
141	BD	BLUE	*qBD20-2*	20	37,526,276	37,600,191	4.08	/	0.0021	/	RTM-GWAS
142	BD	mean	*qBD2-4*	2	25,817,205	25,949,494	3.44	/	0.0014	/	RTM-GWAS
143	BD	mean	*qBD3-3*	3	13,115,549	13,132,697	5.21	/	0.0001	/	RTM-GWAS
144	BD	mean	*qBD8-8*	8	46,395,913	46,518,573	10.28	/	0.0000	/	RTM-GWAS
145	BD	mean	*qBD9-4*	9	45,059,701	45,072,261	2.82	/	0.0028	/	RTM-GWAS
146	BD	mean	*qBD10-9*	10	1,687,575	1,721,654	3.72	/	0.0010	/	RTM-GWAS
147	BD	mean	*qBD10-8*	10	45,658,686	45,697,530	21.68	/	0.0000	/	RTM-GWAS
148	BD	mean	*qBD11-8*	11	25,264,762	25,439,066	5.67	/	0.0001	/	RTM-GWAS
149	BD	mean	*qBD12-3*	12	5,475,077	5,724,257	2.21	/	0.0088	/	RTM-GWAS
150	BD	mean	*qBD13-3*	13	37,145,616	37,175,077	2.51	/	0.0062	/	RTM-GWAS
151	BD	mean	*qBD15-4*	15	8,539,141	8,649,553	5.78	/	0.0001	/	RTM-GWAS
152	BD	mean	*qBD18-2*	18	48,179,723	48,200,966	3.00	/	0.0027	/	RTM-GWAS
153	SD	2019JZ	*qSD1-2*	1	53,117,378	53,159,107	2.25	-0.24	/	2.77	ICIM
154	SD	2019JZ	*qSD11-6*	11	11,043,519	11,099,081	4.76	-0.35	/	4.28	ICIM
155	SD	2019SY	*qSD1-3*	1	49,351,707	49,373,434	4.32	0.11	/	4.80	ICIM
156	SD	2019SY	*qSD2-11*	2	4,623,142	4,689,678	3.10	0.09	/	3.46	ICIM
157	SD	2019SY	*qSD2-12*	2	43,710,097	43,831,428	6.48	0.13	/	7.12	ICIM
158	SD	2019SY	*qSD7-4*	7	14,094,620	14,094,620	3.22	0.09	/	3.60	ICIM
159	SD	2019SY	*qSD8-14*	8	46,364,044	46,420,859	6.79	-0.13	/	7.40	ICIM
160	SD	2019SY	*qSD9-5*	9	36,249,643	36,545,817	3.40	0.09	/	3.77	ICIM

Serial No.	Trait	Environment	QTL	Chromosome	Start	End	PVE(%)	Add	P-value	LOD	Methods
161	SD	2019SY	*qSD11-7*	11	24,308,457	24,394,808	3.05	0.09	/	3.06	ICIM
162	SD	2019SY	*qSD12-2*	12	36,287,746	36,347,559	2.40	0.08	/	2.70	ICIM
163	SD	2019SY	*qSD13-8*	13	37,642,237	37,788,901	4.04	0.10	/	4.47	ICIM
164	SD	2019SY	*qSD15-7*	15	6,335,490	6,377,305	3.70	0.10	/	4.11	ICIM
165	SD	2020JZ	*qSD6-1*	6	44,555,867	44,964,228	2.64	0.22	/	4.44	ICIM
166	SD	2020JZ	*qSD8-15*	8	7,225,100	7,322,137	2.16	0.20	/	3.75	ICIM
167	SD	2020JZ	*qSD10-9*	10	45,243,194	45,322,107	15.96	0.55	/	24.09	ICIM
168	SD	2020JZ	*qSD18-7*	18	48,264,142	48,367,672	2.67	-0.23	/	4.54	ICIM
169	SD	2020JZ	*qSD20-9*	20	44,775,256	44,986,065	2.88	0.24	/	4.83	ICIM
170	SD	2021SQ	*qSD2-13*	2	4,898,292	5,026,152	6.93	0.27	/	5.55	ICIM
171	SD	BLUE	*qSD1-4*	1	48,601,855	48,679,456	6.01	0.19	/	9.52	ICIM
172	SD	BLUE	*qSD2-14*	2	43,411,761	43,527,880	3.12	0.14	/	5.13	ICIM
173	SD	BLUE	*qSD7-5*	7	13,790,127	14,357,932	4.79	0.17	/	7.67	ICIM
174	SD	BLUE	*qSD8-16*	8	7,225,100	7,322,137	3.16	0.14	/	5.22	ICIM
175	SD	BLUE	*qSD8-17*	8	46,364,044	46,420,859	4.05	-0.16	/	6.58	ICIM
176	SD	BLUE	*qSD9-6*	9	36,249,643	36,545,817	4.66	0.17	/	7.56	ICIM
177	SD	BLUE	*qSD10-10*	10	45,243,194	45,322,107	12.56	0.27	/	18.88	ICIM
178	SD	BLUE	*qSD13-9*	13	44,813,128	44,886,577	2.24	0.12	/	3.73	ICIM
179	SD	BLUE	*qSD15-8*	15	11,812,679	11,838,874	1.80	0.10	/	3.01	ICIM
180	SD	BLUE	*qSD18-8*	18	49,063,398	49,310,631	1.92	-0.11	/	3.21	ICIM
181	SD	mean	*qSD2-15*	2	41,567,036	41,747,566	8.84	-0.26	/	12.96	ICIM
182	SD	mean	*qSD6-2*	6	44,555,867	44,964,228	1.98	0.13	/	3.07	ICIM
183	SD	mean	*qSD7-6*	7	13,790,127	14,357,932	3.75	0.17	/	5.50	ICIM

Serial No.	Trait	Environment	QTL	Chromosome	Start	End	PVE(%)	Add	P-value	LOD	Methods
184	SD	mean	*qSD9-7*	9	38,132,395	38,228,500	1.64	0.11	/	2.53	ICIM
185	SD	mean	*qSD10-11*	10	45,243,194	45,322,107	13.34	0.33	/	18.65	ICIM
186	SD	mean	*qSD14-1*	14	5,138,444	5,192,456	1.62	-0.11	/	2.54	ICIM
187	SD	mean	*qSD18-9*	18	48,264,142	48,367,672	3.27	-0.16	/	5.02	ICIM
188	BD	2019JZ	*qBD1-1*	1	3,561,373	3,678,971	3.81	0.14	/	6.60	ICIM
189	BD	2019JZ	*qBD4-6*	4	11,304,147	11,463,905	11.16	0.23	/	5.63	ICIM
190	BD	2019JZ	*qBD8-9*	8	0	417,244	1.65	0.09	/	3.01	ICIM
191	BD	2019JZ	*qBD8-10*	8	45,757,070	45,913,342	2.23	-0.10	/	3.50	ICIM
192	BD	2019JZ	*qBD10-10*	10	45,243,194	45,322,107	10.35	0.22	/	17.01	ICIM
193	BD	2019JZ	*qBD17-2*	17	11,938,709	12,105,510	2.06	0.10	/	3.72	ICIM
194	BD	2019SY	*qBD1-2*	1	51,053,492	51,488,812	3.58	0.08	/	5.90	ICIM
195	BD	2019SY	*qBD3-4*	3	6,710,177	13,132,697	14.38	0.16	/	6.51	ICIM
196	BD	2019SY	*qBD6-2*	6	17,622,676	17,705,666	3.43	0.08	/	5.76	ICIM
197	BD	2019SY	*qBD8-11*	8	7,108,611	7,136,283	2.91	0.07	/	4.89	ICIM
198	BD	2019SY	*qBD8-12*	8	47,525,385	47,621,113	4.07	-0.09	/	6.83	ICIM
199	BD	2019SY	*qBD15-5*	15	6,335,490	6,377,305	3.04	0.07	/	5.17	ICIM
200	BD	2020JZ	*qBD1-3*	1	3,561,373	3,678,971	2.95	0.07	/	3.68	ICIM
201	BD	2020JZ	*qBD6-3*	6	45,282,821	45,393,446	4.42	0.09	/	5.48	ICIM
202	BD	2020JZ	*qBD8-13*	8	7,225,100	7,322,137	4.34	0.09	/	5.29	ICIM
203	BD	2020JZ	*qBD8-14*	8	46,942,524	47,021,800	2.29	-0.06	/	2.90	ICIM
204	BD	2020JZ	*qBD9-5*	9	38,918,254	38,932,202	3.37	0.08	/	4.21	ICIM
205	BD	2020JZ	*qBD10-11*	10	44,487,017	44,548,961	10.11	0.13	/	12.01	ICIM
206	BD	BLUE	*qBD1-4*	1	3,561,373	3,678,971	4.55	0.07	/	6.32	ICIM

Serial No.	Trait	Environment	QTL	Chromosome	Start	End	PVE(%)	Add	P-value	LOD	Methods
207	BD	BLUE	*qBD1-5*	1	51,053,492	51,488,812	3.54	0.06	/	4.87	ICIM
208	BD	BLUE	*qBD2-5*	2	43,411,761	43,527,880	2.81	0.05	/	3.97	ICIM
209	BD	BLUE	*qBD8-15*	8	7,225,100	7,322,137	4.87	0.07	/	6.73	ICIM
210	BD	BLUE	*qBD10-12*	10	45,243,194	45,322,107	7.72	0.09	/	10.40	ICIM
211	BD	BLUE	*qBD15-6*	15	11,812,679	11,838,874	3.34	0.06	/	4.66	ICIM
212	BD	BLUE	*qBD16-2*	16	27,861,800	28,414,517	2.08	0.04	/	2.84	ICIM
213	BD	BLUE	*qBD20-3*	20	37,526,276	37,703,948	2.35	0.05	/	3.34	ICIM
214	BD	mean	*qBD1-6*	1	3,455,420	3,561,373	6.27	0.08	/	8.14	ICIM
215	BD	mean	*qBD1-7*	1	50,544,113	50,610,269	2.79	0.06	/	3.76	ICIM
216	BD	mean	*qBD2-6*	2	41,567,036	41,747,566	2.84	-0.06	/	3.86	ICIM
217	BD	mean	*qBD8-16*	8	7,225,100	7,322,137	3.53	0.06	/	4.72	ICIM
218	BD	mean	*qBD8-17*	8	46,942,524	47,021,800	5.02	-0.07	/	6.71	ICIM
219	BD	mean	*qBD10-13*	10	1,438,786	1,498,141	1.92	0.05	/	2.59	ICIM
220	BD	mean	*qBD10-14*	10	45,243,194	45,322,107	12.27	0.12	/	15.37	ICIM
221	BD	mean	*qBD17-3*	17	13,241,133	13,322,288	4.64	0.07	/	6.20	ICIM
222	BD	mean	*qBD20-4*	20	37,440,483	37,526,276	2.27	0.05	/	3.06	ICIM
223	SD	2019JZ	*qSD2-16*	2	44,394,468	44,450,267	5.97	0.35	/	4.82	3VmrMLM
224	SD	2019JZ	*qSD11-8*	11	26,779,181	26,793,921	4.38	-0.29	/	3.57	3VmrMLM
225	SD	2019SY	*qSD2-17*	2	42,012,035	42,097,139	6.81	-0.15	/	6.11	3VmrMLM
226	SD	2019SY	*qSD8-18*	8	46,395,913	46,420,859	8.42	-0.16	/	7.59	3VmrMLM
227	SD	2020JZ	*qSD10-12*	10	45,243,194	45,257,940	12.60	0.46	/	11.10	3VmrMLM
228	SD	2020JZ	*qSD11-9*	11	10,899,074	10,904,384	4.45	-0.27	/	4.07	3VmrMLM
229	SD	2021SQ	*qSD2-18*	2	5,351,331	5,371,539	6.71	0.29	/	5.14	3VmrMLM

Serial No.	Trait	Environment	QTL	Chromosome	Start	End	PVE(%)	Add	P-value	LOD	Methods
230	SD	BLUE	*qSD8-19*	8	46,395,913	46,420,859	6.42	-0.18	/	6.63	3VmrMLM
231	SD	BLUE	*qSD10-13*	10	45,209,617	45,243,194	8.91	0.24	/	8.89	3VmrMLM
232	SD	mean	*qSD2-19*	2	41,501,976	41,519,661	8.67	-0.26	/	8.59	3VmrMLM
233	SD	mean	*qSD5-6*	5	7,858,918	7,923,552	3.59	0.17	/	3.70	3VmrMLM
234	SD	mean	*qSD10-14*	10	41,520,177	41,675,137	4.76	0.05	/	5.14	3VmrMLM
235	SD	mean	*qSD10-15*	10	45,209,617	45,243,194	7.57	0.24	/	7.55	3VmrMLM
236	BD	2019JZ	*qBD8-18*	8	46,436,199	46,492,514	6.04	-0.12	/	5.89	3VmrMLM
237	BD	2019JZ	*qBD10-15*	10	45,243,194	45,257,940	11.03	0.20	/	10.10	3VmrMLM
238	BD	2019JZ	*qBD11-9*	11	10,904,384	11,043,519	4.21	-0.12	/	3.99	3VmrMLM
239	BD	2019SY	*qBD3-5*	3	8,022,822	12,188,240	5.51	0.09	/	6.72	3VmrMLM
240	BD	2019SY	*qBD6-4*	6	17,219,032	17,250,981	8.03	0.11	/	8.81	3VmrMLM
241	BD	2019SY	*qBD8-19*	8	46,395,913	46,420,859	5.57	-0.10	/	6.21	3VmrMLM
242	BD	2019SY	*qBD11-10*	11	24,983,624	25,055,183	11.24	0.13	/	12.36	3VmrMLM
243	BD	2020JZ	*qBD8-20*	8	47,021,800	47,061,716	5.40	-0.08	/	5.45	3VmrMLM
244	BD	2020JZ	*qBD10-16*	10	45,243,194	45,257,940	7.22	0.11	/	7.15	3VmrMLM
245	BD	2020JZ	*qBD17-4*	17	9,640,711	9,671,981	9.51	0.14	/	9.06	3VmrMLM
246	BD	BLUE	*qBD3-6*	3	13,115,549	13,132,697	5.87	0.08	/	4.92	3VmrMLM
247	BD	mean	*qBD3-7*	3	13,115,549	13,132,697	6.66	0.10	/	5.69	3VmrMLM

SD: stem diameter; BD: branch diameter; JZ: Jingzhou; SY: Sanya; SQ: Shangqiu; BLUE is the Best linear unbiased estimate; PVE is the phenotypic variation explained; ADD is the additive effects; LOD is the logarithm of odds; ICIM: inclusive composite interval mapping; RTM-GWAS: restricted two-stage multi-locus genome-wide association analysis; 3VmrMLM: three-variance component multi-locus random SNP effect mixed linear model.

Supplementary Table S4. Co-localized QTL identified by the same method in different environments

Method	Chromosome	QTL	Start	End	PVE(%)	Environment
RTM-GWAS	11	*qBD11-3*	10,904,384	11,043,519	2.29	2019JZ
		qBD11-5	10,779,406	10,904,384	7.24	2020JZ
ICIM	1	*qBD1-1*	3,561,373	3,678,971	3.81	2019JZ
		qBD1-3	3,561,373	3,678,971	2.95	2020JZ
3VmrMLM	10	*qBD10-15*	45,243,194	45,257,940	11.03	2019JZ
		qBD10-16	45,243,194	45,257,940	7.22	2020JZ

SD: stem diameter; BD: branch diameter; JZ: Jingzhou; RTM-GWAS: restricted two-stage multi-locus genome-wide association analysis; ICIM: inclusive composite interval mapping; 3VmrMLM: three-variance component multi-locus random SNP effect mixed linear model.

Supplementary Table S5. Co-localized QTL identified by different methods

Trait	Environment	Chromosome	RTM-GWAS.QTL	ICIM.QTL	3VmrMLM.QTL	Start	End
SD	2019SY	1	*qSD1-1*	*qSD1-3*	/	49,347,541	49,373,434
		7	*qSD7-1*	*qSD7-4*	/	14,012,455	14,094,620
		8	*qSD8-2*	*qSD8-14*	*qSD8-18*	46,364,044	46,518,573
		15	*qSD15-1*	*qSD15-7*	/	6,321,085	6,430,985
	2020JZ	10	*qSD10-4*	*qSD10-9*	*qSD10-12*	45,120,118	45,322,107
		11	*qSD11-2*	/	*qSD11-9*	10,779,406	10,904,384
BD	2019JZ	8	*qBD8-1*	/	*qBD8-18*	46,395,913	46,518,573
		10	/	*qBD10-10*	*qBD10-15*	45,243,194	45,322,107
		11	*qBD11-3*	/	*qBD11-9*	10,904,384	11,043,519
	2019SY	3	*qBD3-1*	*qBD3-4*	/	6,710,177	13,132,697
		3	/	*qBD3-4*	*qBD3-5*	6,710,177	13,132,697
		8	*qBD8-3*	*qBD8-12*	/	47,505,768	47,621,113
		11	*qBD11-4*	/	*qBD11-10*	24,983,624	25,055,183
	2020JZ	8	*qBD8-5*	/	*qBD8-20*	47,021,800	47,061,716
		10	*qBD10-5*	/	*qBD10-16*	45,120,118	45,257,940

SD: stem diameter; BD: branch diameter; JZ: Jingzhou; SY: Sanya; BLUE: best linear unbiased estimate; RTM-GWAS: restricted two-stage multi-locus genome-wide association analysis; ICIM: inclusive composite interval mapping; 3VmrMLM: three-variance component multi-locus random SNP effect mixed linear model.

Supplementary Table S6. Co-localized QTL by BLUE and mean inferred from different environments

Method	Trait	Chromosome	QTL1	QTL2	Start	End	Environment
RTM-GWAS	SD	10	*qSD10-4*	*qSD10-5*	45,120,118	45,257,940	2020JZ;BLUE
RTM-GWAS	SD	15	*qSD15-2*	*qSD15-4*	11,778,040	11,934,841	2020JZ;BLUE
RTM-GWAS	SD	20	*qSD20-2*	*qSD20-7*	44,666,415	44,746,133	2019JZ;BLUE
ICIM	SD	7	*qSD7-4*	*qSD7-5*	13,790,127	14,357,932	2019SY;BLUE
ICIM	SD	8	*qSD8-15*	*qSD8-16*	7,225,100	7,322,137	2020JZ;BLUE
ICIM	SD	8	*qSD8-14*	*qSD8-17*	46,364,044	46,420,859	2019SY;BLUE
ICIM	SD	9	*qSD9-5*	*qSD9-6*	36,249,643	36,545,817	2019SY;BLUE
ICIM	SD	10	*qSD10-9*	*qSD10-10*	45,243,194	45,322,107	2020JZ;BLUE
ICIM	BD	1	*qBD1-3*	*qBD1-4*	3,561,373	3,678,971	2020JZ;BLUE
ICIM	BD	1	*qBD1-1*	*qBD1-4*	3,561,373	3,678,971	2019JZ;BLUE
ICIM	BD	1	*qBD1-2*	*qBD1-5*	51,053,492	51,488,812	2019SY;BLUE
ICIM	BD	8	*qBD8-13*	*qBD8-15*	7,225,100	7,322,137	2020JZ;BLUE
ICIM	BD	10	*qBD10-10*	*qBD10-12*	45,243,194	45,322,107	2019JZ;BLUE
3VmrMLM	SD	8	*qSD8-18*	*qSD8-19*	46,395,913	46,420,859	2019SY;BLUE
RTM-GWAS	SD	2	*qSD2-5*	*qSD2-10*	41,501,976	41,519,661	2020JZ;mean
RTM-GWAS	SD	2	*qSD2-3*	*qSD2-9*	43,607,379	43,710,097	2019SY;mean
RTM-GWAS	SD	5	*qSD5-1*	*qSD5-4*	7,858,918	7,923,552	2019JZ;mean
RTM-GWAS	SD	8	*qSD8-2*	*qSD8-11*	46,364,044	46,518,573	2019SY;mean
RTM-GWAS	SD	10	*qSD10-4*	*qSD10-6*	45,120,118	45,257,940	2020JZ;mean

Method	Trait	Chromosome	QTL1	QTL2	Start	End	Environment
RTM-GWAS	BD	8	*qBD8-1*	*qBD8-8*	46,395,913	46,518,573	2019JZ;mean
RTM-GWAS	BD	13	*qBD13-1*	*qBD13-3*	37,025,399	37,175,077	2019JZ;mean
ICIM	SD	6	*qSD6-1*	*qSD6-2*	44,555,867	44,964,228	2020JZ;mean
ICIM	SD	7	*qSD7-4*	*qSD7-6*	13,790,127	14,357,932	2019SY;mean
ICIM	SD	10	*qSD10-9*	*qSD10-11*	45,243,194	45,322,107	2020JZ;mean
ICIM	SD	18	*qSD18-7*	*qSD18-9*	48,264,142	48,367,672	2020JZ;mean
ICIM	BD	8	*qBD8-13*	*qBD8-16*	7,225,100	7,322,137	2020JZ;mean
ICIM	BD	8	*qBD8-14*	*qBD8-17*	46,942,524	47,021,800	2020JZ;mean
ICIM	BD	10	*qBD10-10*	*qBD10-14*	45,243,194	45,322,107	2019JZ;mean

SD: stem diameter; BD: branch diameter; JZ: Jingzhou; SY: Sanya; SQ: Shangqiu; RTM-GWAS: restricted two-stage multi-locus genome-wide association analysis; ICIM: inclusive composite interval mapping; 3VmrMLM: three-variance component multi-locus random SNP effect mixed linear model.

Supplementary Table S7. Unique QTL for SD and BD

Trait	Chromosome	Start	End	QTL			
SD	1	53,117,378	53,159,107	*qSD1-2*			
SD	1	49,347,541	49,373,434	*qSD1-1*	*qSD1-3*		
SD	1	48,601,855	48,679,456	*qSD1-4*			
SD	2	27,201,003	27,381,884	*qSD2-1*	*qSD2-2*		
SD	2	41,501,976	41,519,661	*qSD2-5*	*qSD2-10*	*qSD2-19*	
SD	2	43,607,379	43,831,428	*qSD2-3*	*qSD2-8*	*qSD2-9*	*qSD2-12*
SD	2	44,394,468	44,450,267	*qSD2-16*			
SD	2	4,623,142	4,689,678	*qSD2-11*			
SD	2	42,012,035	42,097,139	*qSD2-17*			
SD	2	25,817,205	25,949,494	*qSD2-4*			
SD	2	47,621,618	47,712,802	*qSD2-6*			
SD	2	25,949,494	26,416,236	*qSD2-7*			
SD	2	4,898,292	5,026,152	*qSD2-13*			
SD	2	5,351,331	5,371,539	*qSD2-18*			
SD	2	43,411,761	43,527,880	*qSD2-14*			
SD	2	41,567,036	41,747,566	*qSD2-15*			
SD	3	36,518,347	36,708,811	*qSD3-1*			
SD	3	37,475,674	37,475,674	*qSD3-2*			
SD	4	50,667,694	50,690,238	*qSD4-1*			
SD	5	7,858,918	7,923,552	*qSD5-1*	*qSD5-4*	*qSD5-6*	
SD	5	24,803,769	27,738,758	*qSD5-3*	*qSD5-5*		
SD	5	23,236,127	23,236,127	*qSD5-2*			
SD	6	44,555,867	44,964,228	*qSD6-1*	*qSD6-2*		

Trait	Chromosome	Start	End	QTL						
SD	7	0	6,382,366	*qSD7-2*						
SD	7	19,828,550	19,839,096	*qSD7-3*						
SD	7	13,790,127	14,357,932	*qSD7-1*	*qSD7-4*	*qSD7-5*	*qSD7-6*			
SD	8	7,225,100	7,322,137	*qSD8-15*	*qSD8-16*					
SD	8	46,364,044	46,518,573	*qSD8-2*	*qSD8-10*	*qSD8-11*	*qSD8-14*	*qSD8-17*	*qSD8-18*	*qSD8-19*
SD	8	16,613,265	16,660,545	*qSD8-1*						
SD	8	1,676,370	1,812,439	*qSD8-3*						
SD	8	5,980,273	6,126,734	*qSD8-4*						
SD	8	46,168,008	46,239,110	*qSD8-5*						
SD	8	9,260,312	9,435,430	*qSD8-6*						
SD	8	36,400,556	37,040,512	*qSD8-7*						
SD	8	45,911,551	45,913,342	*qSD8-8*						
SD	8	39,878,906	39,917,603	*qSD8-9*						
SD	8	14,694,018	14,812,948	*qSD8-12*						
SD	8	39,697,455	39,878,906	*qSD8-13*						
SD	9	36,249,643	36,545,817	*qSD9-5*	*qSD9-6*					
SD	9	4,097,186	4,190,365	*qSD9-1*						
SD	9	19,082,826	19,301,463	*qSD9-2*						
SD	9	1,734,905	1,880,548	*qSD9-3*						
SD	9	28,312,724	28,312,724	*qSD9-4*						
SD	9	38,132,395	38,228,500	*qSD9-7*						
SD	10	5,347,014	6,926,536	*qSD10-1*						
SD	10	41,181,895	41,231,103	*qSD10-2*						
SD	10	45,475,505	45,622,801	*qSD10-3*						

Trait	Chromosome	Start	End	QTL								
SD	10	41,520,177	41,675,137	*qSD10-7*	*qSD10-14*							
SD	10	41,689,785	41,723,082	*qSD10-8*								
SD	10	45,120,118	45,322,107	*qSD10-4*	*qSD10-5*	*qSD10-6*	*qSD10-9*	*qSD10-10*	*qSD10-11*	*qSD10-12*	*qSD10-13*	*qSD10-15*
SD	11	11,043,519	11,043,519	*qSD11-4*	*qSD11-5*	*qSD11-6*						
SD	11	10,779,406	10,904,384	*qSD11-2*	*qSD11-9*							
SD	11	26,556,902	26,591,373	*qSD11-1*								
SD	11	26,779,181	26,793,921	*qSD11-8*								
SD	11	24,308,457	24,394,808	*qSD11-7*								
SD	11	31,936,739	31,953,512	*qSD11-3*								
SD	12	10,621,355	11,150,969	*qSD12-1*								
SD	12	36,287,746	36,347,559	*qSD12-2*								
SD	13	45,059,426	45,152,104	*qSD13-4*	*qSD13-5*							
SD	13	37,642,237	37,788,901	*qSD13-8*								
SD	13	28,337,047	28,710,672	*qSD13-1*								
SD	13	31,799,883	31,848,373	*qSD13-2*								
SD	13	43,447,381	43,447,381	*qSD13-3*								
SD	13	44,813,128	44,886,577	*qSD13-9*								
SD	13	37,145,616	37,175,077	*qSD13-6*								
SD	13	44,327,651	44,427,118	*qSD13-7*								
SD	14	5,138,444	5,192,456	*qSD14-1*								
SD	15	11,778,040	11,934,841	*qSD15-2*	*qSD15-4*	*qSD15-8*						
SD	15	6,321,085	6,430,985	*qSD15-1*	*qSD15-7*							
SD	15	6,982,474	7,021,640	*qSD15-3*								
SD	15	5,532,206	5,532,206	*qSD15-5*								

Trait	Chromosome	Start	End	QTL		
SD	15	6,889,315	6,982,474	*qSD15-6*		
SD	16	30,566,971	30,566,971	*qSD16-1*		
SD	17	2,526,930	2,664,000	*qSD17-1*		
SD	17	37,778,039	37,853,866	*qSD17-2*		
SD	18	48,264,142	48,367,672	*qSD18-6*	*qSD18-9*	*qSD18-7*
SD	18	19,303,346	19,430,527	*qSD18-1*		
SD	18	55,674,297	55,991,102	*qSD18-2*		
SD	18	15,940,594	16,188,577	*qSD18-3*		
SD	18	48,367,672	49,013,195	*qSD18-4*		
SD	18	49,063,398	49,310,631	*qSD18-8*		
SD	18	295,669	363,749	*qSD18-5*		
SD	19	20,727,913	23,101,391	*qSD19-1*		
SD	19	2,534,128	2,667,930	*qSD19-2*		
SD	19	4,506,176	4,506,176	*qSD19-3*		
SD	20	44,666,415	44,746,133	*qSD20-2*	*qSD20-7*	
SD	20	38,842,806	38,875,325	*qSD20-1*		
SD	20	37,208,897	37,335,219	*qSD20-3*		
SD	20	43,990,641	44,085,914	*qSD20-4*		
SD	20	44,775,256	44,986,065	*qSD20-9*		
SD	20	44,404,567	44,407,089	*qSD20-5*		
SD	20	37,811,887	37,811,887	*qSD20-6*		
SD	20	37,335,219	37,344,280	*qSD20-8*		
BD	1	3,561,373	3,678,971	*qBD1-1*	*qBD1-3*	*qBD1-4*
BD	1	51,053,492	51,488,812	*qBD1-2*	*qBD1-5*	

Trait	Chromosome	Start	End	QTL					
BD	1	3,455,420	3,561,373	*qBD1-6*					
BD	1	50,544,113	50,610,269	*qBD1-7*					
BD	2	39,493,768	39,703,135	*qBD2-1*					
BD	2	6,541,153	6,565,935	*qBD2-2*					
BD	2	46,277,482	46,336,665	*qBD2-3*					
BD	2	43,411,761	43,527,880	*qBD2-5*					
BD	2	25,817,205	25,949,494	*qBD2-4*					
BD	2	41,567,036	41,747,566	*qBD2-6*					
BD	3	6,710,177	13,132,697	*qBD3-1*	*qBD3-3*	*qBD3-4*	*qBD3-5*	*qBD3-6*	*qBD3-7*
BD	3	282,097	345,265	*qBD3-2*					
BD	4	48,403,516	48,519,846	*qBD4-1*					
BD	4	13,690,192	14,592,070	*qBD4-2*					
BD	4	19,000,528	19,935,526	*qBD4-3*					
BD	4	33,900,234	34,005,811	*qBD4-4*					
BD	4	11,304,147	11,463,905	*qBD4-6*					
BD	4	52,044,264	52,261,891	*qBD4-5*					
BD	5	2,215,956	2,385,119	*qBD5-2*					
BD	5	7,858,918	7,923,552	*qBD5-1*					
BD	5	0	584,541	*qBD5-3*					
BD	6	11,037,943	11,108,003	*qBD6-1*					
BD	6	45,282,821	45,393,446	*qBD6-3*					
BD	6	17,622,676	17,705,666	*qBD6-2*					
BD	6	17,219,032	17,250,981	*qBD6-4*					
BD	7	18,151,571	18,335,168	*qBD7-1*					

Trait	Chromosome	Start	End	QTL					
BD	7	39,446,389	39,484,113	*qBD7-2*					
BD	7	10,919,362	11,202,690	*qBD7-3*					
BD	8	7,225,100	7,322,137	*qBD8-13*	*qBD8-15*	*qBD8-16*			
BD	8	46,395,913	46,518,573	*qBD8-1*	*qBD8-8*	*qBD8-18*	*qBD8-19*		
BD	8	46,945,460	47,021,800	*qBD8-7*	*qBD8-14*	*qBD8-17*			
BD	8	47,021,800	47,061,716	*qBD8-5*	*qBD8-20*				
BD	8	47,505,768	47,621,113	*qBD8-3*	*qBD8-12*				
BD	8	17,599,584	17,848,472	*qBD8-2*					
BD	8	6,917,898	6,937,219	*qBD8-4*					
BD	8	0	417,244	*qBD8-9*					
BD	8	45,757,070	45,913,342	*qBD8-10*					
BD	8	7,108,611	7,136,283	*qBD8-11*					
BD	8	16,111,014	16,210,000	*qBD8-6*					
BD	9	37,983,058	38,139,379	*qBD9-3*					
BD	9	3,785,101	3,911,791	*qBD9-1*					
BD	9	3,911,791	3,976,497	*qBD9-2*					
BD	9	38,918,254	38,932,202	*qBD9-5*					
BD	9	45,059,701	45,072,261	*qBD9-4*					
BD	10	1,438,786	1,498,141	*qBD10-6*	*qBD10-13*				
BD	10	45,120,118	45,322,107	*qBD10-5*	*qBD10-10*	*qBD10-15*	*qBD10-16*	*qBD10-12*	*qBD10-14*
BD	10	46,072,021	46,210,329	*qBD10-2*					
BD	10	47,076,496	47,110,242	*qBD10-3*					
BD	10	45,475,505	45,622,801	*qBD10-1*					
BD	10	39,592,113	39,630,566	*qBD10-4*					

Trait	Chromosome	Start	End	QTL		
BD	10	44,487,017	44,548,961	*qBD10-11*		
BD	10	46,053,381	46,072,021	*qBD10-7*		
BD	10	45,658,686	45,697,530	*qBD10-8*		
BD	10	1,687,575	1,721,654	*qBD10-9*		
BD	11	10,779,406	11,043,519	*qBD11-3*	*qBD11-5*	*qBD11-9*
BD	11	24,983,624	25,055,183	*qBD11-4*	*qBD11-10*	
BD	11	8,739,403	9,363,712	*qBD11-1*		
BD	11	10,609,377	10,744,281	*qBD11-2*		
BD	11	24,823,012	24,983,624	*qBD11-6*		
BD	11	31,107,187	31,154,196	*qBD11-7*		
BD	11	25,264,762	25,439,066	*qBD11-8*		
BD	12	5,475,077	5,724,257	*qBD12-1*	*qBD12-3*	
BD	12	36,269,441	36,402,072	*qBD12-2*		
BD	13	37,025,399	37,175,077	*qBD13-1*	*qBD13-3*	
BD	13	38,041,203	38,067,451	*qBD13-2*		
BD	15	7,247,739	7,353,069	*qBD15-1*		
BD	15	12,253,558	12,304,443	*qBD15-2*		
BD	15	6,335,490	6,377,305	*qBD15-5*		
BD	15	10,827,709	10,860,771	*qBD15-3*		
BD	15	11,812,679	11,838,874	*qBD15-6*		
BD	15	8,539,141	8,649,553	*qBD15-4*		
BD	16	30,566,971	30,623,866	*qBD16-1*		
BD	16	27,861,800	28,414,517	*qBD16-2*		
BD	17	9,731,268	9,764,051	*qBD17-1*		

Trait	Chromosome	Start	End	QTL	
BD	17	11,938,709	12,105,510	*qBD17-2*	
BD	17	9,640,711	9,671,981	*qBD17-4*	
BD	17	13,241,133	13,322,288	*qBD17-3*	
BD	18	15,940,594	16,188,577	*qBD18-1*	
BD	18	48,179,723	48,200,966	*qBD18-2*	
BD	19	37,416,630	37,729,056	*qBD19-1*	
BD	19	38,058,638	38,188,500	*qBD19-2*	
BD	20	37,526,276	37,703,948	*qBD20-2*	*qBD20-3*
BD	20	1,855,970	2,013,720	*qBD20-1*	
BD	20	37,440,483	37,526,276	*qBD20-4*	

SD: stem diameter; BD: branch diameter.

Supplementary Table S8. Pleiotropic QTL discovered by three methods based on mean and BLUE

Method	Chromosome	SD.QTL	BD.QTL	Start	End	PVE(%)	Source
RTM-GWAS	5	*qSD5-1*	*qBD5-1*	7,858,918	7,923,552	2.66-4.46	2019JZ;2019JZ
RTM-GWAS	8	*qSD8-2*	*qBD8-1*	46,395,913	46,518,573	5.77-13.64	2019SY;2019JZ
RTM-GWAS	8	*qSD8-11*	*qBD8-8*	46,364,044	46,518,573	5.79-10.28	mean;mean
RTM-GWAS	10	*qSD10-4*	*qBD10-5*	45,120,118	45,257,940	15.11-19.61	2020JZ;2020JZ
RTM-GWAS	10	*qSD10-3*	*qBD10-1*	45,475,505	45,622,801	2.98-17.46	2019JZ;2019JZ
RTM-GWAS	11	*qSD11-2*	*qBD11-3/qBD11-5*	10,779,406	11,043,519	2.29-7.24	2020JZ;2019JZ;2020JZ
RTM-GWAS	13	*qSD13-6*	*qBD13-3*	37,145,616	37,175,077	2.51-3.32	mean;mean
RTM-GWAS	18	*qSD18-3*	*qBD18-1*	15,940,594	16,188,577	3.38-4.02	2020JZ;2020JZ
ICIM	2	*qSD2-14*	*qBD2-5*	43,411,761	43,527,880	2.81-3.12	BLUE;BLUE
ICIM	2	*qSD2-15*	*qBD2-6*	41,567,036	41,747,566	2.84-8.84	mean;mean
ICIM	8	*qSD8-15*	*qBD8-13*	7,225,100	7,322,137	2.16-4.34	2020JZ;2020JZ
ICIM	8	*qSD8-16*	*qBD8-15*	7,225,100	7,322,137	3.16-4.87	BLUE;BLUE
ICIM	10	*qSD10-9*	*qBD10-10*	45,243,194	45,322,107	10.35-15.96	2020JZ;2019JZ
ICIM	10	*qSD10-10*	*qBD10-12*	45,243,194	45,322,107	7.72-12.56	BLUE;BLUE
ICIM	10	*qSD10-11*	*qBD10-14*	45,243,194	45,322,107	12.27-13.34	mean;mean
ICIM	15	*qSD15-7*	*qBD15-5*	6,335,490	6,377,305	3.04-3.70	2019SY;2019SY
ICIM	15	*qSD15-8*	*qBD15-6*	11,812,679	11,838,874	1.80-3.34	BLUE;BLUE
3VmrMLM	8	*qSD8-18*	*qBD8-19*	46,395,913	46,420,859	5.57-8.42	2019SY;2019SY
3VmrMLM	10	*qSD10-12*	*qBD10-15/qBD10-16*	45,243,194	45,257,940	7.22-12.60	2020JZ;2019JZ;2020JZ
3VmrMLM	11	*qSD11-9*	*qBD11-9*	10,899,074	11,043,519	4.21-4.45	2020JZ;2019JZ

SD: stem diameter; BD: branch diameter; JZ: Jingzhou; SY: Sanya; SQ: Shangqiu; RTM-GWAS: restricted two-stage multi-locus genome-wide association analysis; ICIM: inclusive composite interval mapping; 3VmrMLM: three-variance component multi-locus random SNP effect mixed linear model.

Supplementary Table S9. Genetic differentiation index (F_{ST}) of genes related to SD and BD

Gene	Chromosome	Start	End	F_{ST}
Glyma.01G034400	1	3,582,060	3,583,811	0.60
Glyma.02G246400	2	43,441,160	43,443,049	0.66
Glyma.02G246700	2	43,465,963	43,468,829	0.76
Glyma.02G246800	2	43,469,911	43,470,390	0.63
Glyma.02G247000	2	43,478,314	43,481,473	0.66
Glyma.02G247100	2	43,492,461	43,494,623	0.86
Glyma.04G107500	4	11,317,835	11,319,777	0.65
Glyma.05G071700	5	7,860,560	7,861,720	0.86
Glyma.05G071800	5	7,868,215	7,871,153	0.77
Glyma.05G071900	5	7,885,012	7,885,414	0.74
Glyma.07G121000	7	14,023,351	14,029,811	0.77
Glyma.07G121100	7	14,029,254	14,029,813	0.74
Glyma.08G350100	8	46,412,654	46,414,035	0.64
Glyma.08G350300	8	46,418,952	46,419,101	0.63
Glyma.08G350400	8	46,419,936	46,424,538	0.65
Glyma.08G351000	8	46,466,172	46,470,851	0.69
Glyma.08G351700	8	46,498,600	46,499,878	0.70
Glyma.08G351900	8	46,509,706	46,516,703	0.63
Glyma.08G363600	8	47,512,398	47,519,238	0.78
Glyma.08G363700	8	47,520,208	47,526,611	0.64
Glyma.08G364200	8	47,553,392	47,555,880	0.72
Glyma.08G364300	8	47,556,171	47,558,975	0.72
Glyma.08G364500	8	47,563,535	47,564,496	0.85

Gene	Chromosome	Start	End	F_{ST}
Glyma.08G364900	8	47,581,368	47,582,279	0.68
Glyma.08G365000	8	47,602,097	47,606,318	0.88
Glyma.08G095000	8	7,230,311	7,239,978	0.73
Glyma.08G095200	8	7,245,023	7,250,047	0.76
Glyma.08G095300	8	7,258,432	7,260,587	0.76
Glyma.08G095400	8	7,267,235	7,270,875	0.70
Glyma.08G095500	8	7,271,630	7,272,544	0.70
Glyma.08G095800	8	7,304,924	7,308,800	0.73
Glyma.10G181900	10	41,531,674	41,535,236	0.87
Glyma.10G182000	10	41,536,839	41,546,196	0.66
Glyma.10G182300	10	41,561,515	41,570,949	0.64
Glyma.10G182800	10	41,590,798	41,594,394	0.64
Glyma.10G182900	10	41,602,272	41,604,035	0.68
Glyma.10G183000	10	41,605,748	41,617,854	0.63
Glyma.10G183200	10	41,621,187	41,628,008	0.66
Glyma.10G183300	10	41,633,437	41,645,279	0.64
Glyma.10G183400	10	41,657,981	41,659,143	0.62
Glyma.10G183500	10	41,664,447	41,669,277	0.67
Glyma.10G219600	10	45,126,440	45,130,601	0.65
Glyma.10G219700	10	45,134,028	45,138,735	0.62
Glyma.10G219800	10	45,139,887	45,141,455	0.68
Glyma.10G219900	10	45,144,422	45,147,283	0.67
Glyma.10G220000	10	45,149,408	45,157,038	0.67
Glyma.10G220100	10	45,160,713	45,166,890	0.74

Gene	Chromosome	Start	End	F_{ST}
Glyma.10G220700	10	45,193,087	45,207,808	0.61
Glyma.10G221000	10	45,225,655	45,239,057	0.61
Glyma.10G221300	10	45,266,945	45,274,167	0.70
Glyma.10G224000	10	45,479,863	45,493,565	0.61
Glyma.10G224100	10	45,494,198	45,496,872	0.65
Glyma.10G224200	10	45,498,206	45,500,186	0.68
Glyma.10G224300	10	45,503,642	45,505,214	0.65
Glyma.10G224400	10	45,509,340	45,510,452	0.72
Glyma.10G224500	10	45,514,702	45,516,815	0.68
Glyma.10G224700	10	45,534,516	45,534,867	0.70
Glyma.10G225300	10	45,598,119	45,603,561	0.69
Glyma.10G225400	10	45,605,390	45,609,815	0.67
Glyma.10G230700	10	46,060,022	46,065,739	0.82
Glyma.10G230800	10	46,065,779	46,068,770	0.80
Glyma.11G141200	11	10,783,630	10,788,473	0.77
Glyma.11G141300	11	10,790,718	10,795,368	0.76
Glyma.11G141800	11	10,825,602	10,826,647	0.72
Glyma.11G141900	11	10,832,090	10,832,873	0.68
Glyma.11G142000	11	10,833,308	10,833,493	0.66
Glyma.11G142100	11	10,835,513	10,837,136	0.67
Glyma.11G142600	11	10,872,527	10,876,330	0.82
Glyma.11G143000	11	10,919,661	10,923,789	0.70
Glyma.11G143100	11	10,924,084	10,925,772	0.61
Glyma.11G143700	11	10,972,434	10,973,523	0.62

Gene	Chromosome	Start	End	F_{ST}
Glyma.11G143800	11	10,973,787	10,976,060	0.64
Glyma.11G144100	11	10,994,674	10,996,460	0.71
Glyma.11G182600	11	24,998,933	25,003,549	0.72
Glyma.15G143600	15	11,790,380	11,793,024	0.69
Glyma.15G143700	15	11,812,631	11,819,435	0.63
Glyma.15G143800	15	11,824,186	11,826,677	0.61
Glyma.15G143900	15	11,830,914	11,834,773	0.71
Glyma.15G144000	15	11,843,111	11,847,542	0.69
Glyma.15G144100	15	11,848,711	11,854,145	0.72
Glyma.15G144200	15	11,858,768	11,859,643	0.70
Glyma.15G144300	15	11,871,920	11,872,232	0.80
Glyma.15G082900	15	6,333,075	6,334,762	0.69
Glyma.15G083000	15	6,340,563	6,342,031	0.72
Glyma.15G083200	15	6,359,940	6,361,430	0.62
Glyma.15G083300	15	6,364,062	6,368,149	0.68
Glyma.15G083400	15	6,369,497	6,370,996	0.66
Glyma.15G083500	15	6,381,987	6,386,260	0.67
Glyma.15G083600	15	6,390,214	6,394,961	0.75
Glyma.15G083800	15	6,412,945	6,419,216	0.63
Glyma.18G123600	18	16,015,169	16,021,694	0.60
Glyma.20G136900	20	37,650,802	37,653,939	0.65

F_{ST}: fixation index.

Supplementary Table S10. Functional annotation of 55 candidate genes

Trait	Soybean genes	Arabidopsis genes	Functional annotation
SD	*Glyma.07G121000*	*AT5G26667.1*	P-loop containing nucleoside triphosphate hydrolases superfamily protein
SD	*Glyma.07G121100*		
SD	*Glyma.10G181900*	*AT1G19800.1*	trigalactosyldiacylglycerol 1
SD	*Glyma.10G182000*	*AT3G01810.1*	
SD	*Glyma.10G182300*	*AT5G43310.3*	COP1-interacting protein-related
SD	*Glyma.10G182800*	*AT4G14330.1*	P-loop containing nucleoside triphosphate hydrolases superfamily protein
SD	*Glyma.10G183000*	*AT3G23410.1*	fatty alcohol oxidase 3
SD	*Glyma.10G183200*	*AT4G22340.3*	cytidinediphosphate diacylglycerol synthase 2
SD	*Glyma.10G183500*	*AT4G14305.1*	Peroxisomal membrane 22 kDa (Mpv17/PMP22) family protein
SD	*Glyma.15G082900*	*AT3G58630.1*	sequence-specific DNA binding transcription factors
SD	*Glyma.15G083500*	*AT1G29240.1*	Protein of unknown function (DUF688)
SD	*Glyma.15G083600*	*AT2G38300.1*	myb-like HTH transcriptional regulator family protein
SD	*Glyma.15G143600*	*AT3G16920.1*	chitinase-like protein 2
SD	*Glyma.15G144100*	*AT3G58650.1*	
BD	*Glyma.04G107500*	*AT3G09270.1*	glutathione S-transferase TAU 8
BD	*Glyma.08G363600*	*AT5G47900.1*	Protein of unknown function (DUF1624)
BD	*Glyma.08G363700*	*AT1G29880.1*	glycyl-tRNA synthetase / glycine--tRNA ligase
BD	*Glyma.08G364500*	*AT2G43780.1*	
BD	*Glyma.08G365000*	*AT5G38450.1*	cytochrome P450, family 735, subfamily A, polypeptide 1
BD	*Glyma.10G230700*	*AT5G52660.1*	Homeodomain-like superfamily protein
BD	*Glyma.11G182600*	*AT5G22400.1*	Rho GTPase activating protein with PAK-box/P21-Rho-binding domain
BD	*Glyma.20G136900*	*AT4G33740.1*	
pleiotropic	*Glyma.02G246800*		

Trait	Soybean genes	Arabidopsis genes	Functional annotation
pleiotropic	*Glyma.02G247000*	*AT5G37840.1*	
pleiotropic	*Glyma.08G095000*	*AT2G01600.1*	ENTH/ANTH/VHS superfamily protein
pleiotropic	*Glyma.08G095200*	*AT1G23170.2*	Protein of unknown function DUF2359, transmembrane
pleiotropic	*Glyma.08G095400*	*AT2G44510.1*	CDK inhibitor P21 binding protein
pleiotropic	*Glyma.08G095800*	*AT2G01570.1*	GRAS family transcription factor family protein
pleiotropic	*Glyma.08G351000*	*AT3G07130.1*	purple acid phosphatase 15
pleiotropic	*Glyma.08G351700*	*AT3G24100.1*	Uncharacterised protein family SERF
pleiotropic	*Glyma.08G351900*	*AT5G59540.1*	2-oxoglutarate (2OG) and Fe(II)-dependent oxygenase superfamily protein
pleiotropic	*Glyma.10G219600*	*AT1G34190.1*	NAC domain containing protein 17
pleiotropic	*Glyma.10G219700*	*AT1G14290.1*	sphingoid base hydroxylase 2
pleiotropic	*Glyma.10G220000*	*AT1G34150.1*	Pseudouridine synthase family protein
pleiotropic	*Glyma.10G220100*	*AT4G09720.1*	RAB GTPase homolog G3A
pleiotropic	*Glyma.10G220700*	*AT1G34110.1*	Leucine-rich receptor-like protein kinase family protein
pleiotropic	*Glyma.10G221000*	*AT1G71710.1*	DNAse I-like superfamily protein
pleiotropic	*Glyma.10G224000*	*AT1G43620.1*	UDP-Glycosyltransferase superfamily protein
pleiotropic	*Glyma.10G224100*	*AT1G76760.1*	thioredoxin Y1
pleiotropic	*Glyma.10G224200*	*AT1G22780.1*	Ribosomal protein S13/S18 family
pleiotropic	*Glyma.10G224300*		
pleiotropic	*Glyma.10G224500*	*AT1G18740.1*	Protein of unknown function (DUF793)
pleiotropic	*Glyma.10G225300*	*AT1G21200.1*	sequence-specific DNA binding transcription factors
pleiotropic	*Glyma.11G141200*	*AT2G28760.1*	UDP-XYL synthase 6
pleiotropic	*Glyma.11G141300*	*AT3G46440.1*	UDP-XYL synthase 5
pleiotropic	*Glyma.11G141800*	*AT3G45980.1*	Histone superfamily protein
pleiotropic	*Glyma.11G142100*	*AT3G45980.1*	Histone superfamily protein

Trait	Soybean genes	Arabidopsis genes	Functional annotation
pleiotropic	*Glyma.11G142600*	*AT5G59790.1*	Domain of unknown function (DUF966)
pleiotropic	*Glyma.11G143000*	*AT5G59770.1*	Protein-tyrosine phosphatase-like, PTPLA
pleiotropic	*Glyma.11G143100*	*AT4G01470.1*	tonoplast intrinsic protein 1;3
pleiotropic	*Glyma.15G083000*	*AT1G29195.1*	
pleiotropic	*Glyma.15G083300*	*AT5G64270.1*	splicing factor, putative
pleiotropic	*Glyma.15G143700*	*AT5G49360.1*	beta-xylosidase 1
pleiotropic	*Glyma.15G143900*	*AT5G49350.1*	Glycine-rich protein family
pleiotropic	*Glyma.18G123600*	*AT2G02820.2*	myb domain protein 88

SD: stem diameter; BD: branch diameter.
