# Supplementary figures and images for: QTL mapping and genomic selection of stem and branch diameter in soybean (Glycine max L.)
Source: Front Plant Sci. 2024 May 31;15:1388365. doi: 10.3389/fpls.2024.1388365 (PMC11176531; doi:10.3389/fpls.2024.1388365)

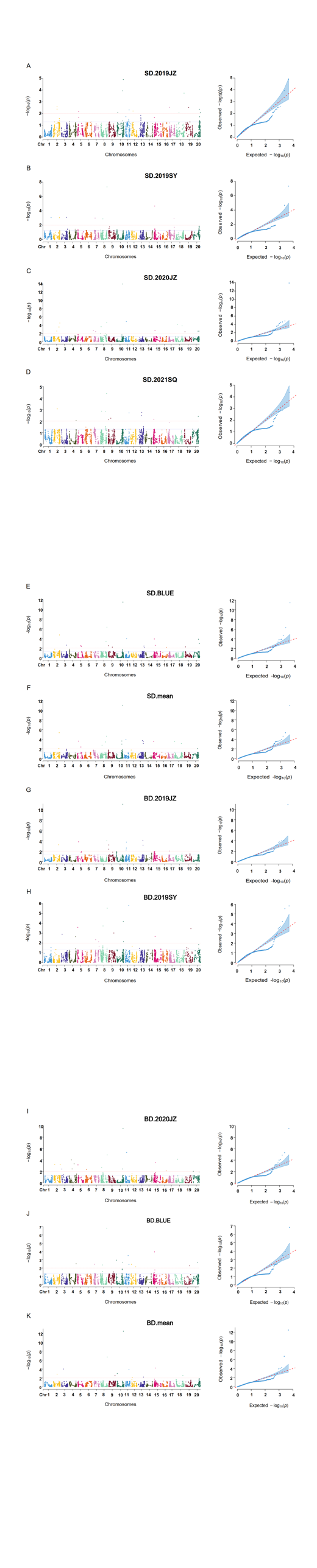

Supplement: Supplementary file 1 [file DataSheet_1.zip › Supplementary Material/Supplementary Figures tif.version/Fig S1.tif]

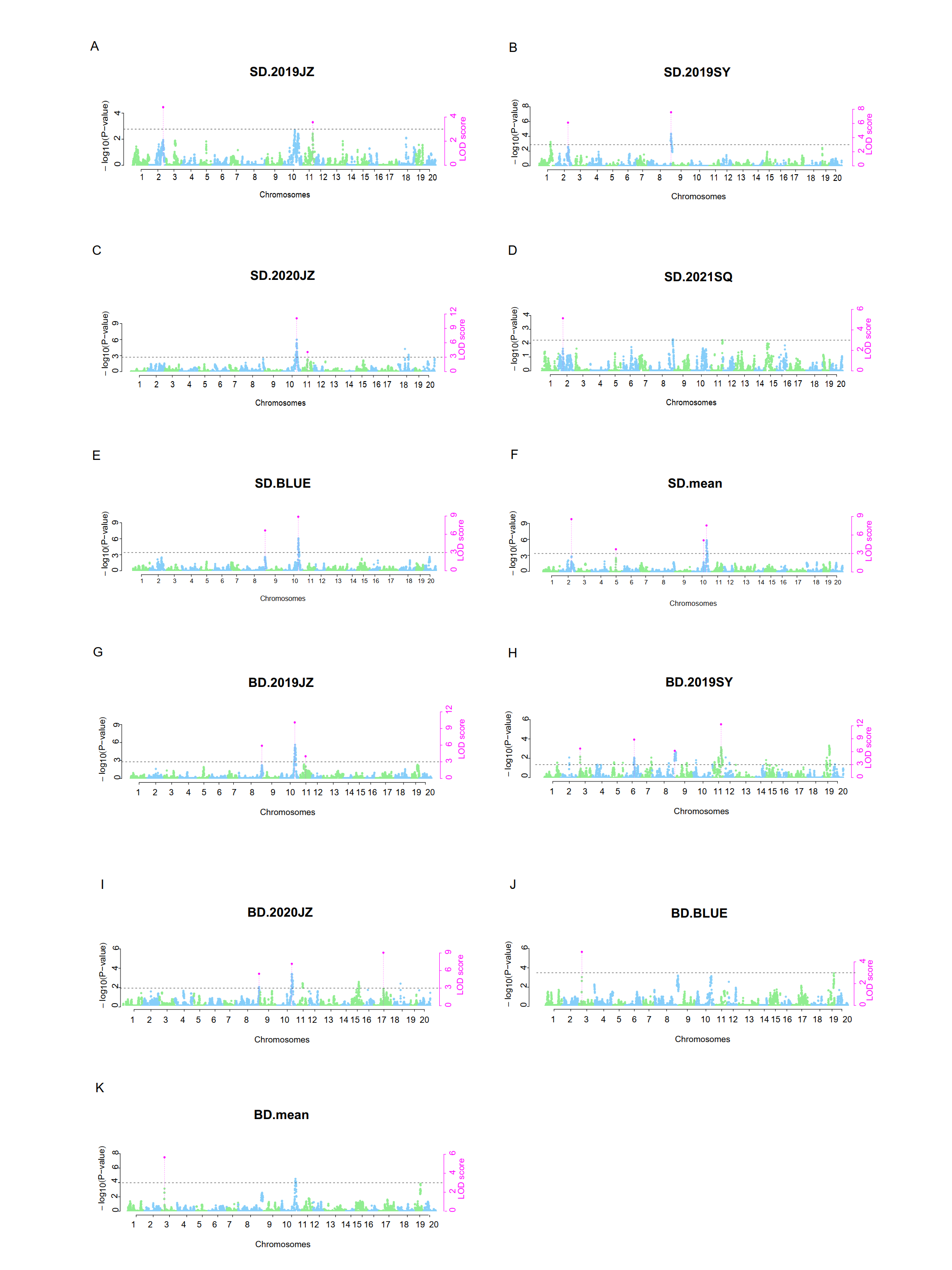

Supplement: Supplementary file 1 [file DataSheet_1.zip › Supplementary Material/Supplementary Figures tif.version/Fig S2.tif]

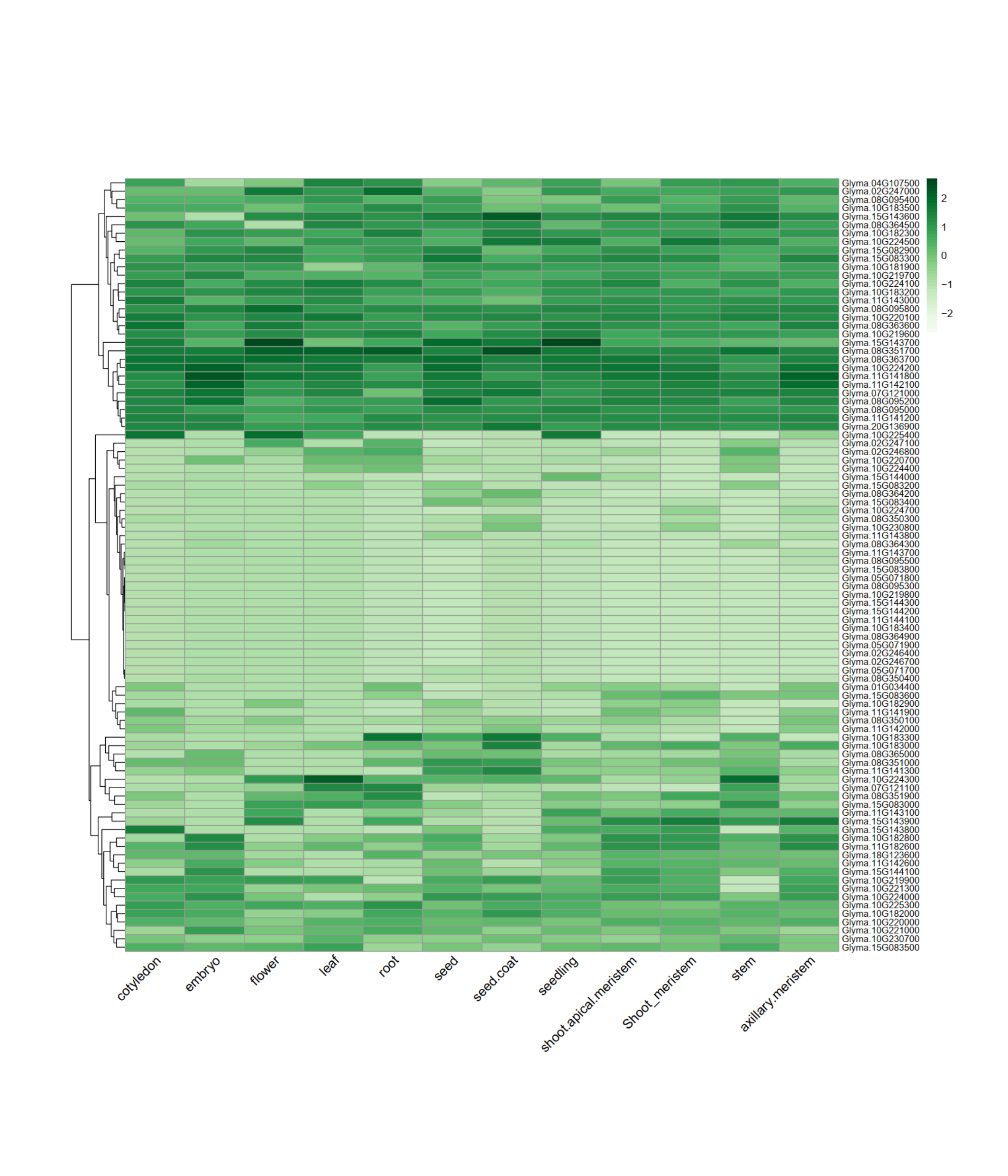

Supplement: Supplementary file 1 [file DataSheet_1.zip › Supplementary Material/Supplementary Figures tif.version/Fig S3.tif]
